# Macrooxazoles A–D, New 2,5-Disubstituted Oxazole-4-Carboxylic Acid Derivatives from the Plant Pathogenic Fungus *Phoma macrostoma*

**DOI:** 10.3390/molecules25235497

**Published:** 2020-11-24

**Authors:** Blondelle Matio Kemkuignou, Laura Treiber, Haoxuan Zeng, Hedda Schrey, Rainer Schobert, Marc Stadler

**Affiliations:** 1 Department of Microbial Drugs, Helmholtz Centre for Infection Research GmbH, Inhoffenstrasse 7, 38124 Braunschweig, Germany; blondelle.matiokemkuignou@helmholtz-hzi.de (B.M.K.); haoxuan.zeng@helmholtz-hzi.de (H.Z.); hedda.schrey@helmholtz-hzi.de (H.S.); 2 German Centre for Infection Research (DZIF), partner site Hannover-Braunschweig, Inhoffenstrasse 7, 38124 Braunschweig, Germany; 3 Organic chemistry laboratory, University of Bayreuth, Universitaetsstrasse 30, 95447 Bayreuth, Germany; Laura1.Treiber@uni-bayreuth.de (L.T.); Rainer.Schobert@uni-bayreuth.de (R.S.)

**Keywords:** *Phoma macrostoma*, oxazole derivatives, anti-biofilm, isolation, structure elucidation, macrocidin Z synthesis

## Abstract

In our ongoing search for new bioactive fungal metabolites, four previously undescribed oxazole carboxylic acid derivatives (**1**–**4**) for which we proposed the trivial names macrooxazoles A–D together with two known tetramic acids (**5**–**6**) were isolated from the plant pathogenic fungus *Phoma macrostoma*. Their structures were elucidated based on high-resolution mass spectrometry (HR-MS) and nuclear magnetic resonance (NMR) spectroscopy. The hitherto unclear structure of macrocidin Z (**6**) was also confirmed by its first total synthesis. The isolated compounds were evaluated for their antimicrobial activities against a panel of bacteria and fungi. Cytotoxic and anti-biofilm activities of the isolates are also reported herein. The new compound **3** exhibited weak-to-moderate antimicrobial activity as well as the known macrocidins **5** and **6**. Only the mixture of compounds **2** and **4** (ratio 1:2) showed weak cytotoxic activity against the tested cancer cell lines with an IC_50_ of 23 µg/mL. Moreover, the new compounds **2** and **3**, as well as the known compounds **5** and **6**, interfered with the biofilm formation of *Staphylococcus aureus*, inhibiting 65%, 75%, 79%, and 76% of biofilm at 250 µg/mL, respectively. Compounds **5** and **6** also exhibited moderate activity against *S. aureus* preformed biofilm with the highest inhibition percentage of 75% and 73% at 250 µg/mL, respectively.

## 1. Introduction

Oxazole and its derivatives are heterocyclic systems which have gained strong interest in recent times due to their increasing importance in the field of medicinal chemistry [1]. They feature a well-known important doubly unsaturated 5-membered ring heterocyclic motif having one oxygen atom at position 1 and a nitrogen at position 3 separated by a carbon in between [2]. Their widespread useful biological activities including antimicrobial [3], anticancer [4], antitubercular [5], anti-inflammatory [6], antidiabetic [7], antiobesity [8] and anthelminthic [9] effects have attracted increasing attention of chemical and pharmacological communities in their search for new lead compounds [1]. Some of them have shown promising therapeutic potential and have qualified for both preclinical and clinical evaluations [2]. Previous studies reported the isolation of several biologically active substituted oxazole-containing natural products mostly from marine invertebrates and microorganisms [2,10,11]. For instance, hennoxazole A, isolated from a marine sponge *Polyfibrospongia* sp., was reported to possess antiviral activity [12,13] while the phthoxazolins isolated from *Streptomyces* sp. showed selective activity against the oomycete *Phytophthora parasitica* in vitro [14]. As part of our ongoing search of exploring fungi for new biologically active metabolites, we investigated the chemical components of the fermentation extract of the plant pathogenic fungus *Phoma macrostoma* originally isolated from its host, the noxious weed, *Cirsium arvense*. Previous investigations indicated that the liquid culture of the fungus could produce phytotoxic metabolites named macrocidins, which also caused bleaching when applied foliarly to several dicotyledonous species [15,16]. In the present work, four previously undescribed oxazole-4-carboxylic acid derivatives (**1**–**4**) together with two known macrocidins (**5**–**6**) were isolated from the liquid culture of *Phoma macrostoma*. The structures of the isolates were elucidated by means of high resolution electro spray ionization mass spectrometry (HR-ESIMS) data and 1D and 2D nuclear magnetic resonance (NMR) spectroscopic data. The so far contentious structure of macrocidin Z (**6**) was also confirmed by comparison of the isolate with the product of its first total synthesis. All compounds were investigated for antimicrobial and cytotoxic effects. The current paper reports details of their isolation, structural elucidation and biological activities.

## 2. Results and Discussion

### 2.1. Structure Elucidation of Compounds ***1**–**6***

**Figure 1:**
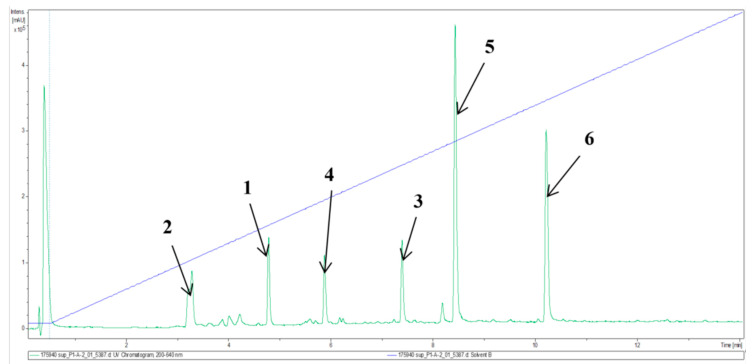
Analytical HPLC–UV/Vis chromatogram of the crude extract from the supernatant of *Phoma macrostoma* (diode array detection at 200–640 nm). Stationary phase: C18 Acquity UPLC BEH column; for gradient and other details on the experimental setup, see the Experimental section; **1**–**6**: Major metabolites detected (chemical structures see Figure 2). % solvent B (acetonitrile (ACN) + 0.1% formic acid 5–100%). Gradient is indicated by the blue line.

**Figure 2:**
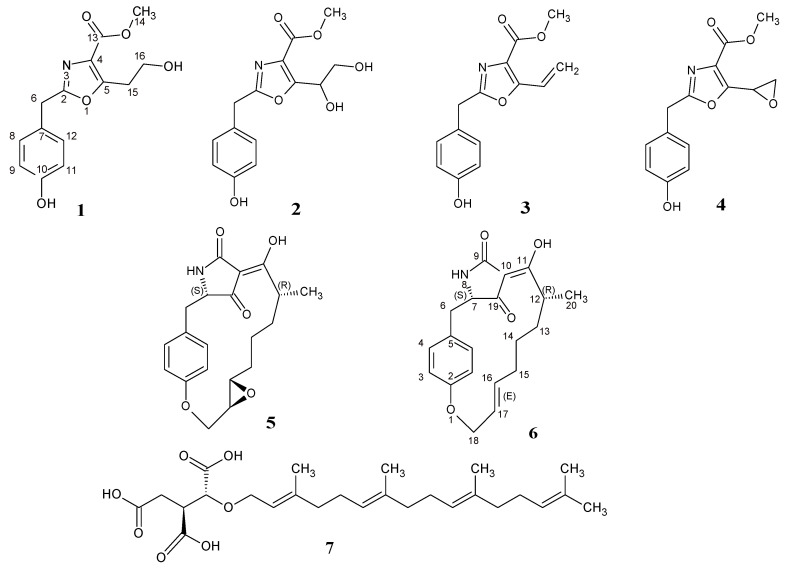
Chemical structures of secondary metabolites isolated from *Phoma macrostoma* (**1**–**6**) and the known biofilm inhibitor microporenic acid A (**7**).

**Figure 3:**
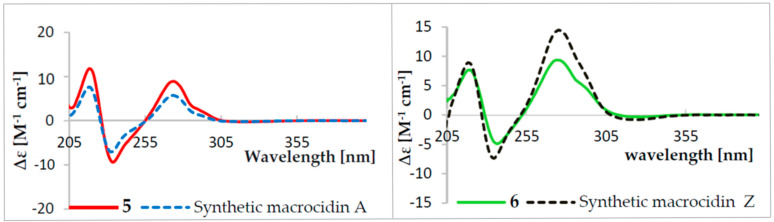
ECD spectra of isolated and synthetic macrocidins A (**5**) and Z (**6**) in MeOH.

**Scheme 1:**
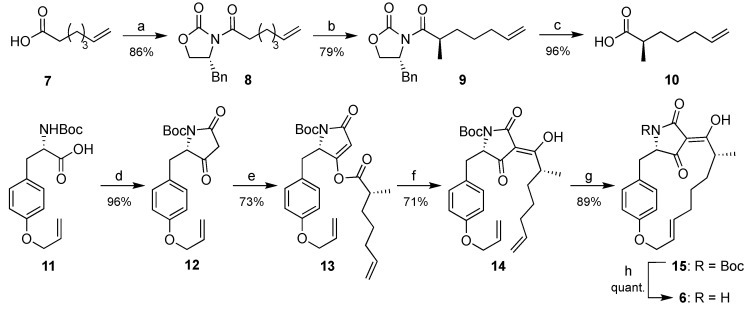
Synthesis of macrocidin Z **(6).** Reagents and conditions: (a) DCC, DMAP, (*R*)-benzyl-2-oxazolidinone, CH_2_Cl_2_, 23 h; (b) 1. NaHMDS, THF, −78 °C, 30 min, 2. MeI, 4.5 h; (c) LiOH, H_2_O_2_, THF/H_2_O (2:1); (d) Meldrum´s acid, DMAP, EDC∙HCl, CH_2_Cl_2_, rt, 2 h; (e) **10**, DMAP, EDC∙HCl, CH_2_Cl_2_, 0 °C, rt, 2 h; (f) NEt_3_, DMAP, CH_2_Cl_2_, rt, 24 h; (g) Grubbs II catalyst, CH_2_Cl_2_, ∆, 15 h; (h) TFA, CH_2_Cl_2_, rt, 15 min. DCC = dicyclohexylcarbodiimide; DMAP = dimethylaminopyridine; NaHMDS = sodium hexamethyldisilazanide; THF = tetrahydrofuran; EDC = 1-ethyl-3-(3-dimethylaminopropyl)carbodiimide; TFA = trifluoroacetic acid.

Liquid fermentation in Q6 ½ medium of *Phoma macrostoma* was carried out as described in the Materials and Methods section. The major metabolites were detected by analytical high performance liquid chromatography (HPLC) as shown in Figure 1 and the preparative HPLC separation to obtain the pure metabolites was guided accordingly. Fractionation of the crude extract using reverse-phase HPLC led to the isolation of four previously undescribed metabolites (**1**–**4**), together with two known compounds, the macrocidins A (**5**) and Z (**6**) (Figure 2). Macrocidin A (**5**) was identified by comparing its NMR and HR-ESIMS data with those reported in the literature [15]. Its absolute configuration was confirmed to be identical to that of the synthetic macrocidin A [17] by comparison of their respective Electronic Circular Dichroism (ECD) spectra (Figure 3). As the structure of macrocidin Z (**6**) was unclear so far [16], we synthesized it for the first time (Scheme 1) and found a perfect match of the ^1^H and ^13^C-NMR data, as well as of the ECD spectra of the synthetic and the isolated macrocidin Z samples (see Table 1 and Figure 3). The E-geometry of the Δ^16–17^ double bond in macrocidin Z was determined based on the existence of a coupling constant *J* = 15.5 Hz between H-16 and H-17.

Compound **1** was isolated as a yellow oil from both the supernatant and the mycelia. Its molecular formula was established as C_14_H_15_NO_5_ (8 degrees of unsaturation) based on its [M + H]^+^ ion at *m*/*z* 278.1026 and [M + Na]^+^ ion at *m*/*z* 300.0837 in the HR-ESIMS.

The ^1^H-NMR spectroscopic data coupled to the ^1^H-^1^H correlation spectroscopy (^1^H-^1^H COSY) spectrum revealed two doublets resonating at *δ* 7.10 (H-8/H-12, d, 9.0) and *δ* 6.73 (H-9/H-11, d, 9.0) integrating for two aromatic protons each, suggesting a 1,4-disubstituted aromatic ring. One methoxy group singlet resonating at *δ* 3.87 (H-14, s), a singlet methylene resonating at *δ* 4.00 (H-6, s), a triplet for methylene protons resonating at *δ* 3.19 (H-15, t, 6.5), and linked to an oxygenated methylene at *δ* 3.80 (H-16, t, 6.5) were also recorded.

The ^13^C-NMR spectrum showed 12 carbon signals instead of 14 as indicated by the molecular formula suggesting the presence of symmetrical carbons and thus confirming the existence of a 1,4-disubstituted aromatic ring. The 12 carbons were further identified as one methoxy, two methylene, one oxymethylene, two aromatic methine carbons and six non-protonated sp^2^ carbons from detailed analysis of its ^1^H-^13^C heteronuclear single quantum coherence (^1^H-^13^C HSQC) spectrum (Table 2). The gross structure of **1** was determined by comprehensive analysis of its 2D NMR including the COSY, HMBC (heteronuclear multiple bond correlation) and NOESY (nuclear overhauser effect spectroscopy) spectra. The chemical shifts of the aromatic carbons as well as the HMBC correlations of H-8/H-12 to C-10 (*δ* 157.9)/C-6 (*δ* 34.3), H-9/H-11 to C-10 (*δ* 157.9)/C-7 (*δ* 127.0) and H-6 to C-7(*δ* 127.0)/C-8(*δ* 131.0) indicated a benzyl group with an oxygen substitution para to the methylene leading to a para-hydroxybenzyl moiety. 

**Figure 4:**
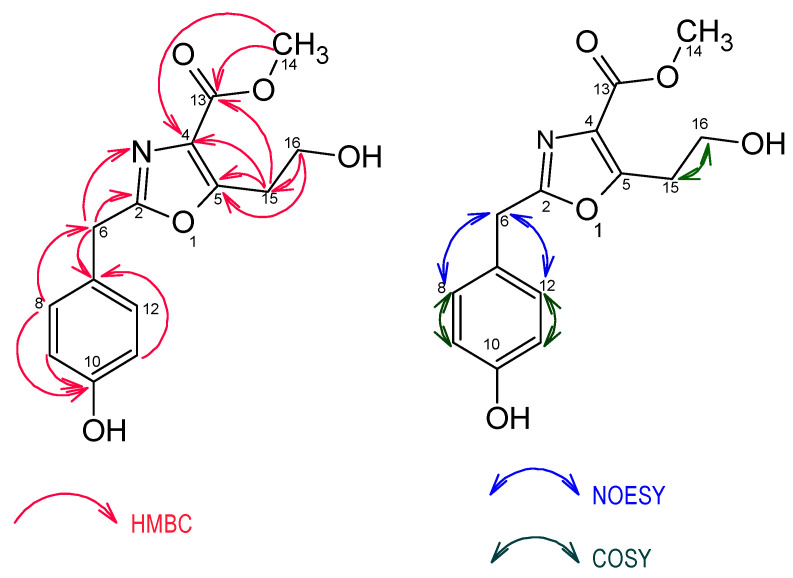
Key HMBC, COSY and NOESY correlations of compound **1**.

The presence of a methyl-2,5-disubstituted oxazole-4-carboxylate moiety was evidenced by resonance of sp^2^ carbon signals at *δ* 164.4 (C-2), *δ* 159.1 (C-5), *δ* 129.0 (C-4), *δ* 163.9 (C-13), and a methoxy carbon at *δ* 52.4 (C-14). This was further confirmed by HMBC correlations between H-15 (*δ* 3.19) and C-5 (*δ* 159.1)/C-4 (*δ* 129.0)/C-13 (*δ* 163.9), H-14 (*δ* 3.87) and C-13 (*δ* 163.9)/C-4(*δ* 129.0), H-6 (*δ* 4.00) and C-2 (*δ* 164.4) as well as the ^15^N-^1^H HMBC correlation of H-6 (*δ* 4.00) to N-3 (*δ* 244.1) (Figure 4). The HMBC correlations of H-6 (*δ* 4.00) to C-2 (*δ* 164.4) and N-3 (*δ* 244.1) revealed the connectivity of the para-hydroxybenzyl moiety to the C-2 carbon of the oxazole moiety confirming unambiguously the structure of compound **1** as methyl 5-(2-hydroxyethyl)-2-(4-hydroxybenzyl)-oxazole-4-carboxylate, named macrooxazole A.

Compound **2** was obtained as a yellow oil from both supernatant and mycelia. Its molecular formula C_14_H_15_NO_6_ (8 degrees of unsaturation) was determined by the [M + H]^+^ ion at *m*/*z* 294.0974, [M + Na]^+^ ion at *m*/*z* 316.0788 and [M + H − H_2_O]^+^ ion at *m*/*z* 276.0865 from the HR-ESIMS data (positive mode). Its NMR spectroscopic data displayed high similarities to those of compound **1**, suggesting that they are close analogues. The only structural difference was the presence of the hydroxyl group at C-15 of compound **2** which was absent in compound **1**. This was confirmed not only by the ^1^H-^1^H COSY coupling of H-15 (*δ* 5.33) to H-16a (*δ* 3.73)/H-16b (*δ* 3.78) but also by the HMBC correlation between H-15 (*δ* 5.33) and C-16 (*δ* 65.1). Interestingly, obtaining an optical rotation value approaching zero identified compound **2** to be a racemic mixture. Consequently, compound **2** was determined as a racemic mixture of methyl 5-(1,2-dihydroxyethyl)-2-(4-hydroxybenzyl)-oxazole-4-carboxylate, named macrooxazole B.

The molecular formula of compound **3** isolated from both supernatant and mycelia as a brown oil was established as C_14_H_13_NO_4_ (9 degrees of unsaturation) from the HR-ESIMS which showed an [M + H]^+^ ion at *m*/*z* 260.0917 and an [M + Na]^+^ ion at *m*/*z* 282.0737. Analysis of 1D and 2D NMR revealed a similar structure to **1** with the C-16 hydroxyl group missing in compound **3**, but a double bond Δ^15−16^ at *δ* 123.2 (C-15) and *δ* 121.0 (C-16) were recorded instead (Table 3). The H-15 (*δ* 7.14) showed COSY correlations to H-16a (*δ* 5.58)/H-16b (*δ* 5.96) and HMBC correlations to C-5 (*δ* 155.6)/C-16 (*δ* 121.0) confirming the structure of the previously unreported metabolite **3** as methyl 2-(4-hydroxybenzyl)-vinyloxazole-4-carboxylate, named macrooxazole C.

Fraction F1 (a mixture of compounds **2** and **4** (ratio 1:2)) was isolated as a yellow oil from both supernatant and mycelial extracts. On the basis of HR-ESIMS and 1D/2D NMR data of this mixture, the structure of compound **4** could be determined independently. HR-ESIMS data revealed the molecular formula of compound **4** as C_14_H_13_NO_5_ (9 degrees of unsaturation) provided by the [M + H]^+^ ion at *m*/*z* 276.0866 and [M + Na]^+^ ion at *m*/*z* 298.0862. Detailed analysis of its 1D and 2D NMR data showed similar features to those of compound **3**, except that the olefinic bond C-15 (*δ* 123.2)/C-16 (*δ* 121.0) was substituted by an epoxide group C-15 (*δ* 44.8)/C-16 (*δ* 48.9). The assumption was evidenced from the established molecular formula and was confirmed not only by the COSY correlation of H-15 (*δ* 4.51) to H-16 (*δ* 3.22), but also by HMBC correlations of H-16 (*δ* 3.22) to C-15 (*δ* 44.8)/C-5 (*δ* 155.1). Therefore, compound **4** was elucidated unambiguously as methyl 2-(4-hydroxybenzyl)-5-(oxiran-2-yl)-oxazole-4-carboxylate, named macrooxazole D. As can be seen in Figure 1, compounds **2** and **4** are both also present in the crude extract, suggesting they are both genuine natural products and that compound **2** does not only arise from macrooxazole D (**4**) as an isolation artefact during preparative HPLC separation. However, the conversion could already have taken place during fermentation of the fungus.

For an unambiguous confirmation of its structure, macrocidin Z (**6**) was synthesized starting by attaching 6-heptenoic acid (**7**) to the Evans auxiliary (*R*)-benzyl-2-oxazolidinone (Scheme 1) [18]. The resulting imide **8** was deprotonated at the α-position with NaHMDS to give an enolate which was quenched with iodomethane. The resulting 9.8:1 mixture of diastereomers was separated by column chromatography to afford the major isomer **9** in 79% yield. It was converted to the carboxylic acid **10** in 96% yield by adding LiOH and H_2_O_2_. The tetramic acid **12** was prepared according to a known protocol [17,19,20] by treatment of commercial Boc-Tyr(Allyl)-OH (**11**) with Meldrum’s acid. Its acylation with carboxylic acid **10** via the two-step Yoshii-Yoda protocol [21,22] initially afforded 4-*O*-acyltetramate **13**, which was rearranged to the 3-acyltetramic acid **14**. A ring-closing metathesis reaction using Grubbs catalyst gave *N*-Boc-protected macrocidin Z **15** with an *E*-selectivity > 99% in 89% yield. Macrocidin Z (**6**) was obtained quantitatively upon removal of the Boc-protection group with TFA in 30% total yield over seven steps.

### 2.2. Biological Activities

The isolated metabolites were evaluated for their antimicrobial activity against various bacteria and fungi. The Minimum Inhibitory Concentration (MIC) values showed that only the new metabolite **3** as well as the known macrocidins **5** and **6** were active, whereas the remaining compounds were inactive against the organisms tested (See Table S1 in the supporting information). Macrocidin A (**5**) showed the strongest activity against *Bacillus subtilis* with an MIC value of 16.7 μg/mL which is the same value as that of oxytetracyclin used as positive control. The same compound **5** demonstrated weak activity against *Mycobacterium smegmatis* with an MIC value of 33.3 μg/mL. Compound **3** exhibited moderate activity against *Mucor hiemalis* with an MIC value of 66.7 μg/mL equal to that of nystatin used as a positive control. The latter also inhibited the growth of *Bacilus subtilis* at 66.7 μg/mL. Against *Micrococcus luteus*, compound **6** exhibited weak activity with an MIC value of 66.7 μg/mL. Furthermore, the ability of some of the isolated compounds to inhibit the proliferation of two mammalian cell lines including HeLa cells KB3.1 and mouse fibroblasts L929 was examined. Only the mixture of compounds **2** and **4** (ratio 1:2) showed weak cytotoxic activity against HeLa cells KB3.1 and mouse fibroblasts L929 with an IC_50_ value of 23 μg/mL for both cell lines, whereas compound **5** and **6** only showed a slight inhibition of HeLa cells KB3.1 proliferation (See Table S2 in the supporting information).

**Figure 5:**
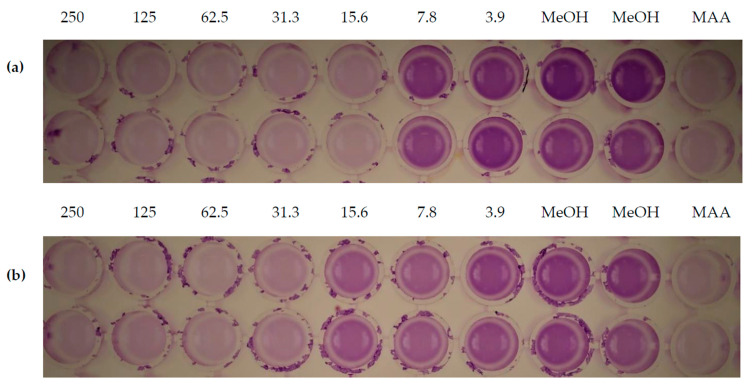
Inhibition of the biofilm formation in *S. aureus* of compounds **5** (**a**) and **6** (**b**). Each column has two replicates in different concentrations of the compounds (250, 125, 62.5, 31.3, 15.6, 7.8 and 3.9 µg/mL) and their respective controls. MAA: microporenic acid A (positive control), MeOH: methanol (negative control).

Moreover, the isolated pure compounds except compound **4** (which was not tested because it was isolated as a mixture) were evaluated for their effectiveness in inhibiting biofilm formation and preformed biofilm of *Staphylococcus aureus* (Table 4). The new compounds **2** and **3** showed moderate-to-weak activity against biofilm formation, with respective inhibition percentages of 65% and 75% at the highest concentration of 250 µg/mL. Compounds **5** and **6** inhibited 61% and 19% of the bacterial biofilm at 15.6 µg/mL, respectively (Figure 5). Interestingly, the test compounds also displayed activity against preformed biofilm of *S. aureus* as represented in Table 4 below. Macrocidins A (**5**) and Z (**6**) exhibited moderate activity against preformed biofilm of *S. aureus* with the highest percentage of inhibition of 75% and 73% at 250 µg/mL, respectively.

Apart from the strong herbicidal activity of macrocidins [15,16], no other activity has been reported for this class of compounds as far as we know. The current paper therefore constitutes the first extensive evaluation of the biological effects for this class of compounds.

## 3. Materials and Methods

### 3.1. General Experimental Procedure

Electrospray mass (ESIMS) spectra were recorded with an UltiMate 3000 Series uHPLC (Thermo Fischer Scientific, Waltman, MA, USA) utilizing a C18 Acquity UPLC BEH column (2.1 × 50 mm, 1.7 µm; Waters, Milford, CT, USA) connected to an amaZon speed ESI-Iontrap-MS (Bruker, Billerica, MA, USA). HPLC parameters were set as follows: solvent A: H_2_O + 0.1% formic acid, solvent B: acetonitrile (ACN) + 0.1% formic acid, gradient: 5% B for 0.5 min increasing to 100% B in 19.5 min, then isocratic condition at 100% B for 5 min, a flow rate of 0.6 mL/min, and diode array detection (DAD) in the range of 190–600 nm.

HR-ESIMS (High-resolution electrospray ionization mass spectrometry) spectra were recorded with an Agilent 1200 Infinity Series HPLC–UV system (Agilent Technologies, Santa Clara, CA, USA) (column 2.1 × 50 mm, 1.7 µm, C18 Acquity UPLC BEH (waters), solvent A: H_2_O + 0.1% formic acid; solvent B: ACN + 0.1% formic acid, gradient: 5% B for 0.5 min increasing to 100% B in 19.5 min and then maintaining 100% B for 5 min, flow rate 0.6 mL/min, UV/Vis detection 200–640 nm) connected to a MaXis ESI-TOF mass spectrometer (Bruker) (scan range 100–2500 *m*/*z*, capillary voltage 4500 V, dry temperature 200 °C). High-resolution mass spectra of synthetic products were obtained with a UPLC/Orbitrap MS system in ESI mode.

Optical rotations were recorded in methanol (Uvasol, Merck, Darmstadt, Germany) by using an Anton Paar MCP-150 polarimeter (Seelze, Germany) at 25 °C for isolated compounds, and by using a PerkinElmer 241 polarimeter for synthetic products dissolved in CHCl_3_, MeCN or methanol. UV/Vis spectra were recorded using methanol (Uvasol, Merck, Darmstadt, Germany) with a Shimadzu UV/Vis 2450 spectrophotometer (Kyoto, Japan). ECD spectra were obtained on a J-815 spectropolarimeter (JASCO, Pfungstadt, Germany). Nuclear magnetic resonance (NMR) spectra were recorded with an Avance III 500 spectrometer (Bruker, ^1^H-NMR: 500 MHz and ^13^C-NMR: 125 MHz). IR spectra were recorded with an FT-IR spectrophotometer equipped with an ATR unit.

For the purification of synthetic products, chromatography silica gel 60 (40–63 μm) or silica gel RP18 (40–63 μm) were used. Analytical thin-layer chromatography (TLC) was carried out using Merck silica gel 60 F_254_ pre-coated aluminum-backed plates.

### 3.2. Fungal Material

The fungal strain *Phoma macrostoma* DAOMC 175,940 was originally isolated from the Canadian thistle *Circium arvense* collected in Quebec, Canada in 1979. It constitutes one of the original producer strains of the macrocidins [15,16,23] and was kindly provided by the CCFC (Canadian Collection of Fungal Cultures, Ottawa, ON, Canada).

### 3.3. Small-Scale Fermentation and Extraction

The fungus was cultivated in Q6 ½ medium (10 g/mL glycerol, 2.5 g/mL d-glucose, 5 g/mL cotton seed flour and pH = 7.2) [24]. A well-grown culture from a yeast-malt (YM) agar plate (10 g/mL malt extract, 4 g/mL yeast extract, 4 g/mL D-glucose, 1.5% agar and pH = 6.3) was cut into small pieces using a cork borer (7mm), and eight pieces were inoculated into 6 × 500 mL Erlenmeyer flasks, each containing 200 mL of the Q6 ½ medium. The culture was incubated at 23 °C on a rotary shaker (140 rpm). The growth of the fungus was monitored by measuring the amount of free glucose using Diastix Harnzuckerstreifen (Bayer). After glucose depletion, small samples were taken to monitor secondary metabolite production over a period of 14 days (searching for the mass spectra and UV/Vis spectra that were reported to be typical for the macrocidins) and a stagnation of the titres of the putative macrocidin derivatives was observed by HPLC–MS between 8 and 14 days.

Then, the fermentation was terminated and the supernatant and mycelia were separated by filtration. The supernatant was extracted with equal amount of ethyl acetate (200 mL) and filtered through anhydrous sodium sulphate. The resulting ethyl acetate extract was evaporated to dryness by means of rotary evaporator. The mycelia was extracted with 200 mL of acetone in an ultrasonic bath at 40 °C for 40 min, filtered and the filtrate evaporated. The remaining water phase was suspended in equal amount of distilled water and subjected to same procedure as the supernatant.

### 3.4. Scale Up of Production in Shake Flask Batches and Extraction

Four well-grown 17-day-old YM agar plates of the mycelial culture were cut into small pieces using a 7 mm cork borer and 8 pieces inoculated in 30 × 500 mL Erlenmeyer flasks containing 200 mL of Q6 ½ medium. The culture was incubated at 23 °C on a rotary shaker (140 rpm) for 13 days. Fermentation was aborted 10 days after the depletion of free glucose.

The mycelia and supernatant from the batch fermentation were separated via filtration. The mycelia was extracted with 4 × 500 mL of acetone in an ultrasonic water bath at 40 °C for 40 min. The extracts were combined and the solvent evaporated by means of a rotary evaporator. The remaining water phase was four times subjected to the same procedure as mycelium in small-scale extraction yielding 949 mg dark brown crude extract. The supernatant (6 L) was extracted with an equal amount of ethyl acetate and filtered through anhydrous sodium sulphate. The resulting ethyl acetate extract was evaporated to dryness by means of rotary evaporator to afford 238 mg of brown crude extract.

### 3.5. Isolation of Compounds ***1**–**6***

The mycelial and the supernatant crude extracts from shake flask batch fermentations (3.4) dissolved in methanol were centrifuged by means of a centrifuge (Hettich Rotofix 32 A, Tuttlingen, Germany) for 10 min at 4000 rpm. The extracts were purified separately using preparative reverse-phase liquid chromatography (PLC 2020; Gilson, Middleton, WI, USA). A VP Nucleodur 100–5 C18ec column (250 × 21 mm, 7 µm: Machery-Nagel, Düren, Germany) was used as the stationary phase. Deionized water (Milli-Q, Millipore, Schwalbach, Germany) with 0.1% formic acid (FA) (solvent A) and acetonitrile (ACN) with 0.1% FA (solvent B) were used as the mobile phase. The elution gradient used was 5–45% solvent B for 20 min, 45–60% B for 15 min, 60–100% B for 10 min and thereafter isocratic condition at 100% solvent B for 5 min. The flow rate was 15 mL/min and the fractions obtained from both the supernatant and mycelial extracts were combined according to UV absorption at 190, 210 and 280 nm as well as concurrent HPLC–MS analyses to yield compound **1** (7.98 mg, *t_R_*: 3.7–3.9 min), **2** (5.20 mg, *t_R_*: 2.3–2.5 min), **3** (8.22 mg, *t_R_*: 6.4–6.6 min), **5** (39.12 mg, *t_R_*: 7.4–7.6 min), **6** (32.10 mg, *t_R:_* 9.3–9.5 min) and fraction F1 (7.20 mg), which was a mixture of compounds **2** (*t_R_*: 2.3–2.5 min) and **4** (*t_R_*: 4.8–5.0 min) with compound **4** as the major component (ratio 1:2).

A total of 5 mg of F1 was further purified by reversed phase HPLC (solvent A (H_2_O + 0.1% FA)/solvent B (ACN + 0.1% FA)), elution gradient 20–50% solvent B for 35 min followed by maintaining isocratic condition at 100% solvent B for 5 min with a preparative HPLC column (VP Nucleodur 100–10 C18ec column (250 × 10 mm, 7 µm: Machery-Nagel, Düren, Germany) as stationary phase) and a flow rate of 8 mL/min, to afford only compound **2**. The absence of the peak of compound **4** in the obtained HPLC chromatogram suggests the instability of compound **4** which easily turns into compound **2**.

### 3.6. Physico-Chemical Characteristics of Compounds ***1**–**6***

*Methyl 5-(2-Hydroxyethyl)-2-(4-hydroxybenzyl)-oxazole-4-carboxylate (Macrooxazole A* (**1***)):* Yellow oil. UV (MeOH, c = 0.025 mg/mL) λ_max_ (log ε) 202 (4.15), 227 (4.14), 277 (3.36) nm. HR-ESIMS *m*/*z* 300.0837 [M + Na] ^+^; *m*/*z* 555.1968 [2M + H]^+^; *m*/*z* 278.1026 [M + H]^+^ (Calcd for C_14_H_16_NO_5_ 278.1023). For NMR data, see Table 2.

*Methyl 5-(1,2-Dihydroxyethyl)-2-(4-hydroxybenzyl)-oxazole-4-carboxylate (Macrooxazole B (***2***))*: Yellow oil. [α]^25^_D_ = 0 ° (c = 0.002, MeOH); UV (MeOH, c = 0.025 mg/mL) λ_max_ (log ε) 202 (4.15), 227 (4.12), 278 (3.21) nm. HR-ESIMS *m*/*z* 316.0788 [M + Na]^+^; *m*/*z* 276.0865 [M + H − H_2_O]^+^; *m*/*z* 294.0974 [M + H]^+^ (Calcd for C_14_H_16_NO_6_ 294.0972). For NMR data, see Table 2.

*Methyl 2-(4-Hydroxybenzyl)-vinyloxazole-4-carboxylate (Macrooxazole C (***3***))*: Brown oil. UV (MeOH, c = 0.025 mg/mL) λ_max_ (log ε) 201 (4.01), 228 (3.96), 272 (3.89) nm. HR-ESIMS *m*/*z* 282.0737 [M + Na]^+^; *m*/*z* 260.0917 [M + H]^+^ (Calcd for C_14_H_14_NO_4_ 260.0917). For NMR data, see Table 3.

*Methyl 2-(4-Hydroxybenzyl)-5-(oxiran-2-yl)-oxazole-4-carboxylate (macrooxazole D (***4***))*: Yellow oil. UV (MeOH, c = 0.025 mg/mL) λ_max_ (log ε) 201 (4.06), 227 (3.96), 274 (3.35) nm. HR-ESIMS *m*/*z* 298.0682 [M + Na]^+^; *m*/*z* 551.1656 [2M + H]^+^; *m*/*z* 276.0866 [M + H]^+^ (Calcd for C_14_H_14_NO_5_ 276.0866). For NMR data, see Table 3.

*Macrocidin A (***5***)*: Beige-yellowish solid. [α]^25^_D_ = +45 ° (c = 0.001, MeOH); UV (MeOH, c = 0.025 mg/mL) λ_max_ (log ε) 201 (4.25), 224 (4.16), 281 (4.14) nm; CD (c = 2.8 × 10^−3^ M, MeOH) λ_max_ (Δε) 218 (+11.77), 234 (−9.41), 274 (+8.91). HR-ESIMS *m*/*z* 715.3223 [2M + H]^+^; *m*/*z* 340.1547 [M + H − H_2_O]^+^ *m*/*z* 380.1465 [M + Na]^+^; *m*/*z* 358.1655 [M + H]^+^ (Calcd for C_20_H_24_NO_5_ 358.1649). ^1^H-NMR (500 MHz, MeOH-*d4*): *δ*_H_ 6.82 (2H, d, *J* = 8.7 Hz, H-3/H-21); *δ*_H_ 7.04 (2H, br d, *J* = 8.7 Hz, H-4/H-22), *δ*_H_ 2.92 (1H, dd, *J* = 14.1, 4.0 Hz, H-6a), *δ*_H_ 3.12 (1H, dd, *J* = 14.1, 3.4 Hz, H-6b), *δ*_H_ 4.11 (1H, t, *J* = 3.6 Hz, H-7), *δ*_H_ 3.59 (1H, m, H-12), *δ*_H_ 1.46 (1H, ddd, *J* = 13.1, 11.6, 4.1 Hz, H-13a), *δ*_H_ 1.36 (1H, tt, *J* = 13.1, 4.1 Hz, H-13b), *δ*_H_ 0.46 (1H, qt, *J* = 12.9, 4.7 Hz, H-14a), *δ*_H_ 1.15 (1H, qt, *J* = 13.1, 4.1 Hz, H-14b), *δ*_H_ 1.9 (1H, tt, *J* = 12.8, 3.5 Hz, H-15a), *δ*_H_ 0.76 (1H, tdd, *J* = 12.9, 9.8, 5.0 Hz, H-15b), *δ*_H_ 3.03 (1H, ddd, *J* = 9.9, 3.4, 2.4 Hz, H-16), *δ*_H_ 2.58 (1H, dt, *J* = 8.5, 2.0 Hz, H-17), *δ*_H_ 4.40 (1H, dd, *J* = 12.7, 1.8 Hz, H-18a), *δ*_H_ 3.95 (1H, dd, *J* = 12.7, 8.7 Hz, H-18b), *δ*_H_ 1.08 (3H, d, *J* = 6.9 Hz, H-20). (3H, d, *J* = 6.9 Hz, H-20).

*Macrocidin Z (***6***)*: Yellow oil. [α]^25^_D_ = +123.5 ° (c = 0.00942, MeOH); UV (MeOH, c = 0.025 mg/mL) λ_max_ (log ε) 201 (4.11), 226 (3.92), 281 (3.90) nm; CD (c = 2.9 × 10^−3^ M, MeOH) λ_max_ (Δε) 219 (+7.67), 237 (−4.87), 274 (+9.36). HR-ESIMS *m*/*z* 683.3324 [2M + H]^+^; *m*/*z* 342.1701 [M + H]^+^ (Calcd for C_20_H_24_NO_4_ 342.1700). For NMR data, see Table 2.

### 3.7. Synthesis of Macrocidin Z *(**6**)*

*(R)-4-Benzyl-3-(hept-6-enoyl)oxazolidin-2-one* (**8**) [25]: 6-Heptenoic acid (**7**; 100 μL, 738 μmol) in dry CH_2_Cl_2_ (1 mL) at 0 °C was treated with DMAP (dimethylaminopyridine; 8.20 mg, 738 μmol), (R)-benzyl-2-oxazolidinone (119 mg, 671 μmol) and DCC (dicyclohexylcarbodiimide;152 mg, 738 μmol). The mixture was stirred for 23 h at room temperature. The white precipitate was filtered off and washed with CH_2_Cl_2_. The filtrate was washed with aqueous sat. NaHCO_3_ solution and the aqueous phase was extracted with CH_2_Cl_2_ (2 × 20 mL). The combined organic phases were dried over Na_2_SO_4_. After removing the solvent under reduced pressure, the crude product was purified by silica gel column chromatography on silica gel 60 using a mobile phase of 12.5% ethyl acetate (EtOAc in hexane, to give imide **8** (166 mg, 86%) as a colorless oil; R_f_ = 0.68 (25% EtOAc in hexanes); ^1^H-NMR (500 MHz, CDCl_3_) *δ* 1.49 (quin, *J* = 7.6 Hz, 2H), 1.74 (m, 2H), 2.11 (m, 2H), 2.77 (dd, *J* = 13.3, 9.6 Hz, 1H), 2.95 (m, 2H), 3.30 (dd, *J* = 13.3, 3.3 Hz, 1H), 4.19 (m, 2H), 4.67 (m, 1H), 4.97 (m, 1H), 5.03 (dq, *J* = 17.2, 1.5 Hz, 1H), 5.82 (m, 1H), 7.19–7.36 (m, 5H); ^13^C-NMR (125 MHz, CDCl_3_) *δ* 23.8, 28.4, 33.6, 35.5, 38.1, 55.3, 66.3, 114.9, 127.5, 129.1, 129.6, 135.4, 138.6, 153.6, 173.4.

*(R,R)-4-Benzyl-3-(2-methylhept-6-enoyl)oxazolidin-2-one* (**9**): A solution of imide **8** (1.32 g, 4.60 mmol) in dry THF (11 mL) at –78 °C was treated with 1M NaHMDS in THF (5.29 mL, 5.29 mmol), stirred for 30 min, and then quenched with iodomethane (1.50 mL, 24.0 mmol). The resulting mixture was stirred for 4.5 h, diluted with H_2_O (100 mL), and the aqueous phase was extracted with Et_2_O (3 × 75 mL). The combined organic phases were dried over Na_2_SO_4_ and concentrated under reduced pressure to give a crude product which was purified by column chromatography (silica gel 60, 10% EtOAc in hexanes) to leave **9** (1.10 g, 79%) as a colorless oil; R_f_ = 0.72 (25% EtOAc in hexanes); [α]^24^_D_–61.6 (c 1.46, CHCl_3_); IR ν_max_ 3079 (w), 3024 (w), 2974 (w), 2932 (m), 2857 (w), 1779 (s), 1697 (s), 1455 (w), 1385 (m), 1350 (m), 1240 (m), 1211 (m), 1196 (m), 1101 (w), 913 (w), 703 (m) cm^−1^; ^1^H-NMR (500 MHz, CDCl_3_) *δ* 1.23 (d, *J* = 6.88 Hz, 3H), 1.43 (m, 3H), 1.76 (m, 1H), 2.01 (m, 2H), 2.77 (dd, *J* = 13.2, 9.5 Hz, 1H), 3.27 (dd, *J* = 13.3, 3.1 Hz, 1H), 3.72 (m, 1H), 4.19 (m, 2H), 4.67 (m, 1H), 4.95 (m, 1H), 5.01 (dq, *J* = 17.2, 1.7 Hz, 1H), 5.79 (m, 1H), 7.19–7.36 (m, 5H); ^13^C-NMR (125 MHz, CDCl_3_) *δ* 17.5, 26.6, 33.0, 33.8, 37.7, 38.0, 55.5, 66.1, 114.8, 127.5, 129.0, 129.6, 135.4, 138.6, 153.2, 177.3; HRMS (ESI): *m*/*z* [C_18_H_23_NO_3_ + H^+^]: calcd 302.17507, found 302.17438.

*(R)-2-Methylhept-6-enoic acid* (**10**): A solution of imide **9** (1.20 g, 3.98 mmol) in THF (36 mL) and H_2_O (16 mL) was treated with LiOH H_2_O (334 mg, 7.96 mmol) and H_2_O_2_ (30 wt%, 2.03 mL, 19.9 mmol) at 0 °C. After stirring at room temperature for 1 d, sat. aqueous NaHCO_3_ solution (50 mL) was added, the aqueous layer was extracted with CH_2_Cl_2_ (50 mL), acidified with 1M HCl to pH = 2, and extracted with Et_2_O (3 × 40 mL). The combined organic phases were dried over Na_2_SO_4,_ and concentrated under reduced pressure to give **4** (546 mg, 96%) as a colorless liquid; R_f_ = 0.37 (25% EtOAc in hexanes); [α]^24^_D_ –17.4 (c 0.69, CHCl_3_); IR ν_max_ 2978 (m), 2936 (m), 2867 (m), 1706 (s), 1466 (w), 1414 (w), 1233 (m), 992 (w), 911 (m) cm^−1^; ^1^H-NMR (500 MHz, CDCl_3_) *δ* 1.19 (d, *J* = 5.9 Hz, 3H), 1.44 (m, 3H), 1.69 (m, 1H), 2.06 (m, 2H), 2.47 (m, 1H), 4.95 (d, *J* = 5.0 Hz, 1H), 5.01 (d, *J* = 5.0 Hz, 1H), 5.79 (m, 1H), 11.5 (br, 1H, OH); ^13^C-NMR (125 MHz, CDCl_3_) *δ* 17.0, 26.5, 33.1, 33.7, 39.3, 114.9, 138.5, 183.1; HRMS (ESI): *m*/*z* [C_8_H_14_O_2_ + H^+^]: calcd 143.10666, found 143.10656.

*(S)-tert-Butyl-2-(4-(allyloxy)benzyl)-3,5-dioxopyrrolidin-1-carboxylate* (**12**) [17]: A solution of Boc-Tyr(Allyl)-OH (**11**; 1.28 g, 3.99 mmol) in dry CH_2_Cl_2_ (13 mL) was treated with Meldrum´s acid (633 mg, 4.39 mmol), DMAP (683 mg, 5.59 mmol) and 1-ethyl-3-(3-dimethylaminopropyl)carbodiimide (EDC)-∙HCl (918 mg, 4.79 mmol) at room temperature. The mixture was stirred for 2 h, concentrated under reduced pressure, and the resulting crude product was diluted with EtOAc (100 mL) and washed with 0.5 M H_2_SO_4_ (3 × 40 mL). The combined aqueous phases were extracted with EtOAc (2 × 50 mL). The combined organic phases were washed with H_2_O (100 mL), dried over Na_2_SO_4_ and refluxed until gas formation ceased. The solvent was removed under reduced pressure to give **12** (1.32 g, 96%) as a yellow foam; *R_f_* = 0.63 (10% MeOH in CH_2_Cl_2_); IR ν_max_ 2976 (w), 1755 (m), 1711 (m), 1610 (m), 1510 (m), 1364 (m), 1298 (m), 1240 (s), 1149 (s), 1077 (m), 1021 (m), 997 (m), 925 (w), 829 (m), 813 (m), 788 (w), 772 (w), 756 (w), 656 (w) cm^−1^; ^1^H-NMR (500 MHz, CD_3_OD) *δ* 1.61 (s, 9H), 3.10 (dd, *J* = 14.2, 2.6 Hz, 1H), 3.41 (dd, *J* = 14.2, 5.3 Hz, 1H), 4.47 (dt, *J* = 5.2, 1.6 Hz, 2H), 4.64 (dd, *J* = 5.3, 2.6 Hz, 1H), 5.22 (dq, *J* = 10.5, 1.6 Hz, 1H), 5.37 (dq, *J* = 17.5, 1.6 Hz, 1H), 6.03 (ddt, *J* = 17.5, 10.5, 5.2 Hz, 1H), 6.78 (m, 2H), 6.97 (m, 2H); ^13^C-NMR (125 MHz, CD_3_OD) *δ* 28.5, 35.0, 62.2, 69.7, 83.9, 115.3, 117.3, 127.5, 131.9, 135.0, 150.9, 159.3, 173.4, 178.2.

*(2S)-tert-Butyl-2-(4-(allyloxy)benzyl)-3-(((R)-2-methylhept-6-enoyl)oxy)-5-oxopyrrolidine-1-carboxylate* (**13**): A stirred solution of carboxylic acid **10** (397 mg, 2.79 mmol) in dry CH_2_Cl_2_ (14 mL) at 0 °C was treated with EDC HCl (642 mg, 3.35 mmol) and DMAP (68.2 mg, 558 μmol), allowed to warm to room temperature and stirred for 30 min. The resulting mixture was treated with tetramic acid **12** (1.16 g, 3.35 mmol), stirred for a further 4 h, and then diluted with 0.5M H_2_SO_4_ (200 mL). The aqueous phase was extracted with EtOAc (3 × 50 mL) and the combined organic phases were washed with brine and dried over Na_2_SO_4_. After the removal of the solvent under reduced pressure, the crude product was purified by column chromatography (silica gel 60, 10% EtOAc in hexanes → 15% EtOAc in hexanes → 20% EtOAc in hexanes → 25% EtOAc in hexanes → 100% EtOAc) to afford **13** (967mg, 73%) as a yellowish oil; *R_f_* = 0.93 (40% EtOAc in hexanes); [α]^24^_D_ 90.3 (*c* 1.15, CHCl_3_); IR ν_max_ 2971 (w), 2938 (w), 1781 (m), 1742 (s), 1631 (m), 1512 (m), 1368 (m), 1322 (m), 1229 (m), 1217 (m), 1175 (m), 1063 (m), 912 (w), 847 (w) cm^−1^; ^1^H-NMR (500 MHz, CDCl_3_) *δ* 1.25 (t, *J* = 6.9 Hz, 3H), 1.43–1.57 (m, 3H), 1.60 (s, 9H), 1.76 (m, 1H), 2.10 (m, 2H), 2.62 (m, 1H), 3.11 (dd, *J* = 14.2, 2.6 Hz, 1H), 3.36 (dd, *J* = 14.2, 6.0 Hz, 1H), 4.48 (m, 2H), 4.77 (m, 1H), 5.00 (dd, *J* = 10.4, 1H), 5.04 (d, *J* = 17.2, 1H), 5.27 (d, *J* = 10.4, 1H), 5.39 (d, *J* = 17.2, 1H), 5.80 (m, 1H), 6.03 (m, 1H), 6.77 (m, 2H), 6.90 (m, 2H); ^13^C-NMR (125 MHz, CDCl_3_) *δ* 16.8, 16.4, 28.3, 32.8, 33.6, 34.8, 40.0, 60.7, 68.9, 83.2, 108.3, 114.8, 115.4, 117.8, 126.0, 130.5, 133.3, 138.0, 149.4, 157.9, 165.1, 168.2, 171.9 [some peaks are doubled due to **13** partially rearranging to **14** on silica gel]; HRMS (ESI): *m*/*z* [C_27_H_35_NO_6_ + Na^+^]: calcd 492.23566, found 492.23499.

*(S,Z)-tert-Butyl-2-(4-(allyloxy)benzyl)-4-(1-hydroxy-(2R)-methylhept-6-en-1-ylidene)-3,5-dioxopyrrolidine-1-carboxylate* (**14**): A solution of **13** (967 mg, 2.05 mmol) in dry CH_2_Cl_2_ (20 mL) was treated with NEt_3_ (344 μL, 2.46 mmol) and DMAP (125 mg, 1.03 mmol) and stirred at room temperature for 7 h. DMAP (62.6 mg, 0.51 mmol) was added and the stirring continued for 16 h. The reaction was quenched with sat. aqueous NaHCO_3_ solution (100 mL), the aqueous phase was extracted with EtOAc (3 × 50 mL), and the combined organic phases were washed with brine and dried over Na_2_SO_4_. The solvent was removed under reduced pressure and the remainder was purified by column chromatography on reversed phase silica gel (RP18, 40% MeCN in H_2_O + 0.01% HCOOH → 60% MeCN in H_2_O + 0.01% HCOOH → 80% MeCN in H_2_O + 0.01% HCOOH → 100% MeCN + 0.01% HCOOH) to yield **14** (684 mg, 71%) as a yellow oil; *R_f_* = 0.94 (10% MeOH in CH_2_Cl_2_); [α]^24^_D_–26.9 (*c* 2.12, MeOH); IR ν_max_ 3463 (w), 3016 (m), 2970 (m), 2945 (m), 1738 (s), 1599 (w), 1510 (w), 1366 (s), 1229 (s), 1217 (s), 1206 (s), 1156 (w), 907 (m), 787 (w) cm^−1^; ^1^H-NMR (500 MHz, CD_3_OD) *δ* 1.01 (m, 3H), 1.23 (m, 1H), 1.28–1.42 (m, 2H), 1.63 (m, 1H), 1.63 (s, 9H), 2.04 (q, *J* = 6.7 Hz, 2H), 3.19 (dd, *J* = 14.2, 2.7 Hz, 1H), 3.38 (dd, *J* = 14.2, 5.3 Hz, 1H), 3.56 (m, 1H), 4.47 (m, 2H), 4.58 (br, 1H), 4.95 (m, 1H), 5.01 (dq, *J* = 17.0, 1.7 Hz, 1H), 5.21 (dq, *J* = 10.6, 1.6 Hz, 1H), 5.35 (dq, *J* = 17.3, 1.6 Hz, 1H), 5.78 (ddt, *J* = 17.0, 10.3, 6.7 Hz, 1H), 6.03 (ddt, *J* = 17.3, 10.6, 5.2 Hz, 1H), 6.76 (m, 2H), 6.90 (m, 2H); ^13^C-NMR (125 MHz, CD_3_OD) *δ* 17.3, 27.4, 28.4, 33.7, 34.7, 35.8, 37.9, 69.7, 84.9, 115.3, 115.5, 117.4, 127.4, 131.9, 134.8, 139.5, 159.3, 195.1; ^1^H-NMR (500 MHz, CDCl_3_) *δ* 0.91/1.13 (d, *J* = 6.9 Hz, 3H), 1.15–1.44 (m, 3H), 1.59 (m, 1H), 1.62 (s, 9H), 2.02 (m, 2H), 3.20/3.26 (dd, *J* = 13.8, 2.0 Hz, 1H), 3.34/3.40 (dd, *J* = 13.8, 5.6 Hz, 1H), 3.48/3.65 (m, 1H), 4.38/4.63 (m, 1H), 4.44 (m, 2H), 4.88–5.06 (m, 2H), 5.22–5.41 (m, 2H), 5.75 (m, 1H), 6.01 (m, 1H), 6.73 (m, 2H), 6.91 (m, 2H); ^13^C-NMR (125 MHz, CDCl_3_) *δ* 17.1/17.4, 26.5/26.6, 28.2/28.3, 32.2/33.3, 33.8, 34.9/35.0, 36.3/37.6, 61.8/65.7, 68.7/68.8, 83.4/84.1, 102.1/105.1, 114.7, 114.8/115.1, 117.7/117.8, 126.0/126.4, 130.7/130.9, 133.3, 138.4/138.5, 149.0/150.0, 157.8/157.9, 164.2/173.8, 192.3, 195.6, 197.3, 200.8 [not all quaternary C-atoms are visible in the JMOD in CD_3_OD; some of them can be seen via HSQC or HMBC correlations. Some peaks in the ^1^H-NMR and JMOD in CDCl_3_ are doubled because of tautomers]; HRMS (ESI): *m*/*z* [C_27_H_35_NO_6_ + Na^+^]: calcd 492.23566, found 492.23450.

*N-Boc-Macrocidin Z* (**15**): A solution of diene **14** (634 mg, 1.35 mmol) in degassed CH_2_Cl_2_ (270 mL) was treated with 2nd generation Grubbs catalyst (57.3 mg, 67.5 μmol) and heated at reflux for 16 h. The solvent was removed under reduced pressure and the remainder was purified by column chromatography on reversed phase silica gel (RP18, 40% MeCN in H_2_O + 0.01% HCOOH → 60% MeCN in H_2_O + 0.01% HCOOH → 80% MeCN in H_2_O + 0.01% HCOOH) to yield **15** (529 mg, 89%) as a brownish foam; *R_f_* = 0.94 (10% MeOH in CH_2_Cl_2_); [α]^24^_D_ 155.8 (c 0.62, MeOH); IR ν_max_ 3456 (m), 3016 (m), 2970 (s), 2944 (m), 2136 (w), 1740 (s), 1728 (s), 1600 (m), 1436 (m), 1366 (s), 1354 (s), 1299 (m), 1228 (s), 1216 (s), 1296 (m), 1091 (w), 974 (w), 908 (m), 730 (w), 786 (w) cm^−1^;. ^1^H-NMR (500 MHz, CD_3_OD) *δ* 0.91 (m, 1H), 1.08 (d, *J* = 6.7 Hz, 3H), 1.14 (m, 2H), 1.40 (m, 1H), 1.63 (s, 9H), 1.82 (m, 1H), 2.11 (m, 1H), 3.07 (dd, *J* = 14.3, 3.5 Hz, 1H), 3.36 (dd, *J* = 14.3, 3.1 Hz, 1H), 3.47 (m, 1H), 4.46 (m, 1H), 4.54 (m, 1H), 4.65 (dd, *J* = 13.4, 9.1 Hz, 1H), 5.27 (m, 1H), 5.69 (m, 1H), 6.60–6.97 (m, 4H); ^13^C-NMR (125 MHz, CD_3_OD) *δ* 15.0, 27.7, 28.3, 33.2, 34.8, 35.6, 37.1, 66.8, 67.9, 85.3, 102.9, 115.6, 119.0, 126.8, 130.8, 132.6, 138.8, 157.6, 174.9, 192.4, 193.8; ^1^H-NMR (500 MHz, CDCl_3_) *δ* 0.89 (m, 1H), 1.07 (d, *J* = 6.7 Hz, 3H), 1.13 (m, 2H), 1.32 (m, 1H), 1.63 (s, 9H), 1.82 (m, 1H), 2.05 (m, 1H), 3.12 (dd, *J* = 14.3, 3.5 Hz, 1H), 3.37 (dd, *J* = 14.3, 3.1 Hz, 1H), 3.50 (m, 1H), 4.41 (t, *J* = 3.4 Hz, 1H), 4.59 (d, *J* = 6.2 Hz, 2H), 5.26 (dt, *J* = 15.5, 6.2 Hz, 1H), 5.58 (m, 1H), 6.59–7.01 (m, 4H); ^13^C-NMR (125 MHz, CDCl_3_) *δ* 13.77/14.97, 26.3/26.6, 28.3/28.4, 32.2/33.3, 33.9, 34.2/34.6/34.9, 35.6/37.1, 61.7/65.7, 67.3/67.7, 84.3, 102.6, 114.5, 118.5, 125.3/125.5, 125.7, 130.0, 131.6, 135.0/137.6, 148.9, 156.0, 174.1, 191.9, 195.4 [not all quaternary C-atoms are visible in the JMOD in CD_3_OD, some of them can be seen via HSQC or HMBC correlations. Some peaks in the ^1^H-NMR and JMOD in CDCl_3_ are doubled because of tautomers]; HRMS (ESI): *m*/*z* [C_25_H_31_NO_6_ + Na^+^]: calcd 464.20436, found 464.20413.

*Macrocidin Z* (**6**): A solution of Boc-protected macrocidin Z (**15**) (238 mg, 539 μmol) in dry CH_2_Cl_2_ (10 mL) was treated with TFA (1.00 mL), stirred for 15 min, diluted with toluene (100 mL) and finally concentrated under reduced pressure. This procedure was repeated once to afford macrocidin Z (**6**) (183 mg, quant.) as a pale yellow foam; *R_f_* = 0.60 (10% MeOH in CH_2_Cl_2_); [α]^25^_D_ +126.1 (c 0.85, MeOH); IR ν_max_ 2934 (m), 2864 (w), 1696 (m), 1656 (s), 1607 (s), 1508 (s), 1448 (m), 1338 (m), 1250 (m), 1217 (m), 1177 (w), 976 (m), 843 (w) cm^−1^; ^1^H-NMR (500 MHz, CD_3_OD) *δ* 0.83 (m, 1H, C*H*^a^HCC=C), 1.05 (d, *J* = 6.8 Hz, 3H, CH_3_), 1.13 (m, 2H, C*H*_2_CMe), 1.32 (m, 1H, CH*H*^b^CC=C), 1.79 (m, 1H, CC*H*^a^HC=C), 2.06 (m, 1H, CCH*H*^b^C=C), 2.89 (dd, *J* = 14.1, 3.1 Hz, 1H, PhC*H*^a^H), 3.07 (dd, *J* = 14.1, 3.9 Hz, 1H, PhCH*H*^b^), 3.39 (sex, *J* = 6.8 Hz, 1H, C*H*Me), 4.10 (m, 1H, CHN), 4.53 (m, 1H, C*H*^a^HO), 4.64 (dd, *J* = 13.4, 9.5 Hz, 1H, CH*H*^b^O), 5.26 (m, 1H, OHC=C), 5.68 (m, 1H, C=CHC), 6.71 (m, 2H, H^ortho^), 6.97 (m, 2H, H^meta^); ^13^C-NMR (125 MHz, CD_3_OD) *δ* 15.2 (Me), 28.1 (*C*CC=C), 33.4 (*C*C=C), 35.1 (*C*CMe), 36.5 (PhC), 36.8 (CMe), 63.8 (HCN), 67.9 (OCH_2_), 102.1 (NC*C*=C), 115.7, 118.7 (C^ortho^), 126.7 (OCH_2_*C*), 127.3 (C^para^), 131.4, 132.5 (C^meta^), 139.0 (OCH_2_C=*C*), 157.3 (C^ipso^), 175.5 (NCO), 191.8 (COH), 197.1 (C*C*(O)C); ^1^H-NMR (500 MHz, CDCl_3_) *δ* 0.86 (m, 1H), 1.07 (d, *J* = 6.9 Hz, 3H), 1.09–1.35 (m, 3H), 1.80 (m, 1H), 2.03 (m, 1H), 2.86 (dd, *J* = 14.4, 2.9 Hz, 1H), 3.20 (dd, *J* = 14.4, 3.7 Hz, 1H), 3.44 (sex, *J* = 6.9 Hz, 1H), 4.14 (t, *J* = 3.7 Hz, 1H), 4.60 (m, 2H), 5.27 (m, 1H), 5.59 (m, 1H), 6.17 (s, 1H), 6.71 (br, 2H), 7.01 (m, 2H); ^13^C-NMR (125 MHz, CDCl_3_) *δ* 15.1, 27.0, 32.4, 34.2, 35.5, 36.1, 62.3, 67.4, 100.9, 114.8, 118.1, 125.2, 125.7, 130.3, 131.7, 137.9, 156.0, 175.8, 192.2, 194.3 [not all quaternary C-atoms are visible in the JMOD in CD_3_OD, some of them can be seen via HSQC or HMBC correlations]; HRMS (ESI): *m*/*z* [C_20_H_23_NO_4_ + H^+^]: calcd 342.16998, found 342.16907.

### 3.8. Antimicrobial Assay

Minimum Inhibitory Concentrations (MIC) of compounds **1**–**6** were determined in serial dilution assays as described previously [26,27] using different test microorganisms including *Pichia anomala, Schizosaccharomyces pombe, Mucor hiemalis, Candida albicans,* and *Rhodotulas glutinis* for fungal microorganisms; *Micrococcus luteus, Bacillus subtilis, Staphyloccocus aureus* and *Mycobacterium smegmatis* for Gram-positive bacteria; *Chromobacterium violaceum, Escherichia coli* and *Pseudomonas aeruginosa* for Gram-negative bacteria. A detailed protocol can be found in the Supporting Information.

### 3.9. Cytotoxicity Assay

The in vitro cytotoxicity (IC_50_) of compounds **1**–**6** was determined against mammalian cell lines including mouse fibroblast L929 and Hela (KB3.1) cells according to our previously reported procedures [26,27]. A detailed protocol is given in the Supporting Information.

### 3.10. Biofilm Inhibition Assay

*Staphylococcus aureus* DSM 1104 from −20 °C stock was incubated in 20 mL CASO (casein-peptone soymeal-peptone) medium at 37 °C on a rotary shaker (100 rpm) overnight. The OD_600_ of the culture solution was measured and adjusted to match the turbidity of a 0.001 McFarland standard. A total of 150 µL of CASO with 4% glucose broth was added together with the serial diluted compounds (250–3.9 µg/mL) and incubated in 96 well microtiter plates (TPP tissue culture ref.no 92196) for 18 h at 37 °C. The biofilm inhibition activity of the test compounds was evaluated by using 0.1% crystal violet staining (Thermo Fisher, Waltham, USA) following previously established protocols [28,29]. In brief, the supernatant was discarded, the biofilm stained at room temperature with 0.1% crystal violet for 15 min and washed three times by using PBS (phosphate-buffered saline) buffer, the dye in the biofilm was extracted with diluted acetic acid (30%), and the absorbance was finally quantified in a plate reader (Synergy 2, BioTek, Santa Clara, USA) at 550 nm. Methanol (5%) was used as a negative control and microporenic acid A [28] (250–7.9 µg/mL) was used as a positive control. Standard deviations (SD) of three repeats with duplicates each were 15% or less. SD values are shown in Table S5 in the Supporting Information.

### 3.11. Dispersion of Preformed Biofilm

A cell suspension of *Staphylococcus aureus* strain DSM 1104 was adjusted to match the turbidity of a 0.001 McFarland standard and incubated in 96-well tissue microtiter plates for 18 h in CASO with 4% glucose broth. The supernatant was removed from the wells and washed with 150 µL PBS buffer; then, 150 µL of the fresh media (CASO with 4% glucose broth) was added together with the serially diluted compounds (250–3.9 µg/mL) into the wells. The plates were further incubated for 24 h at 37 °C. Staining of the preformed biofilm and controls were described as for the biofilm inhibition [28,29]. All experiments were made in triplicates with two repetitions.

## 4. Conclusions

During the course of our search for new biologically active secondary metabolites, four previously undescribed oxazole carboxylic acid derivatives were isolated from the plant pathogenic fungus *Phoma macrostoma*. As far as we know, these metabolites constitute the first series of oxazole derivatives isolated from this genus. Investigation of the antimicrobial activity of the new isolates revealed that only compound **3** displayed moderate activity against *Bacillus subtilis* and *Mucor hiemalis*. Although none of the isolates displayed any antibacterial activity against *S. aureus*, compounds **2** and **3** showed moderate to weak inhibition of biofilm formation and preformed biofilm of the bacteria. Moreover, two known tetramic acids macrocidins, A and Z, were also characterized. The so far unclear structure of macrocidin Z was confirmed in this study by its first total synthesis. Even though the macrocidins are well known for displaying a strong herbicidal activity, their biological effects have also been extensively evaluated in the present work and they turned out to possess an interesting antibiofilm effect against *S. aureus*. Thanks to their ability to inhibit biofilm formation, these are likely to be considered as promising candidates for the development of lead molecules that could function as adjunctive agents in combination therapy with antibiotics.

## Figures and Tables

**Table 1:** ^13^C and ^1^H-NMR spectroscopic data (^1^H 500 MH_Z_, ^13^C 125 MH_Z_ in Methanol-*d*_4_, *δ* in ppm) for isolated and synthetic compound **6**.

Position	6		Synthetic 6	
	*δ*_C_, Type	*δ*_H_ (*J* in Hz)	*δ*_C_, Type	*δ*_H_ (*J* in Hz)
2	157.4, C		157.3, C	
3/21	115.9, CH	6.69, m	115.7, CH	6.71, m
4/22	132.6, CH	6.98, m	132.5, CH	6.97, m
5	127.6, C		127.3, C	
6	36.6, CH_2_	3.07, dd (14.1, 3.9)	36.5, CH_2_	3.07, dd (14.1, 3.9)
		2.90, dd (14.1, 3.3)		2.89, dd (14.1, 3.1)
7	63.8, CH	4.10, t (3.6)	63.8, CH	4.10, m
9	177.3, C		175.5, C	
10	102.3, C		102.1, C	
11	194.0, C		191.8, C	
12	37.2, CH	3.40, sxt (6.8)	36.8, C	3.39, sxt (6.8)
13	35.3, CH_2_	1.16, tdd (12.9, 6.4, 4.3)	35.1, CH_2_	1.13, m
		1.09, m		
14	28.3, CH_2_	0.83, tddd (12.9, 8.5, 6.5, 4.4)	28.1, CH_2_	0.83, m
		1.32, m		1.32, m
15	33.6, CH_2_	2.06, dq (12.8, 6.2)	33.4, CH_2_	2.06, m
		1.79, m		1.79, m
16	139.1, CH	5.67, ddd (15.5, 8.8, 5.9)	139.0, CH	5.68, m
17	126.6, CH	5.26, ddd (15.5, 8.9, 3.8)	126.7, CH	5.26, m
18	68.1, CH_2_	4.64, dd (13.4, 8.9)	67.9, CH_2_	4.64, dd (13.4, 9.5)
		4.53, dd (13.4, 3.8)		4.53, m
19	197.3, C		197.1, C	
20	15.4, CH_3_	1.05, d (6.8)	15.2, CH_3_	

**Table 2:** ^13^C and ^1^H-NMR spectroscopic data (^1^H 500 MH_Z_, ^13^C 125 MH_Z_ in Methanol-*d*_4_, *δ* in ppm) for compounds **1** and **2**.

Position	1		2	
	*δ*_C_, Type	*δ*_H_ (*J* in Hz)	*δ*_C_, Type	*δ*_H_ (*J* in Hz)
2	164.4, C	-	165.1, C	-
4	129.0, C	-	129.6, C	-
5	159.1, C	-	159.3, C	-
6	34.3, CH_2_	4.00, s	34.3, CH_2_	4.04, s
7	127.0, C	-	126.9, C	-
8	131.0, CH	7.10, d (9.0)	131.0, CH	7.12, d (9.0)
9	116.7, CH	6.73, d (9.0)	116.7, CH	6.73, d (9.0)
10	157.9, C	-	158.0, C	-
11	116.7, CH	6.73, d (9.0)	116.7, CH	6.73, d (9.0)
12	131.0, CH	7.10, d (9.0)	131.0, CH	7.12, d (9.0)
13	163.9, C	-	163.6, C	-
14	52.4, CH_3_	3.87, s	52.6, CH_3_	3.88, s
15	30.8, CH_2_	3.19, t (6.5)	67.3, CH	5.33, t (6.5)
16	60.6, CH_2_	3.80, t (6.5)	65.1, CH_2_	3.73, dd (11, 6.5) 3.78, dd (11, 6.5)

**Table 3:** ^13^C and ^1^H-NMR spectroscopic data (^1^H 500 MH_Z_, ^13^C 125 MH_Z_ in Methanol-*d*_4_, *δ* in ppm) for compounds **3** and **4**.

Position	3		4	
	*δ*_C_, Type	*δ*_H_ (*J* in Hz)	*δ*_C_, Type	*δ*_H_ (*J* in Hz)
2	164.8, C	-	165.3, C	-
4	128.1, C	-	133.0, C	-
5	155.6, C	-	155.1, C	-
6	34.3, CH_2_	4.04, s	34.2, CH_2_	4.00, s
7	126.8, C	-	126.6, C	
8	131.0, CH	7.12, d (9.0)	131.0, CH	7.08, d (9.0)
9	116.8, CH	6.75, d (9.0)	116.7, CH	6.73, d (9.0)
10	158.0, C	-	158.0, C	-
11	116.8, CH	6.75, d (9.0)	116.7, CH	6.73, d (9.0)
12	131.0, CH	7.12, d (9.0)	131.0, CH	7.08, d (9.0)
13	163.4, C	-	163.2, C	-
14	52.6, CH_3_	3.89, s	52.8, CH_3_	3.92, s
15	123.2, CH	7.14, dd (17.5, 11.5)	44.8, CH	4.51, t (3.5)
16	121.0, CH_2_	5.58, dd (11.5, 1.1) 5.96, dd (17.5, 1.1)	48.9 *, CH_2_	3.22, dd (3.5, 1.6)

* Overlapping with the solvent peak.

**Table 4:** Inhibition of biofilm and destruction of preformed biofilm in *Staphylococcus aureus* by compounds **1**–**3**, **5**, **6**.

Compounds	Inhibition of Biofilm Formation (%)	Destruction of Preformed Biofilm (%)
**1**	-	-
**2**	65 (250 µg/mL) 43 (125 µg/mL)	36 (250 µg/mL) 31 (125 µg/mL)
**3**	75 (250 µg/mL) 59 (125 µg/mL)	57 (250 µg/mL) 48 (125 µg/mL)
**4**	n.t	n.t
**5**	79 (250 µg/mL) 77 (62.5 µg/mL) 61 (15.6 µg/mL)	75 (250 µg/mL) 65 (62.5 µg/mL) 31 (15.6 µg/mL)
**6**	76 (250 µg/mL) 70 (62.5 µg/mL) 19 (15.6 µg/mL)	73 (250 µg/mL) 59 (62.5 µg/mL) 40 (15.6 µg/mL)
Microporenic acid A	83 (250 µg/mL) 81 (62.5 µg/mL) 48 (15.6 µg/mL)	71 (250 µg/mL) 70 (62.5 µg/mL) 39 (15.6 µg/mL)

n.t: not tested, (-) no activity.

## Supplementary Materials

Tables S1–S5, 1D, 2D NMR, ESIMS and HR-ESIMS spectra of compounds **1**–**6**; NMR spectra of intermediates in the synthesis of **6**. Protocol: Antimicrobial assay; Protocol: Cytotoxicity assay.
