# Supplementary material for: Macrooxazoles A–D, New 2,5-Disubstituted Oxazole-4-Carboxylic Acid Derivatives from the Plant Pathogenic Fungus Phoma macrostoma
Source: Molecules. 2020 Nov 24;25(23):5497. doi: 10.3390/molecules25235497 (PMC7727655; doi:10.3390/molecules25235497)
Supplement: Supplementary file 1 [file molecules-25-05497-s001.pdf]

Macrooxazoles A-D, new 2,5-disubstituted oxazole-4-carboxylic acid derivatives from the plant pathogenic fungus *Phoma macrostoma*

Blondelle Matio Kemkuignou [1,2,†], Laura Treiber [3,†], Haoxuan Zeng [1,2], Hedda Schrey [1,2] , Rainer Schobert [3], and Marc Stadler [1,2,*]

[1] Department of Microbial Drugs, Helmholtz Centre for Infection Research GmbH, Inhoffenstrasse 7, 38124 Braunschweig, Germany; blondelle.matiokemkuignou@helmholtz-hzi.de (B.M.K.), haoxuan.zeng@helmholtz-hzi.de (H.Z.), hedda.schrey@helmholtz-hzi.de (H.S.)

[2] German Centre for Infection Research (DZIF), partner site Hannover-Braunschweig, Inhoffenstrasse 7, 38124 Braunschweig, Germany

[3] Organic chemistry laboratory, University of Bayreuth, Universitaetsstrasse 30, 95447 Bayreuth, Germany; Rainer.Schobert@uni-bayreuth.de (R.S.), Laura1.Treiber@uni-bayreuth.de (L.T.)

* Correspondence: marc.stadler@helmholtz-hzi.de; Tel.: +49-531-6181-4240; Fax: +49-531-6181-9499

[†] These authors contributed equally.

Contents

Figure S1: HR-ESIMS data for macrooxazole A (**1**). 4
Figure S2: ESIMS data for macrooxazole A (**1**). 5
Figure S3: ^{1}H NMR spectrum (MeOH-*d4*, 500 MHz) of macrooxazole A (**1**). 6
Figure S4: ^{13}C NMR spectrum (MeOH-*d4*, 125 MHz) of macrooxazole A (**1**). 7
Figure S5: ^{1}H, ^{1}H COSY NMR spectrum (MeOH-*d4*, 500 MHz) of macrooxazole A (**1**). 8
Figure S6: ^{1}H, ^{1}H NOESY NMR spectrum (MeOH-*d4*, 500 MHz) of macrooxazole A (**1**). 9
Figure S7: ^{1}H, ^{13}C HSQC NMR spectrum (MeOH-*d4*, 500 MHz) of macrooxazole A (**1**). 10
Figure S8: ^{1}H, ^{13}C HMBC NMR spectrum (MeOH-*d4*, 500 MHz) of macrooxazole A (**1**). 11
Figure S9: ^{1}H, ^{15}N HMBC NMR spectrum (MeOH-*d4*, 500 MHz) of macrooxazole A (**1**). 12
Figure S10: HR-ESIMS data for macrooxazole B (**2**). 13
Figure S11: ESIMS data for macrooxazole B (**2**). 14
Figure S12: ^{1}H NMR spectrum (MeOH-*d4*, 500 MHz) of macrooxazole B (**2**). 15
Figure S13: ^{13}C NMR spectrum (MeOH-d4, 125 MHz) of macrooxazole B (**2**) 16
Figure S14: ^{1}H, ^{1}H COSY NMR spectrum (MeOH-*d4*, 500 MHz) of macrooxazole B (**2**). 17
Figure S15: ^{1}H, ^{13}C HSQC NMR spectrum (MeOH-*d4*, 500 MHz) of macrooxazole B (**2**). 18
Figure S16: ^{1}H, ^{13}C HMBC NMR spectrum (MeOH-*d4*, 500 MHz) of macrooxazole B (**2**). 19
Figure S17: HR-ESIMS data for macrooxazole C (**3**). 20
Figure S18: ESIMS data for macrooxazole C (**3**). 21
Figure S19: ^{1}H NMR spectrum (MeOH-*d4*, 500 MHz) of macrooxazole C (**3**). 22
Figure S20: ^{13}C NMR spectrum (MeOH-*d4*, 125 MHz) of macrooxazole C (**3**). 23
Figure S21: ^{1}H, ^{1}H COSY NMR spectrum (MeOH-*d4*, 500 MHz) of macrooxazole C (**3**). 24
Figure S22: ^{1}H, ^{13}C HSQC NMR spectrum (MeOH-*d4*, 500 MHz) of macrooxazole C (**3**). 25
Figure S23: 1H, 13C HMBC NMR spectrum (MeOH-d4, 500 MHz) of macrooxazole C (**3**). 26
Figure S24: HR-ESIMS data for macrooxazole D (**4**). 27
Figure S25: ESIMS data for macrooxazole D (**4**). 28
Figure S26: ^{1}H NMR spectrum (MeOH-*d4*, 500 MHz) of macrooxazole D (**4**). 29
Figure S27: ^{13}C NMR spectrum (MeOH-*d4*, 125 MHz) of macrooxazole D (**4**). 30
Figure S28: ^{1}H, ^{1}H COSY NMR spectrum (MeOH-*d4*, 500 MHz) of macrooxazole D (**4**). 31
Figure S29: ^{1}H, ^{13}C HSQC NMR spectrum (MeOH-*d4*, 500 MHz) of macrooxazole D (**4**). 32
Figure S30: ^{1}H, ^{13}C HMBC NMR spectrum (MeOH-*d4*, 500 MHz) of macrooxazole D (**4**). 33
Figure S31: HR-ESIMS data for macrocidin A (**5**) 34
Figure S32: ESIMS data for macrocidin A (**5**). 35
Figure S33: ^{1}H NMR spectrum (MeOH-*d4*, 500 MHz) of macrocidin A (**7**) 36
Figure S34: HR-ESIMS data for macrocidin Z (**6**). 37

Figure S35: ESIMS data for macrocidin Z (**6**). 38
Figure S36: ^{1}H NMR spectrum (MeOH-*d4*, 500 MHz) of macrocidin Z (**6**). 39
Figure S37: ^{13}C NMR spectrum (MeOH-*d4*, 125 MHz) of macrocidin Z (**6**). 40
Figure S38: ^{1}H, ^{1}H COSY NMR spectrum (MeOH-*d4*, 500 MHz) of macrocidin Z (**6**). 41
Figure S39: ^{1}H, ^{1}H ROESY NMR spectrum (MeOH-*d4*, 500 MHz) of macrocidin Z (**6**) 42
Figure S40: ^{1}H, ^{13}C HSQC NMR spectrum (MeOH-*d4*, 500 MHz) of macrocidin Z (**6**). 43
Figure S41: ^{1}H, ^{13}C HMBC NMR spectrum (MeOH-*d4*, 500 MHz) of macrocidin Z (**6**). 44
Table S1: Minimum Inhibitory concentrations (MIC) of compounds **1-6** against tested microorganisms. 45
Table S2: Cytotoxic effect (IC_{50}) of compound **1-6** against two cancer cell lines. 46
Figure S42: ^{1}H-NMR spectrum of compound **8** in $CDCl_3$. 47
Figure S43: ^{13}C-NMR spectrum of compound **8** in $CDCl_3$. 48
Figure S44: ^{1}H-NMR spectrum of compound **9** in $CDCl_3$. 49
Figure S45: ^{13}C-NMR spectrum of compound **9** in $CDCl_3$. 50
Figure S46: ^{1}H-NMR spectrum of compound **10** in $CDCl_3$. 51
Figure S47: ^{13}C-NMR spectrum of compound **10** in $CDCl_3$. 52
Figure S48: ^{1}H-NMR spectrum of compound **13** in $CDCl_3$. 53
Figure S49: ^{13}C-NMR spectrum of compound **13** in CDCl3 54
Figure S50: ^{1}H-NMR spectrum of compound **14** in MeOD. 55
Figure S51: ^{13}C-NMR spectrum of compound **14** in MeOD. 56
Figure S52: ^{1}H-NMR spectrum of compound **14** in $CDCl_3$. 57
Figure S53: ^{13}C-NMR spectrum of compound **14** in $CDCl_3$. 58
Figure S54: ^{1}H-NMR spectrum of compound **15** in MeOD. 59
Figure S55: ^{13}C-NMR spectrum of compound **15** in MeOD. 60
Figure S56: ^{1}H-NMR spectrum of compound **15** in $CDCl_3$. 61
Figure S57: ^{13}C-NMR spectrum of compound **15** in $CDCl_3$. 62
Figure S58: ^{1}H-NMR spectrum of macrocidin Z (**6**) in MeOD. 63
Figure S59: ^{13}C-NMR spectrum of macrocidin Z (**6**) in MeOD. 64
Figure S60: ^{1}H-NMR spectrum of macrocidin Z (**6**) in $CDCl_3$. 65
Figure S61: ^{13}C-NMR spectrum of macrocidin Z (**6**) in $CDCl_3$. 66
Table S3: MIC assay experiment parameters 67
Table S4: Cytotoxicity assay experiment parameters 69
Table S5: *S. aureus* biofilm and preformed biofilm inhibition activity of compounds **1-3**, **5-6** 69

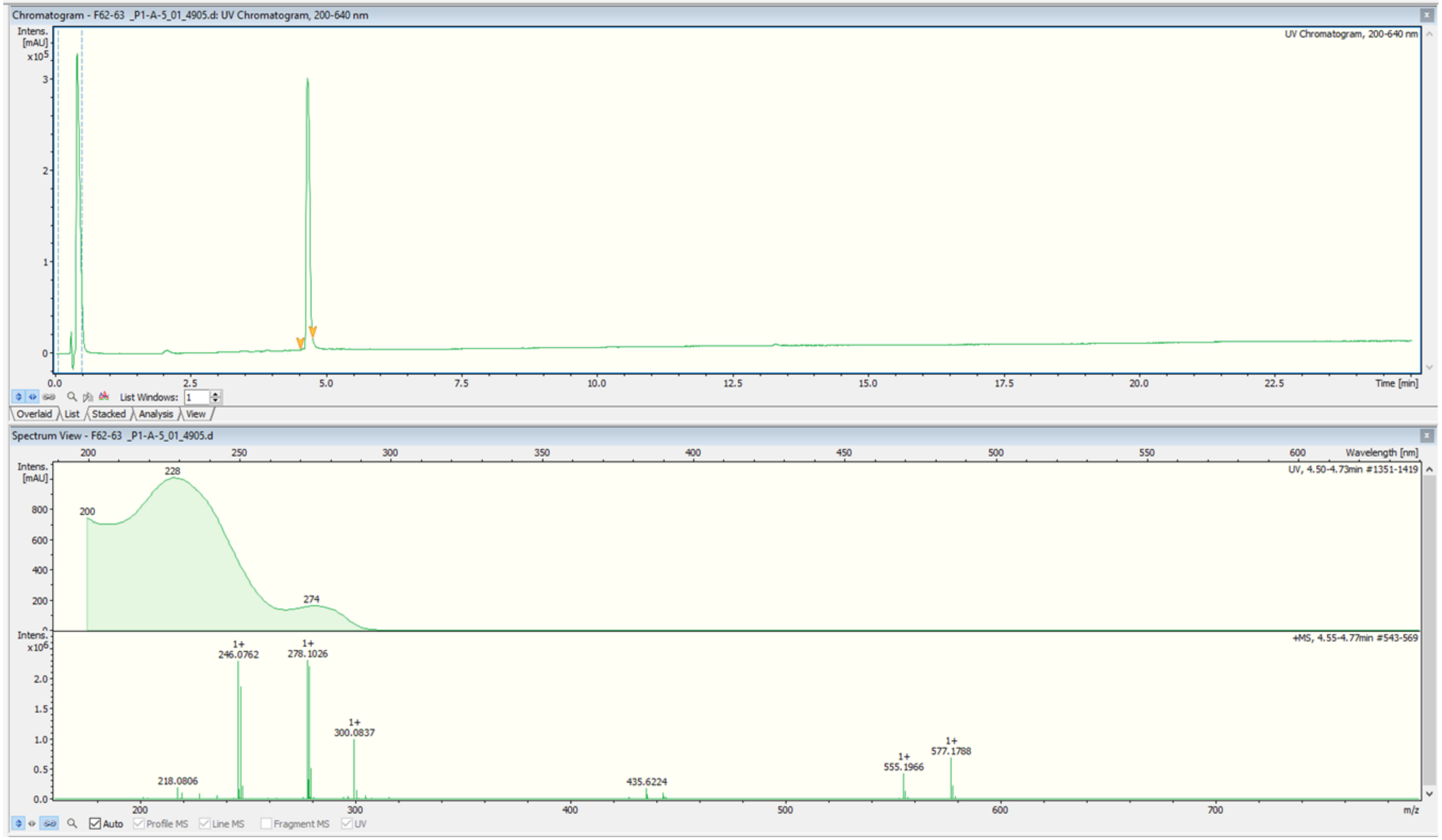

Figure S1: HR-ESIMS data for macrooxazole A (**1**).

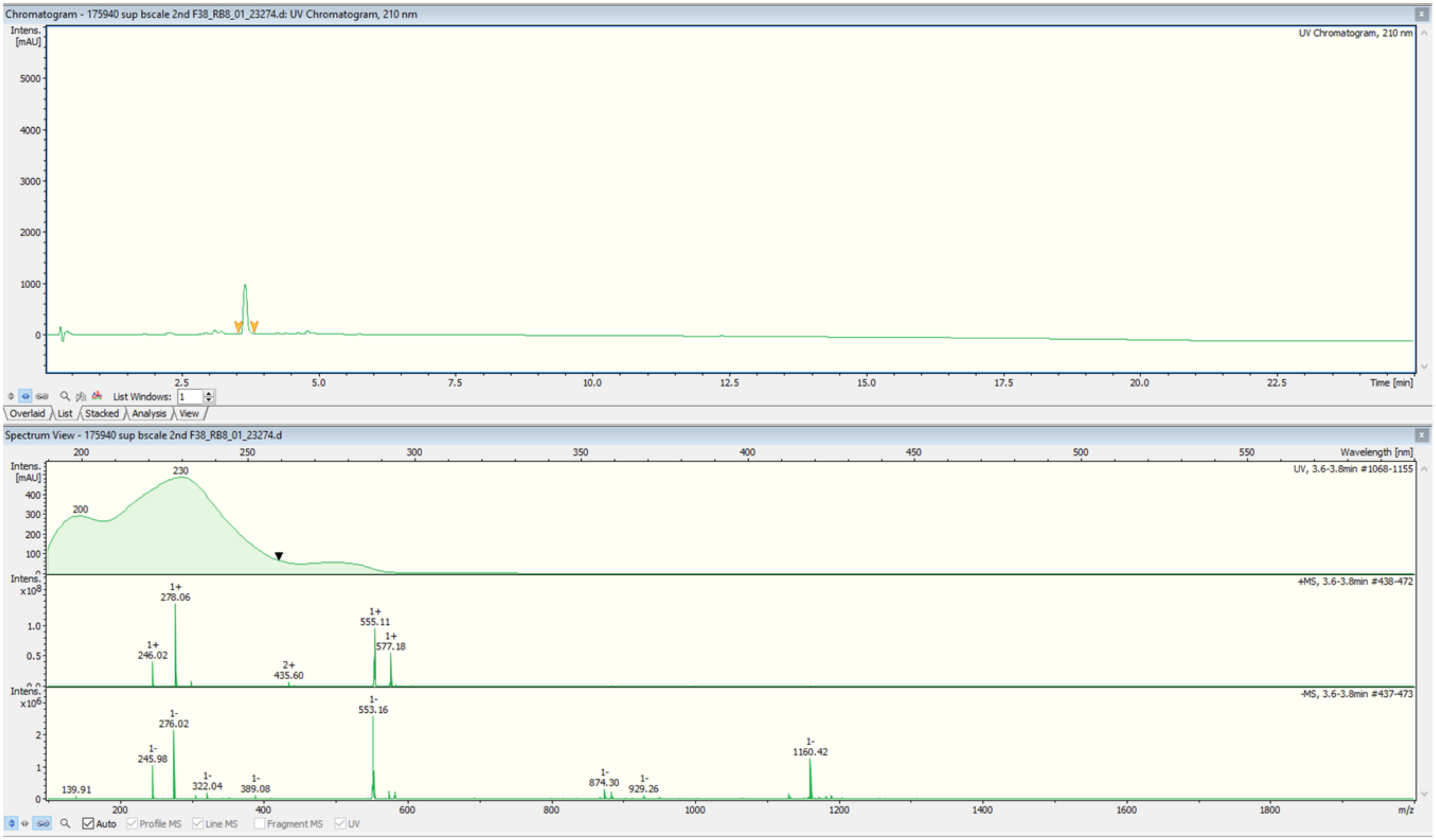

Figure S2: ESIMS data for macrooxazole A (**1**).

1 D and 2D NMR data for macrooxazole A (1)

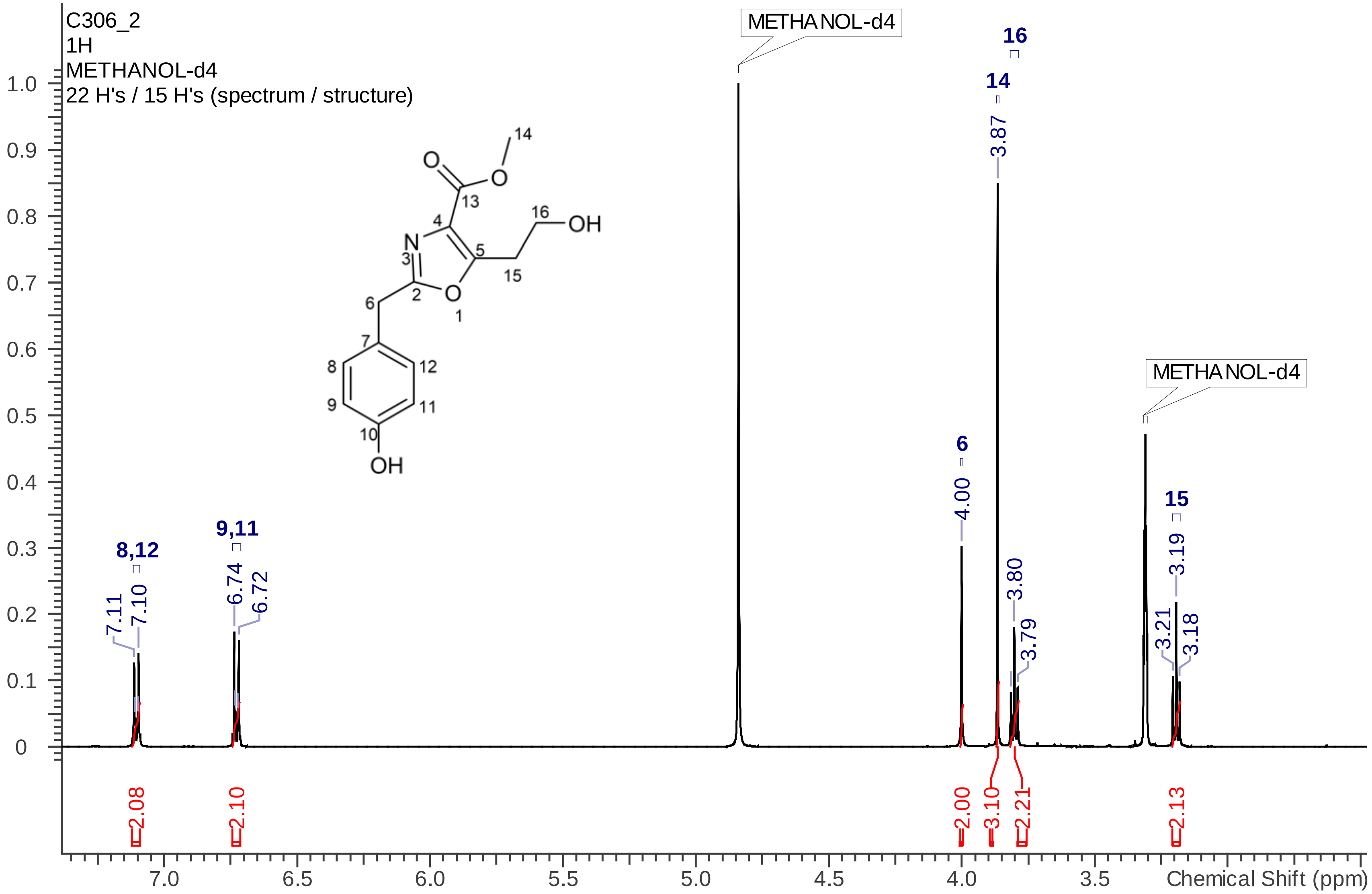

Figure S3: ^{1}H NMR spectrum (MeOH-*d4*, 500 MHz) of macrooxazole A (**1**).

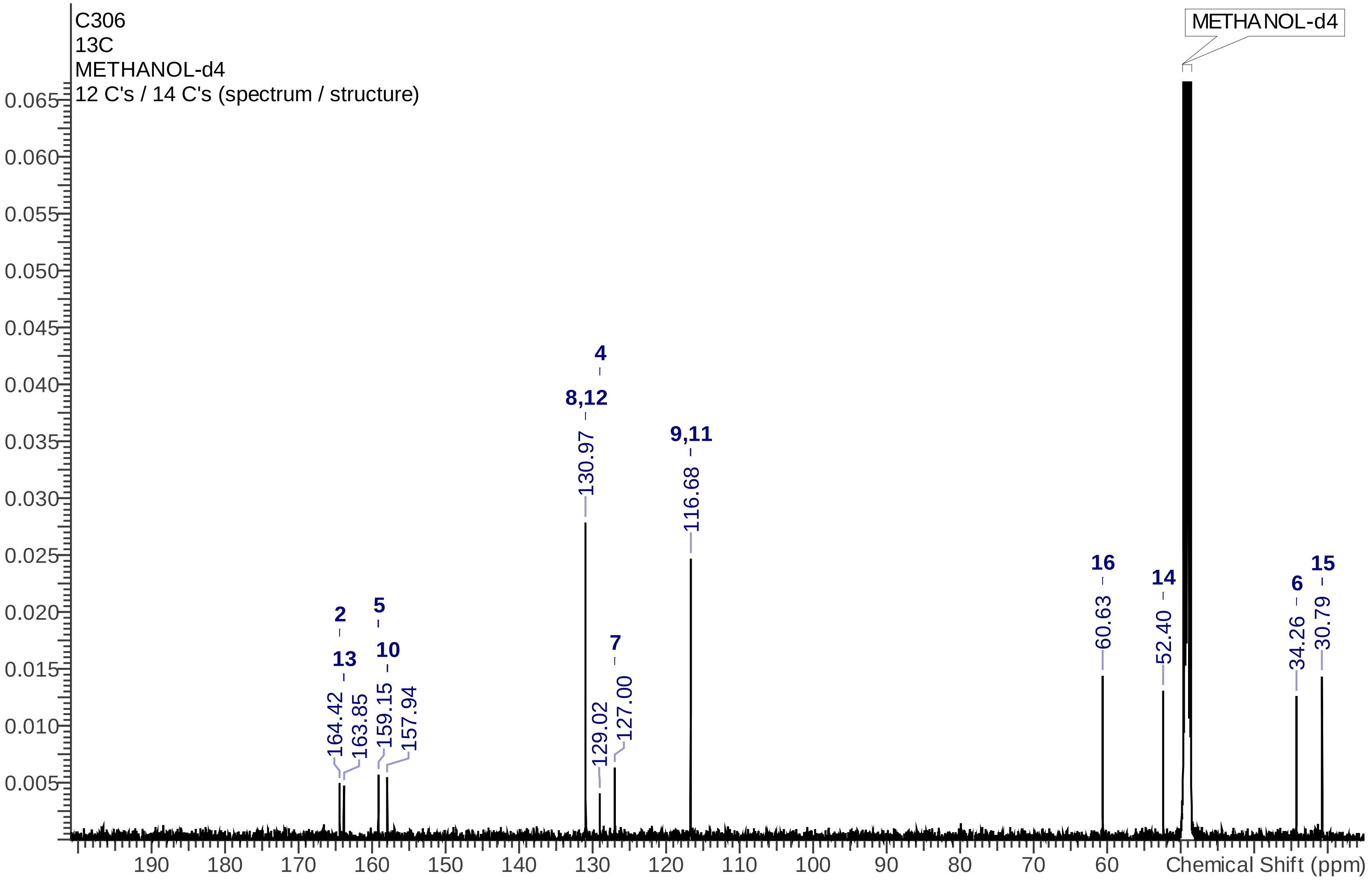

Figure S4: ^{13}C NMR spectrum (MeOH-*d4*, 125 MHz) of macrooxazole A (**1**).

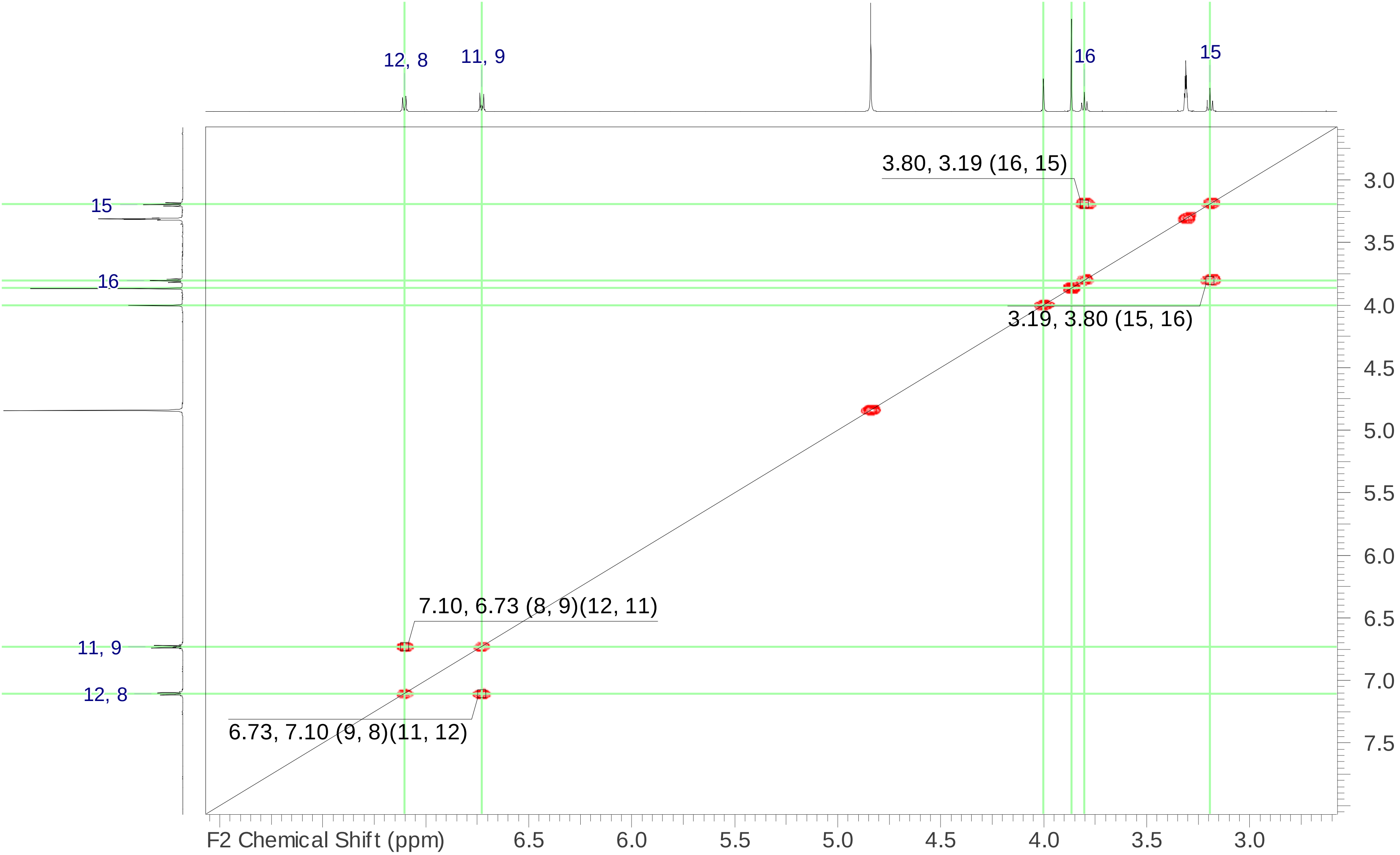

Figure S5: ^{1}H, ^{1}H COSY NMR spectrum (MeOH-*d4*, 500 MHz) of macrooxazole A (**1**).

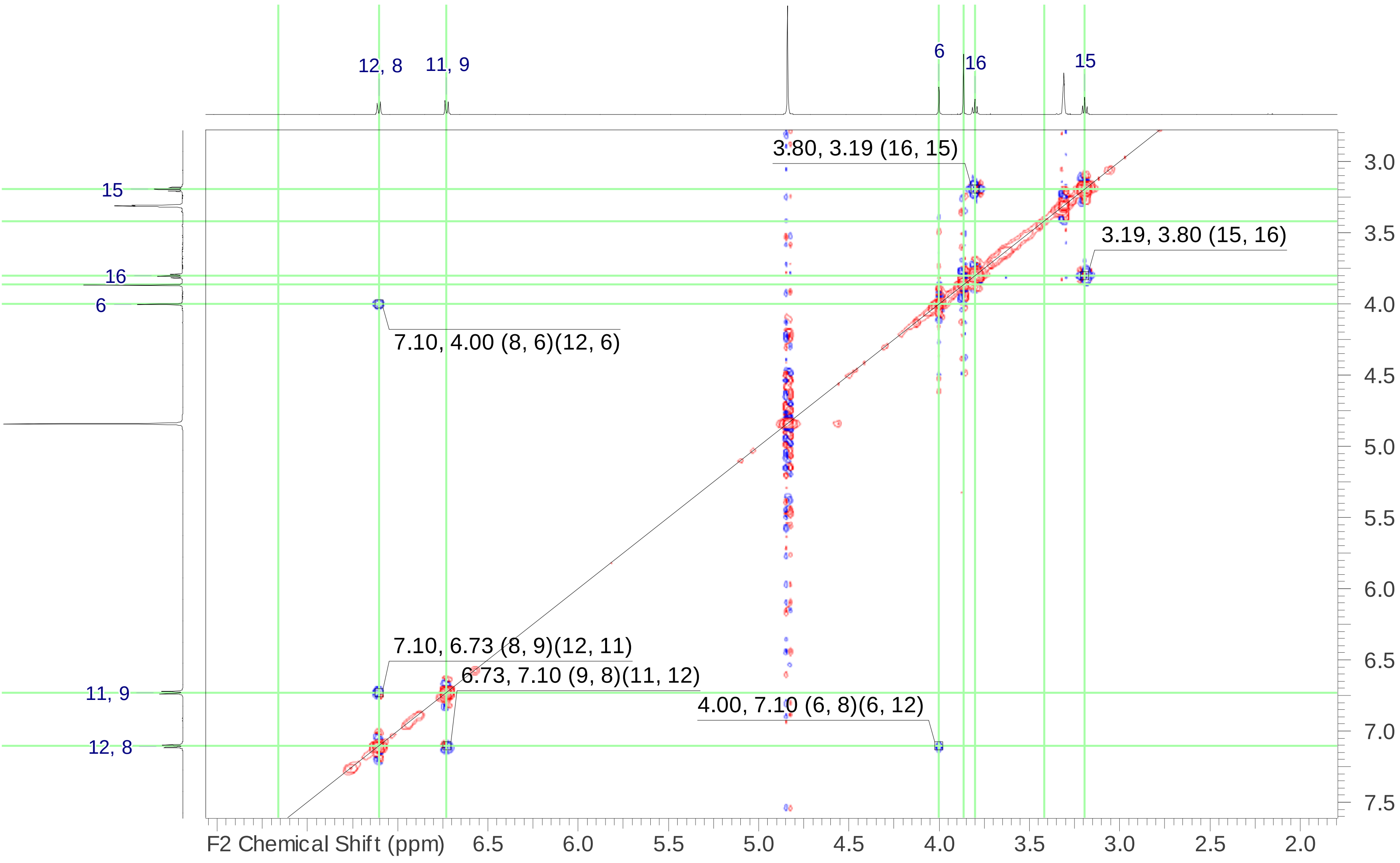

Figure S6: ^{1}H, ^{1}H NOESY NMR spectrum (MeOH-*d4*, 500 MHz) of macrooxazole A (**1**).

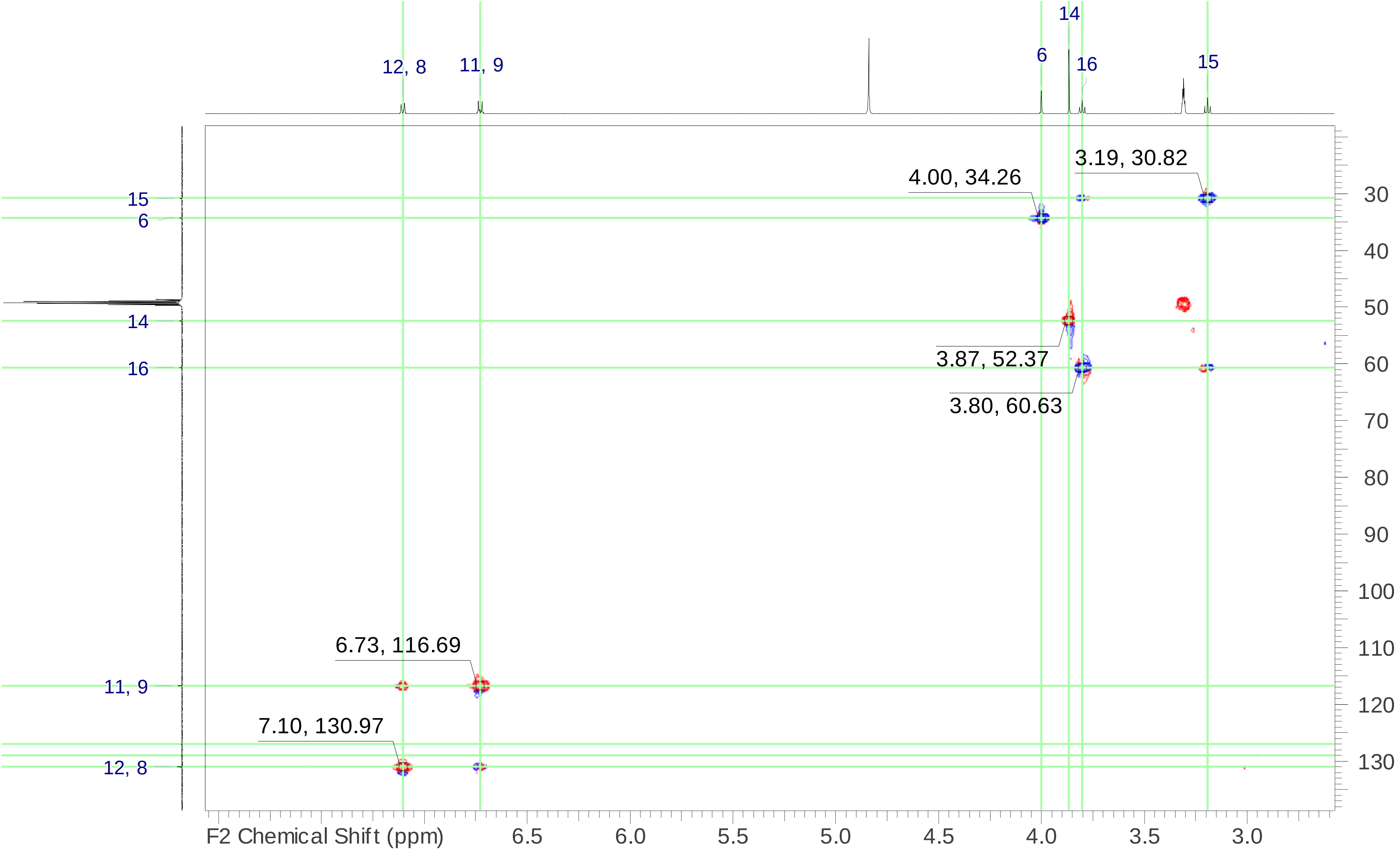

Figure S7: ^{1}H, ^{13}C HSQC NMR spectrum (MeOH-*d4*, 500 MHz) of macrooxazole A (**1**).

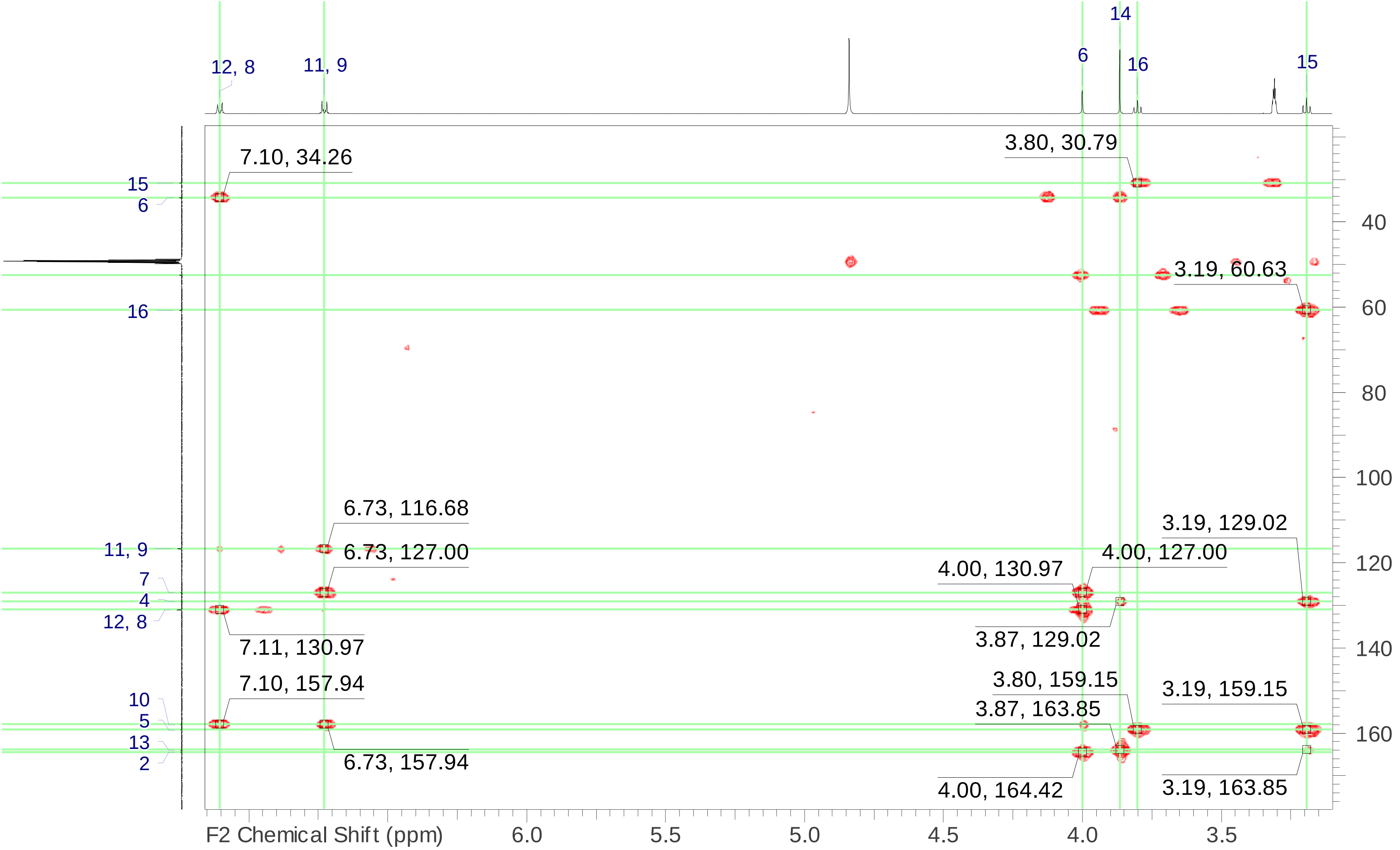

Figure S8: ^{1}H, ^{13}C HMBC NMR spectrum (MeOH-*d4*, 500 MHz) of macrooxazole A (**1**).

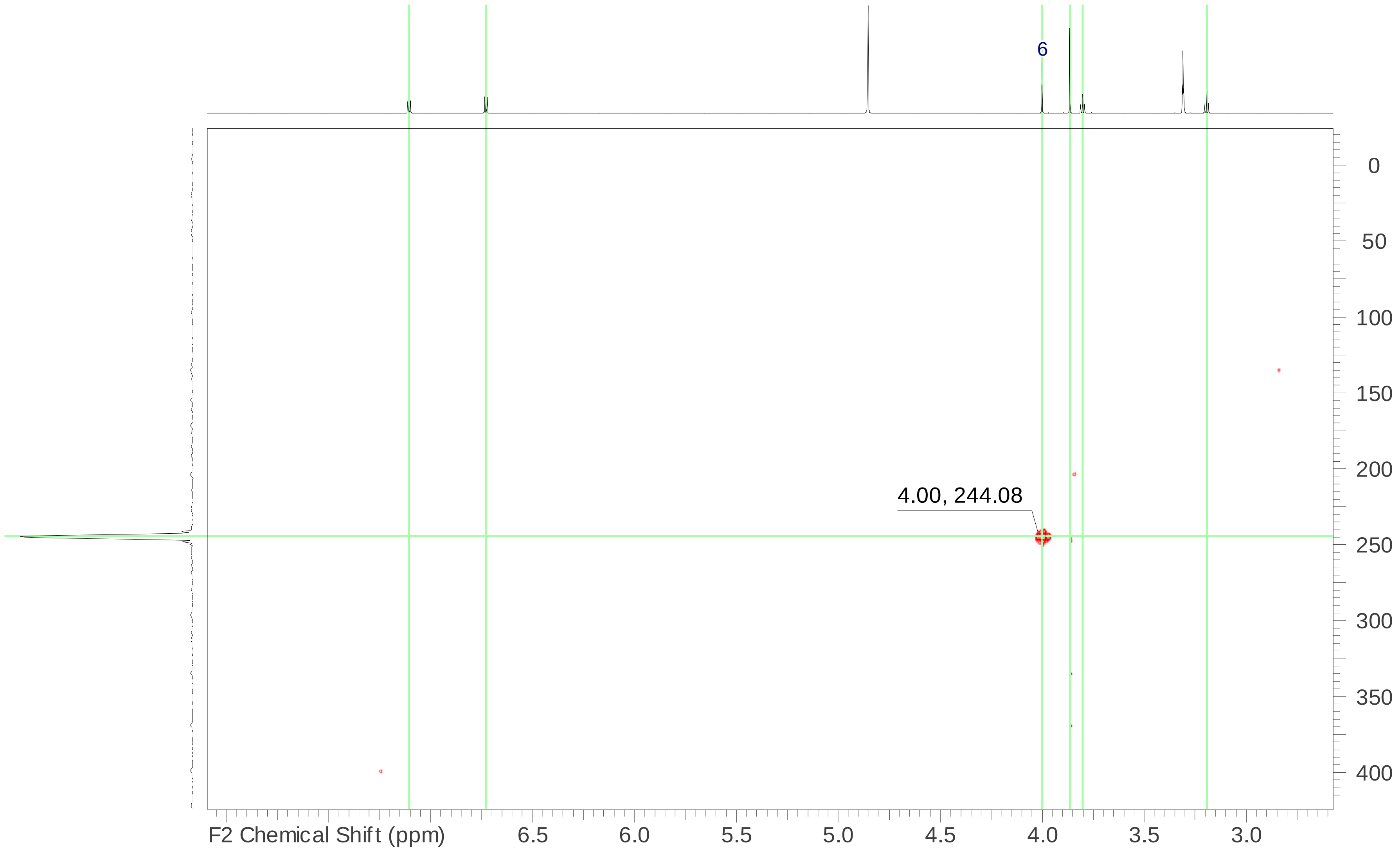

Figure S9: ^{1}H, ^{15}N HMBC NMR spectrum (MeOH-*d4*, 500 MHz) of macrooxazole A (**1**).

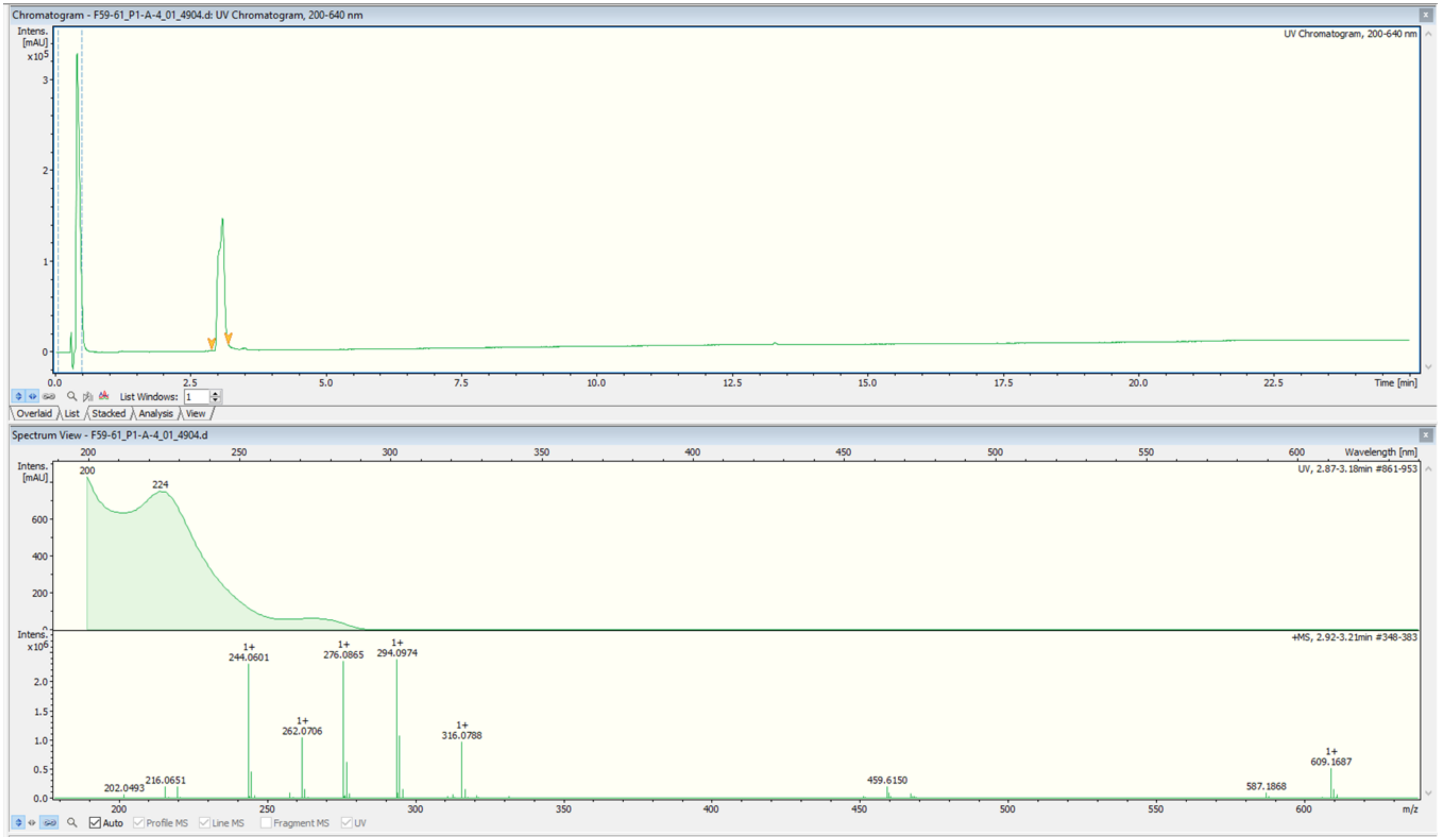

Figure S10: HR-ESIMS data for macrooxazole B (**2**).

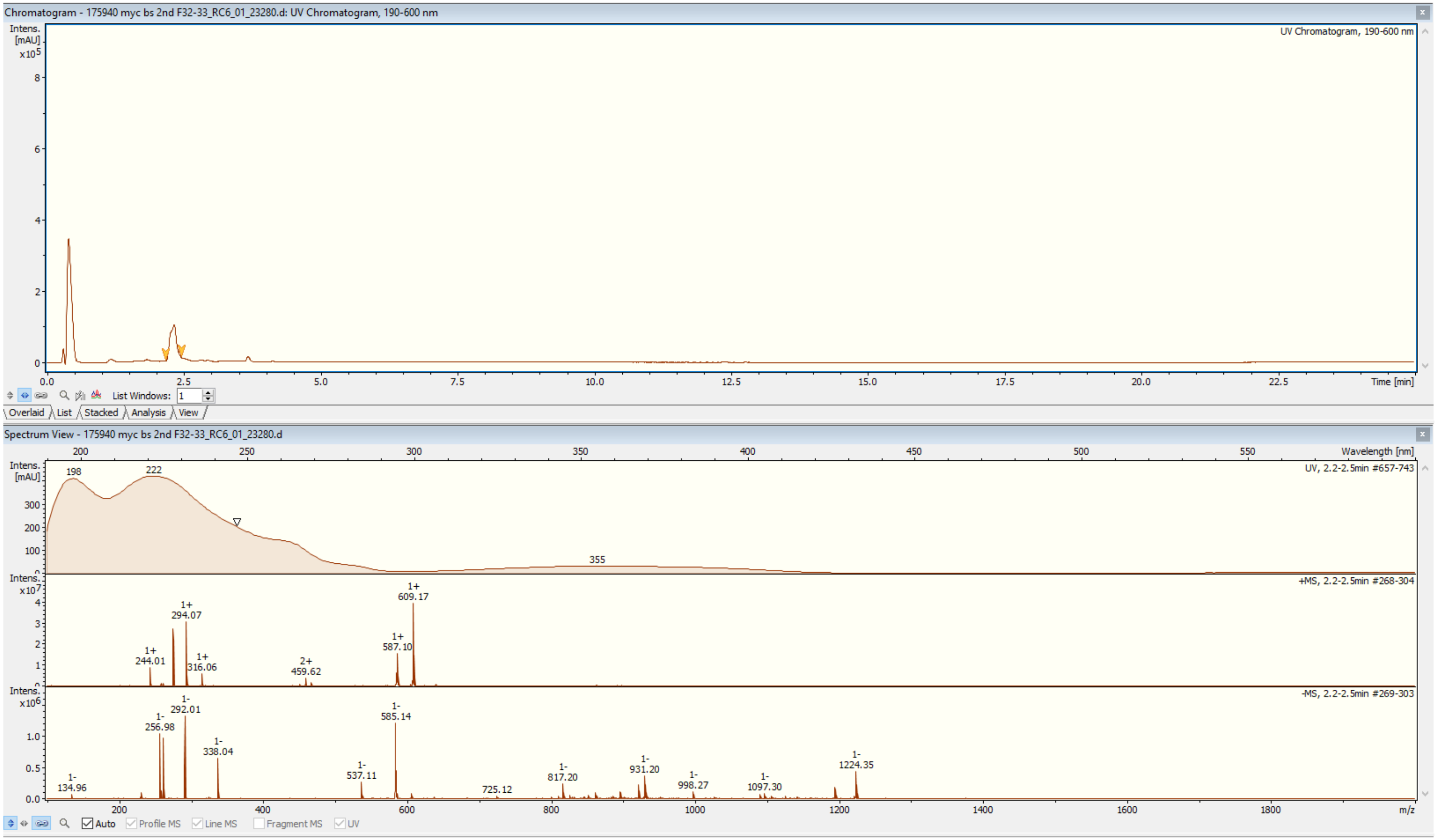

Figure S11: ESIMS data for macrooxazole B (**2**).

1D and 2D NMR data for macrooxazole B (2)

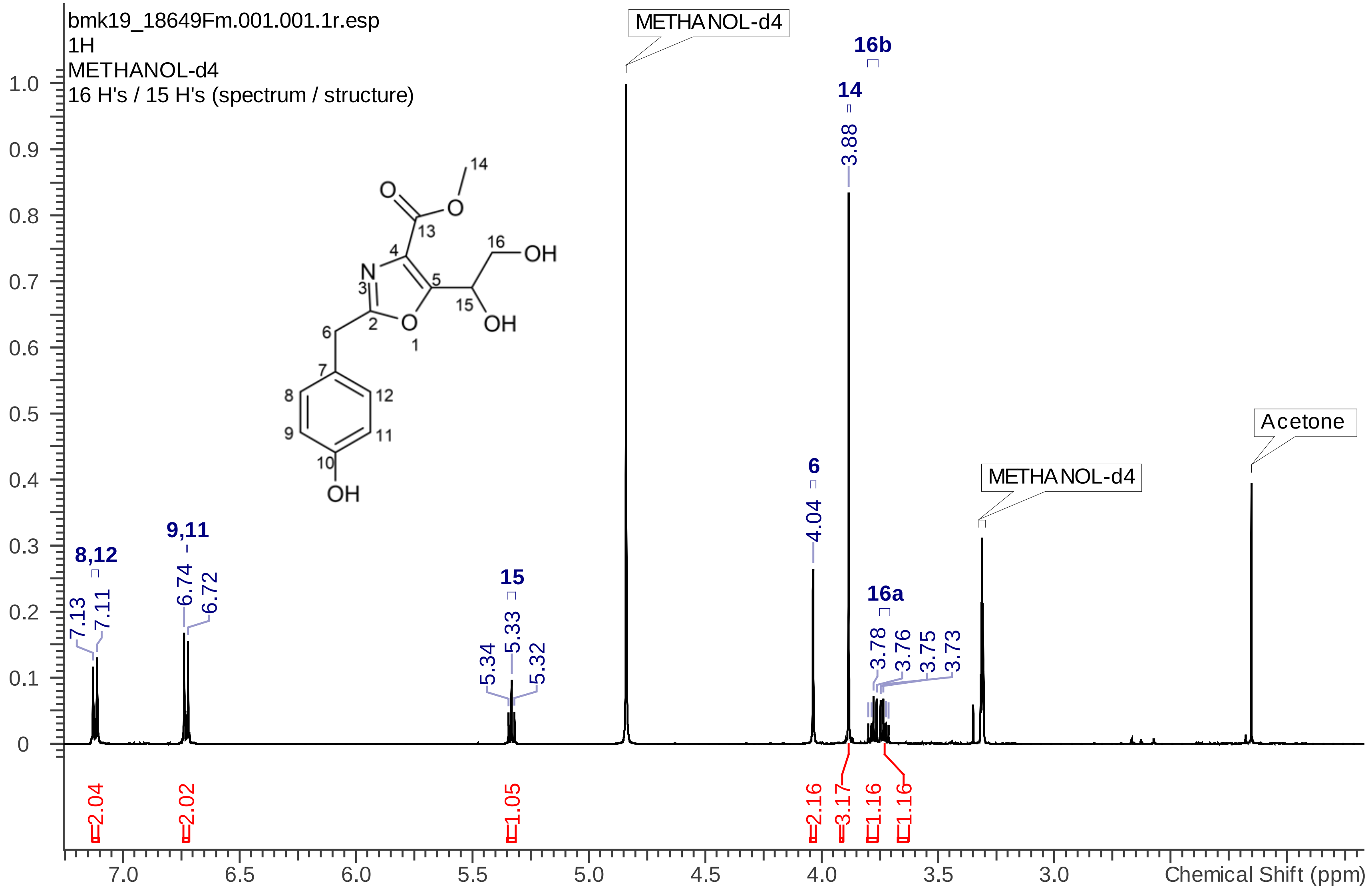

Figure S12: ^{1}H NMR spectrum (MeOH-*d4*, 500 MHz) of macrooxazole B (**2**).

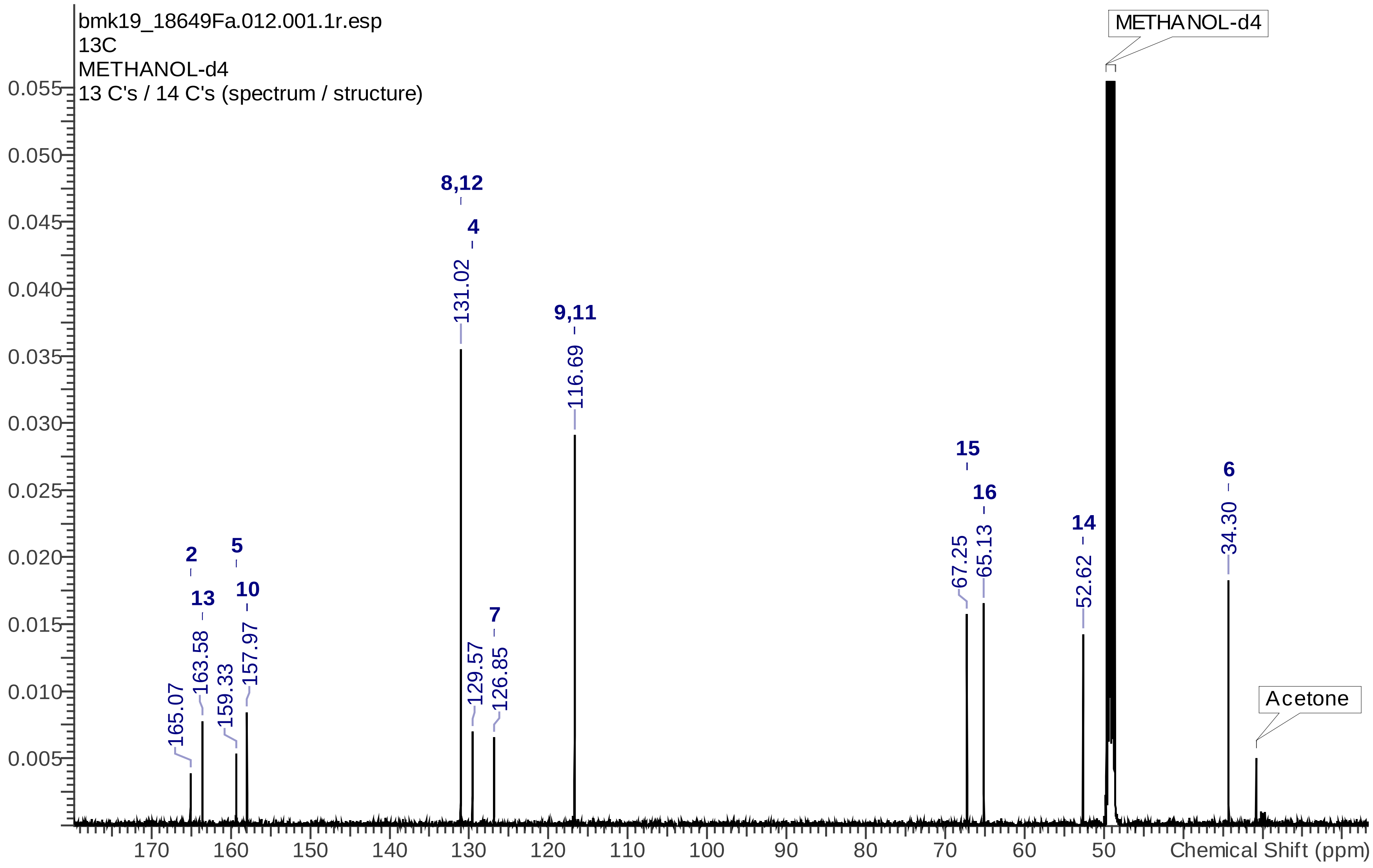

Figure S13: 13C NMR spectrum (MeOH-d4, 125 MHz) of macrooxazole B (**2**).

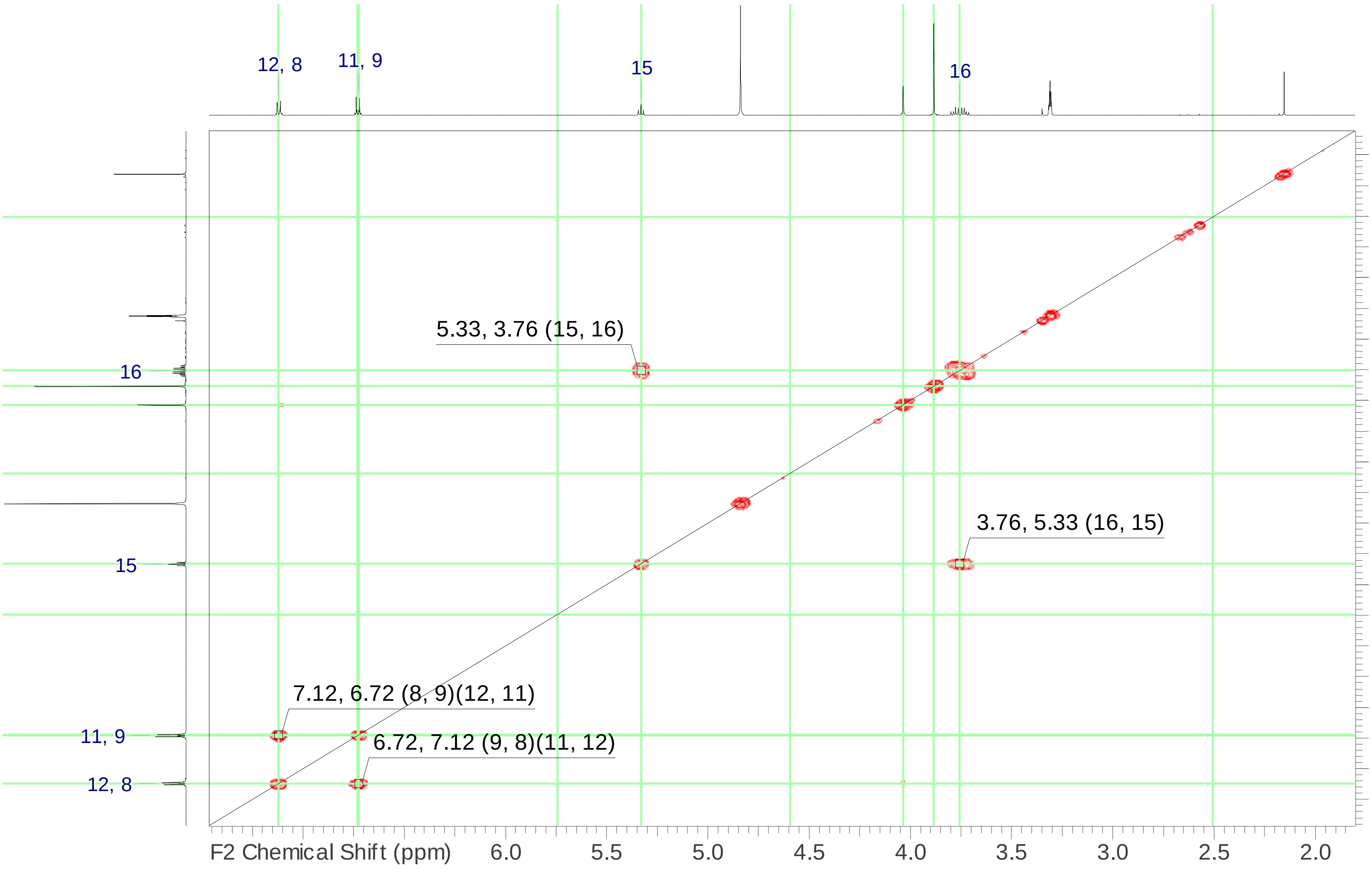

Figure S14: ^{1}H, ^{1}H COSY NMR spectrum (MeOH-*d4*, 500 MHz) of macrooxazole B (**2**).

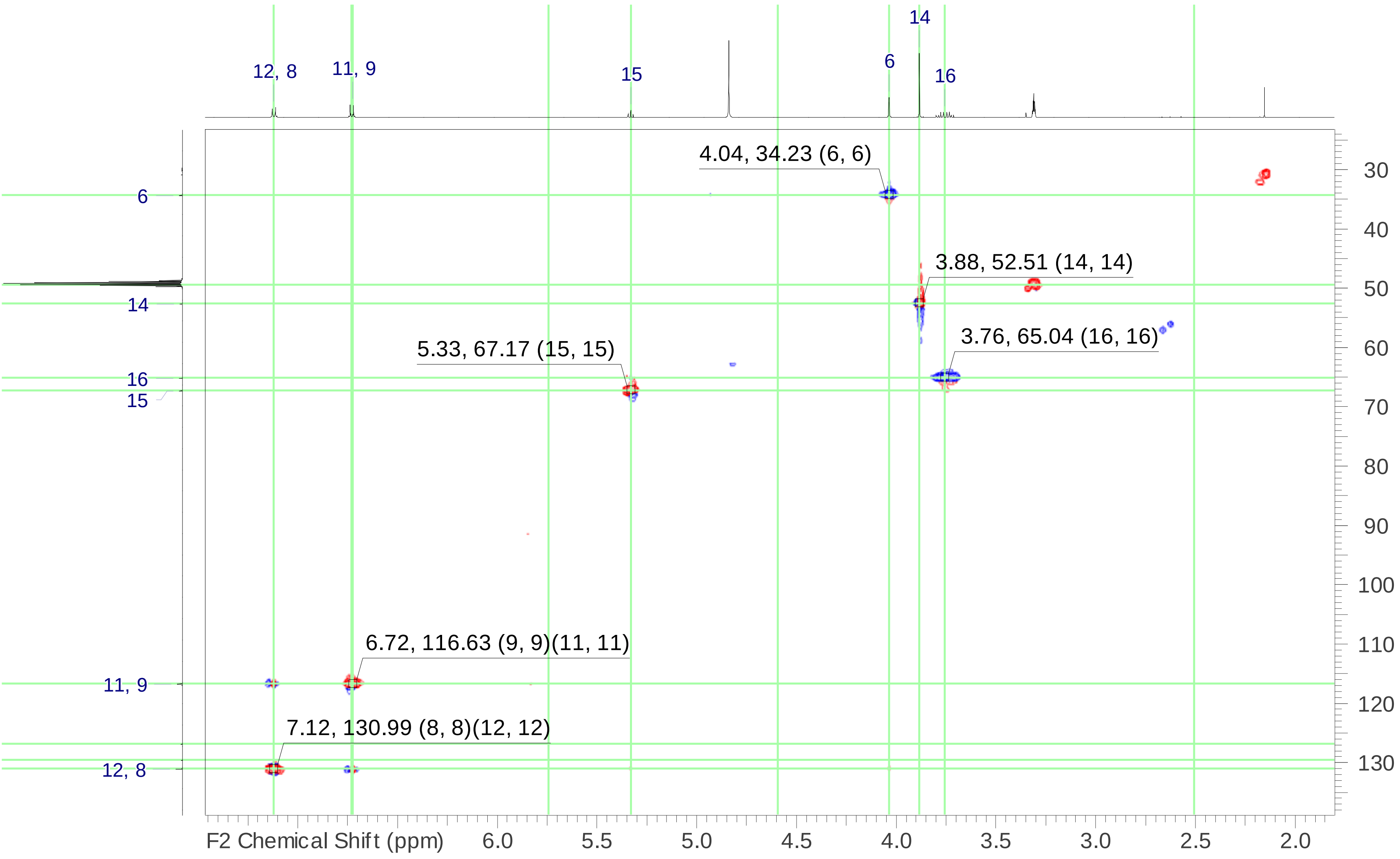

Figure S15: ^{1}H, ^{13}C HSQC NMR spectrum (MeOH-*d4*, 500 MHz) of macrooxazole B (**2**).

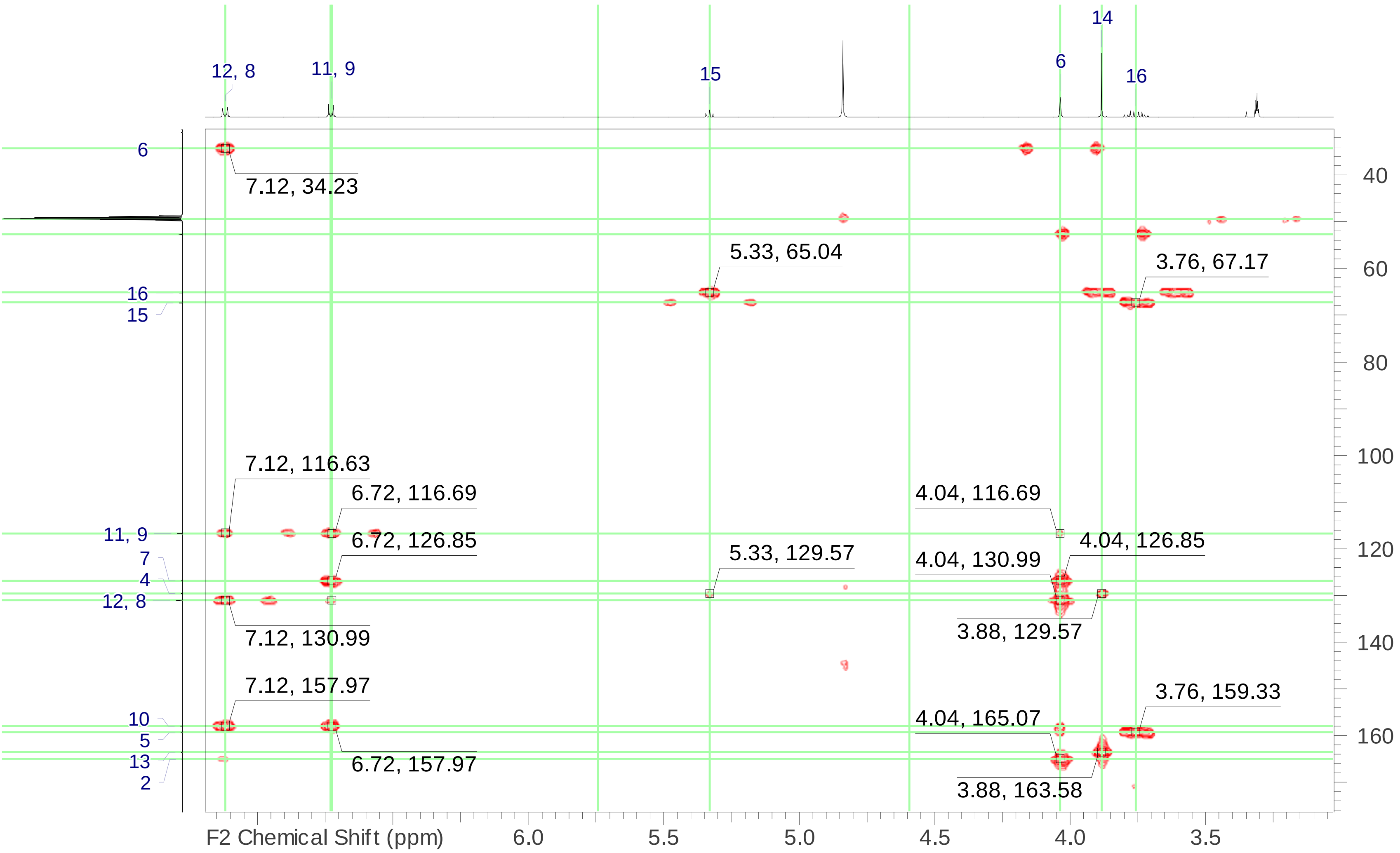

Figure S16: ^{1}H, ^{13}C HMBC NMR spectrum (MeOH-*d4*, 500 MHz) of macrooxazole B (**2**).

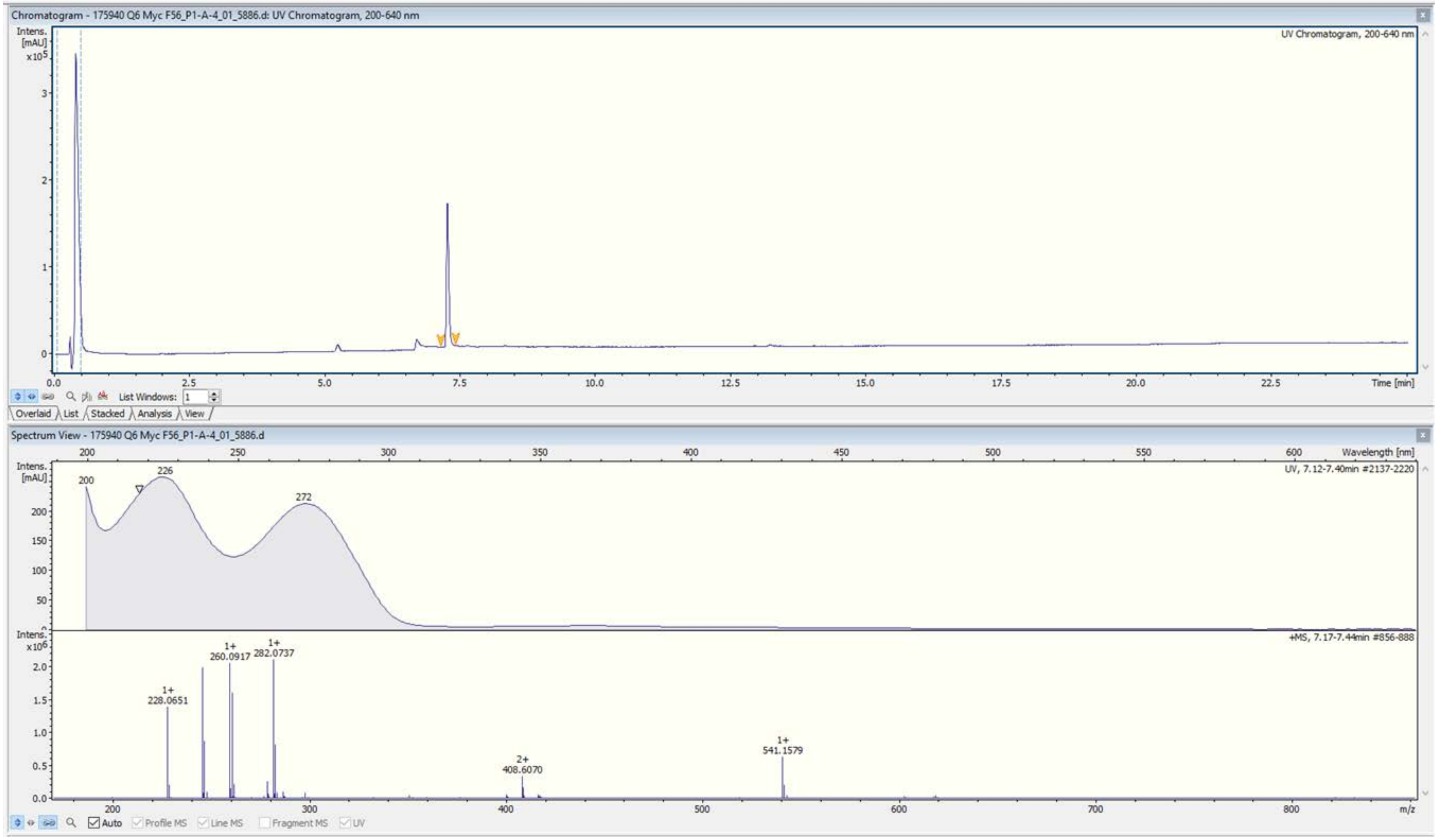

Figure S17: HR-ESIMS data for macrooxazole C (**3**).

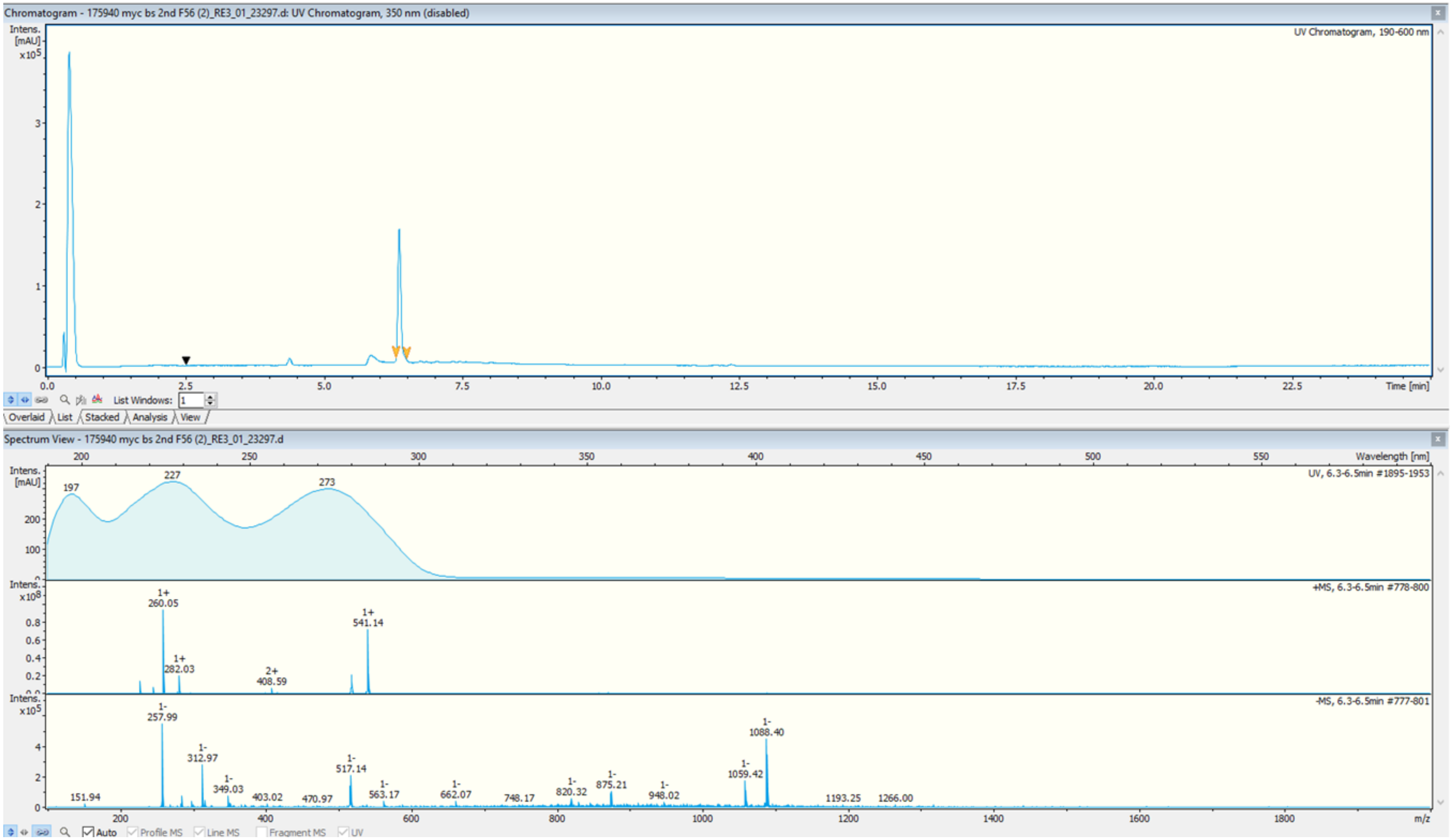

Figure S18: ESIMS data for macrooxazole C (**3**).

1D and 2D NMR data for macrooxazole C (3)

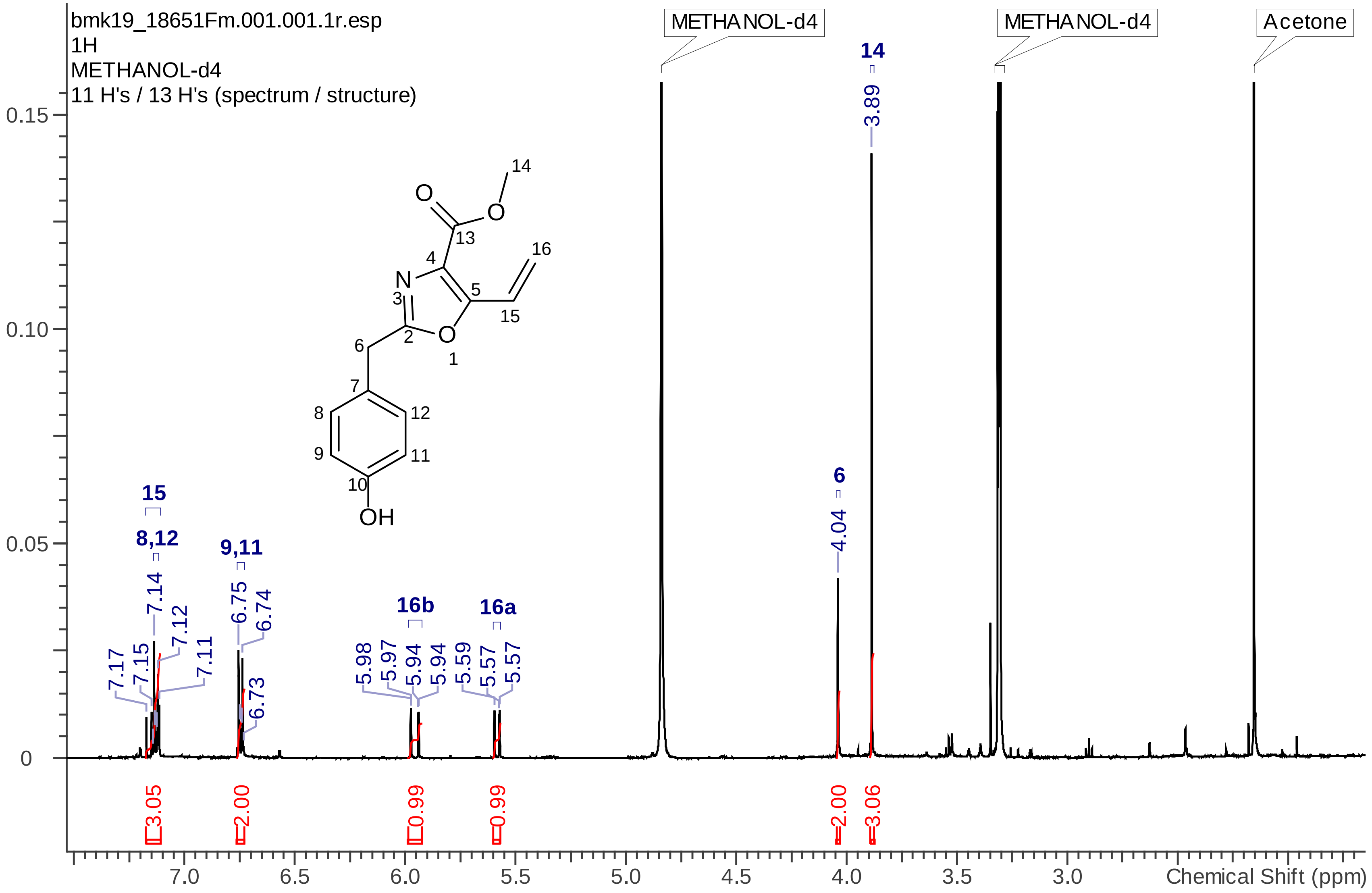

Figure S19: ^{1}H NMR spectrum (MeOH-*d4*, 500 MHz) of macrooxazole C (**3**).

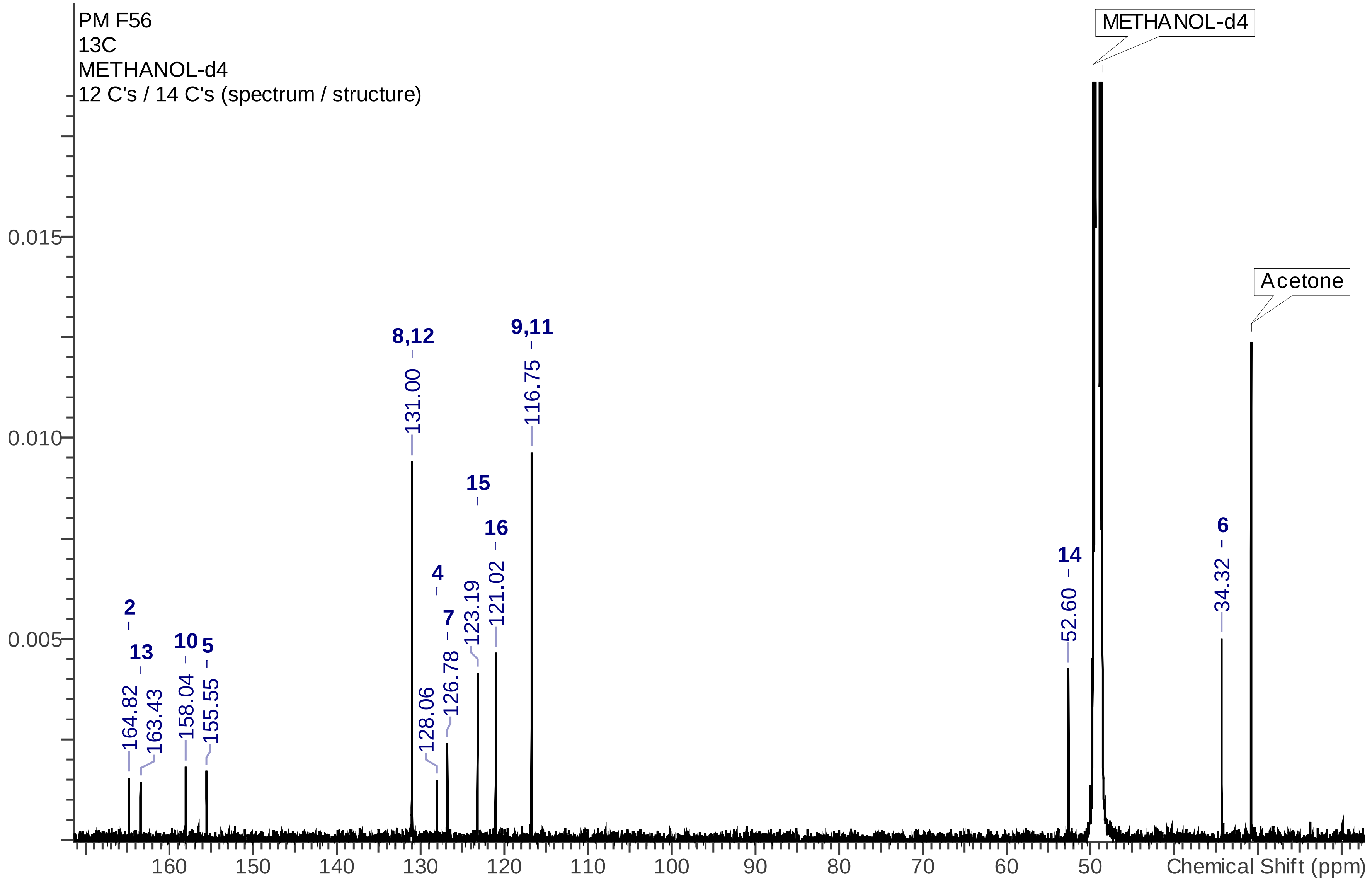

Figure S20: ^{13}C NMR spectrum (MeOH-*d4*, 125 MHz) of macrooxazole C (**3**).

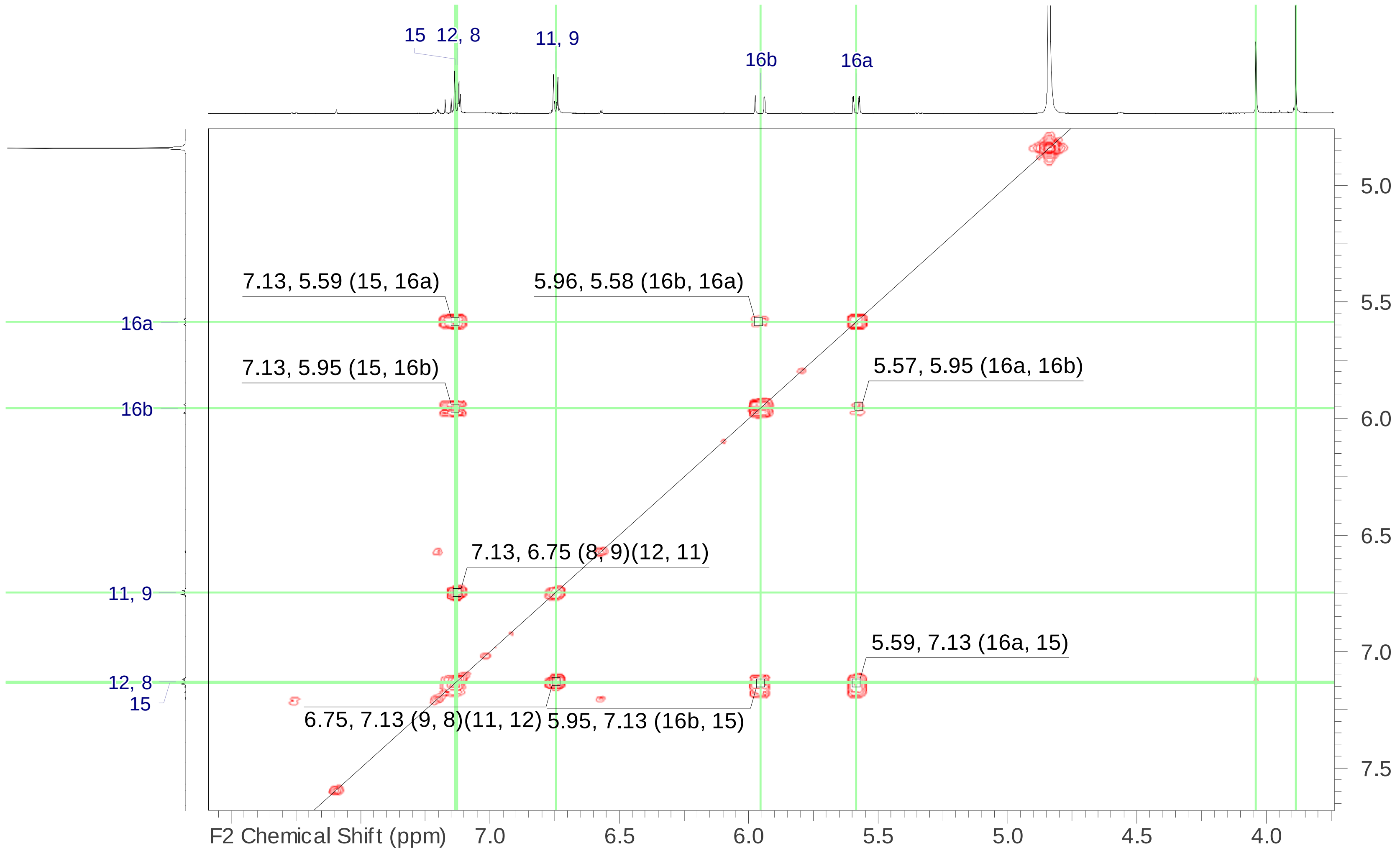

Figure S21: ^{1}H, ^{1}H COSY NMR spectrum (MeOH-*d4*, 500 MHz) of macrooxazole C (**3**).

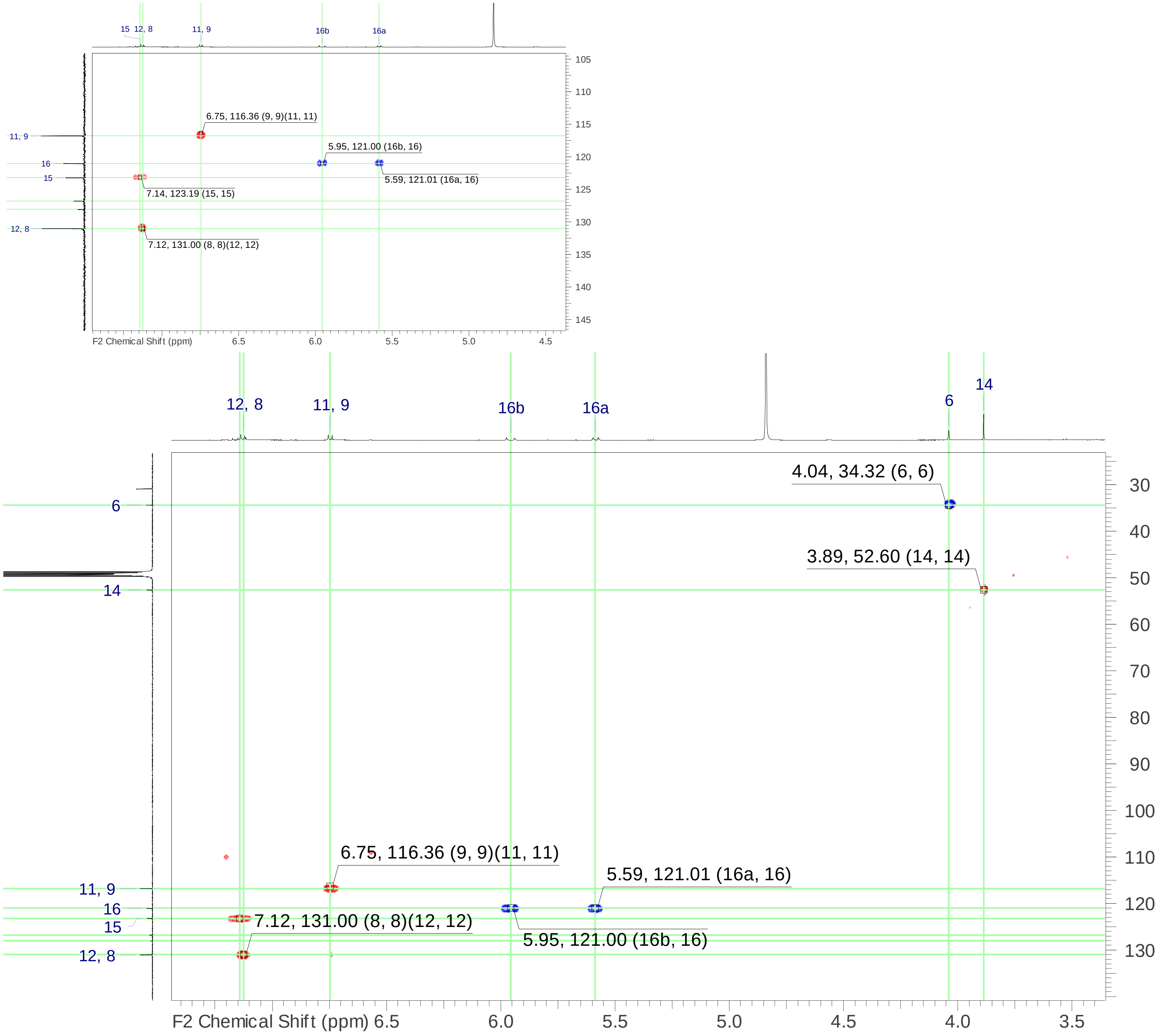

Figure S22: ^{1}H, ^{13}C HSQC NMR spectrum (MeOH-*d4*, 500 MHz) of macrooxazole C (**3**).

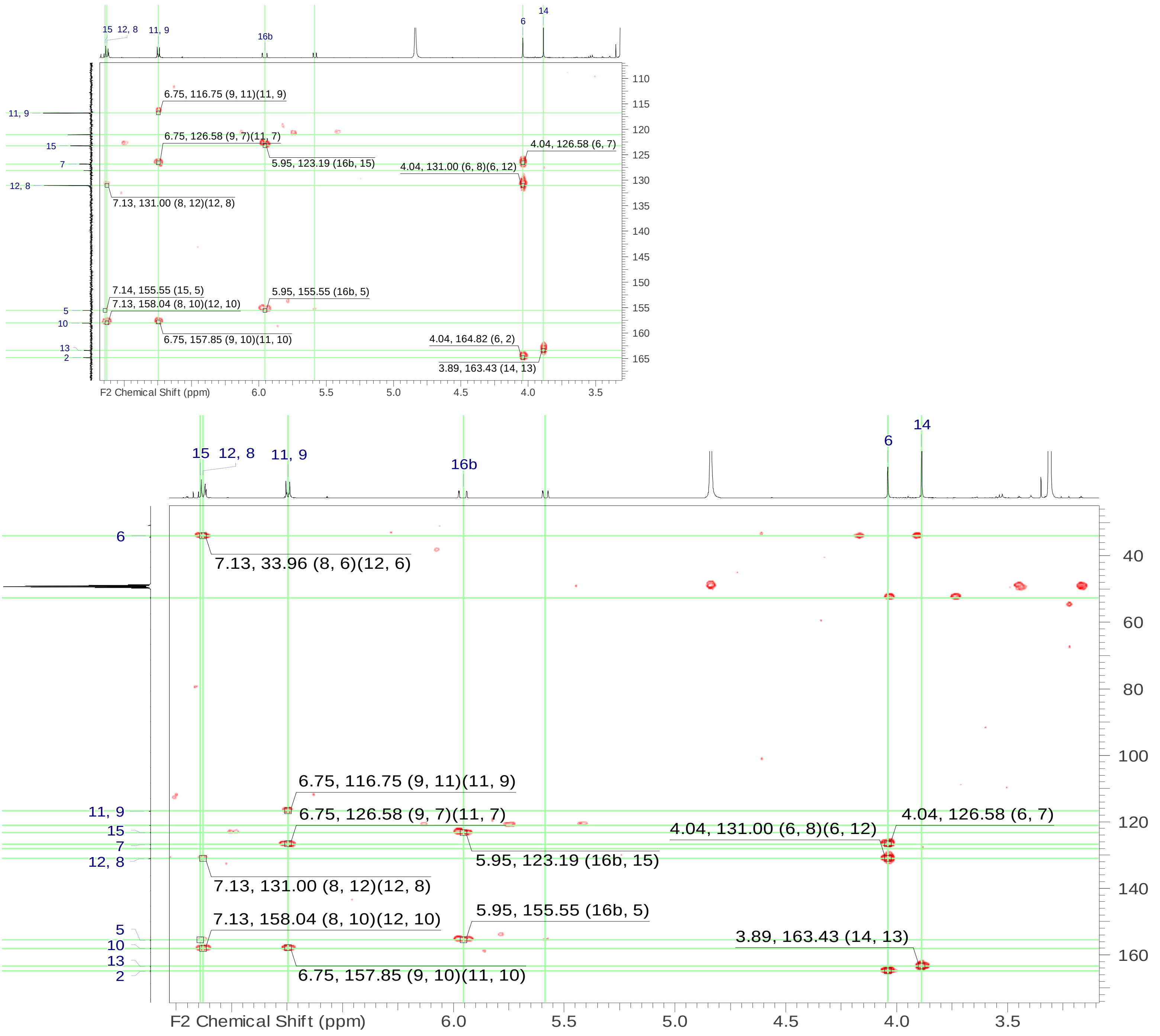

Figure S23: ^{1}H, ^{13}C HMBC NMR spectrum (MeOH-*d4*, 500 MHz) of macrooxazole C (**3**).

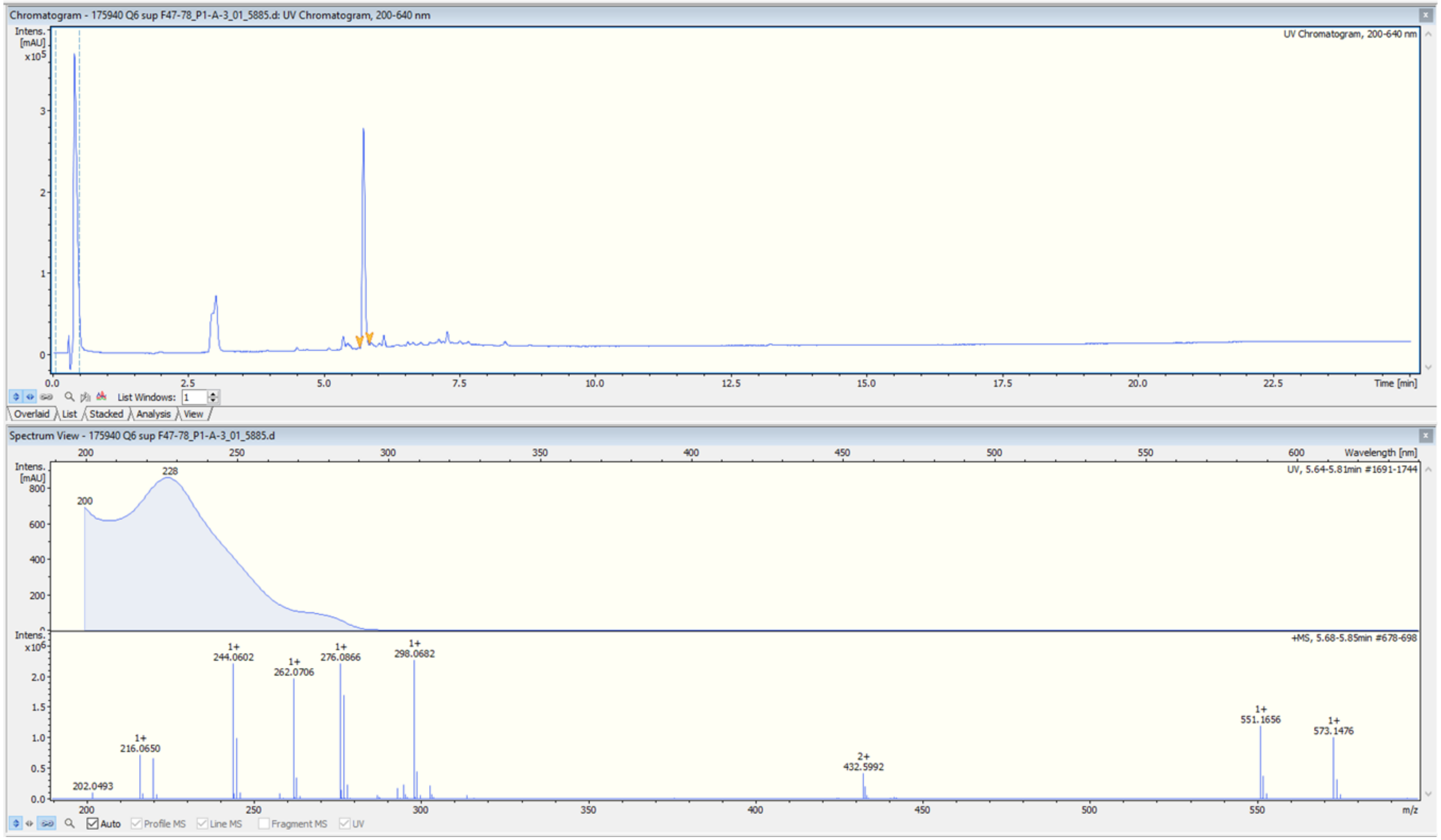

Figure S24: HR-ESIMS data for macrooxazole D (**4**).

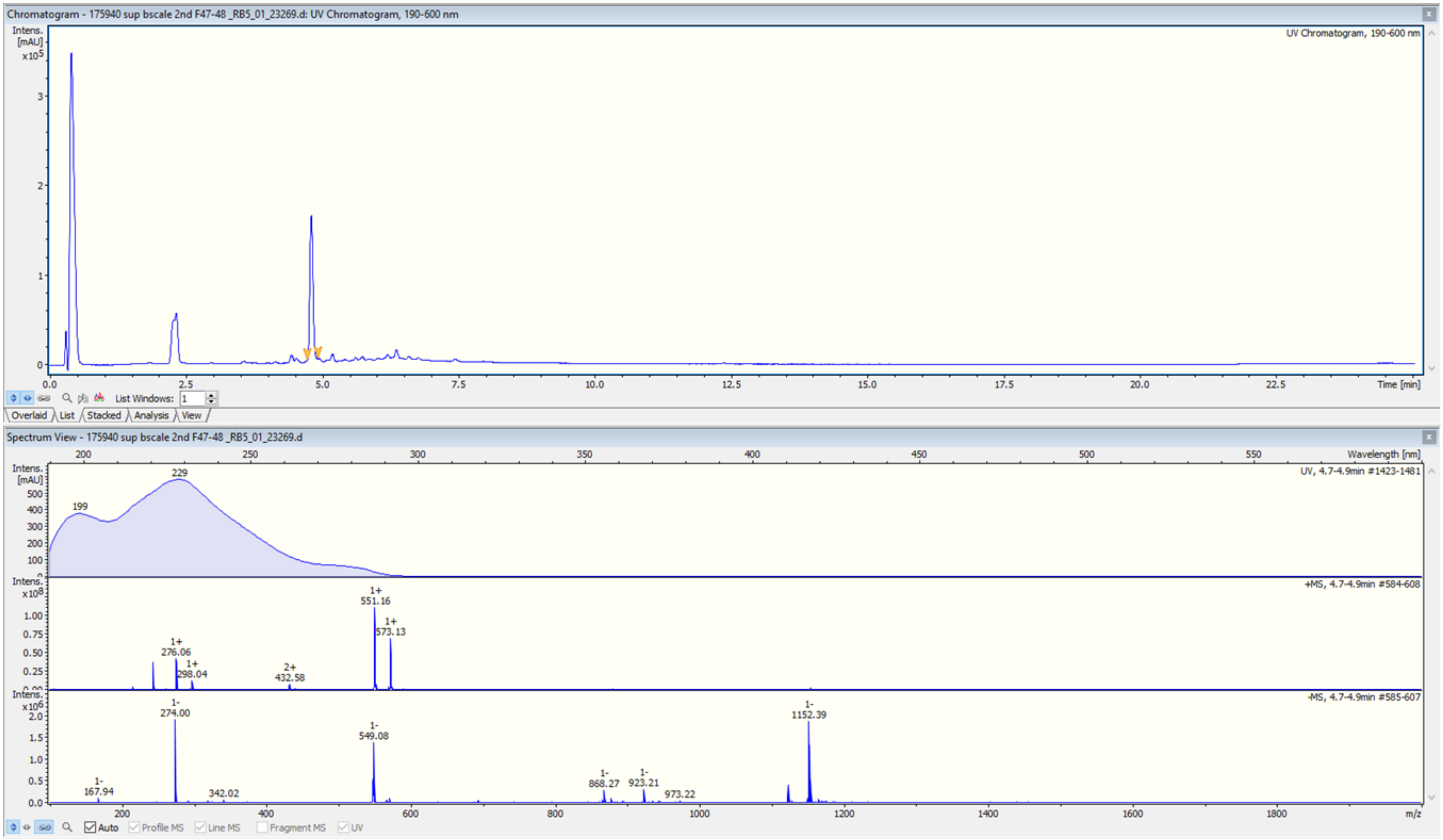

Figure S25: ESIMS data for macrooxazole D (**4**).

1D and 2D NMR data for macrooxazole D (4)

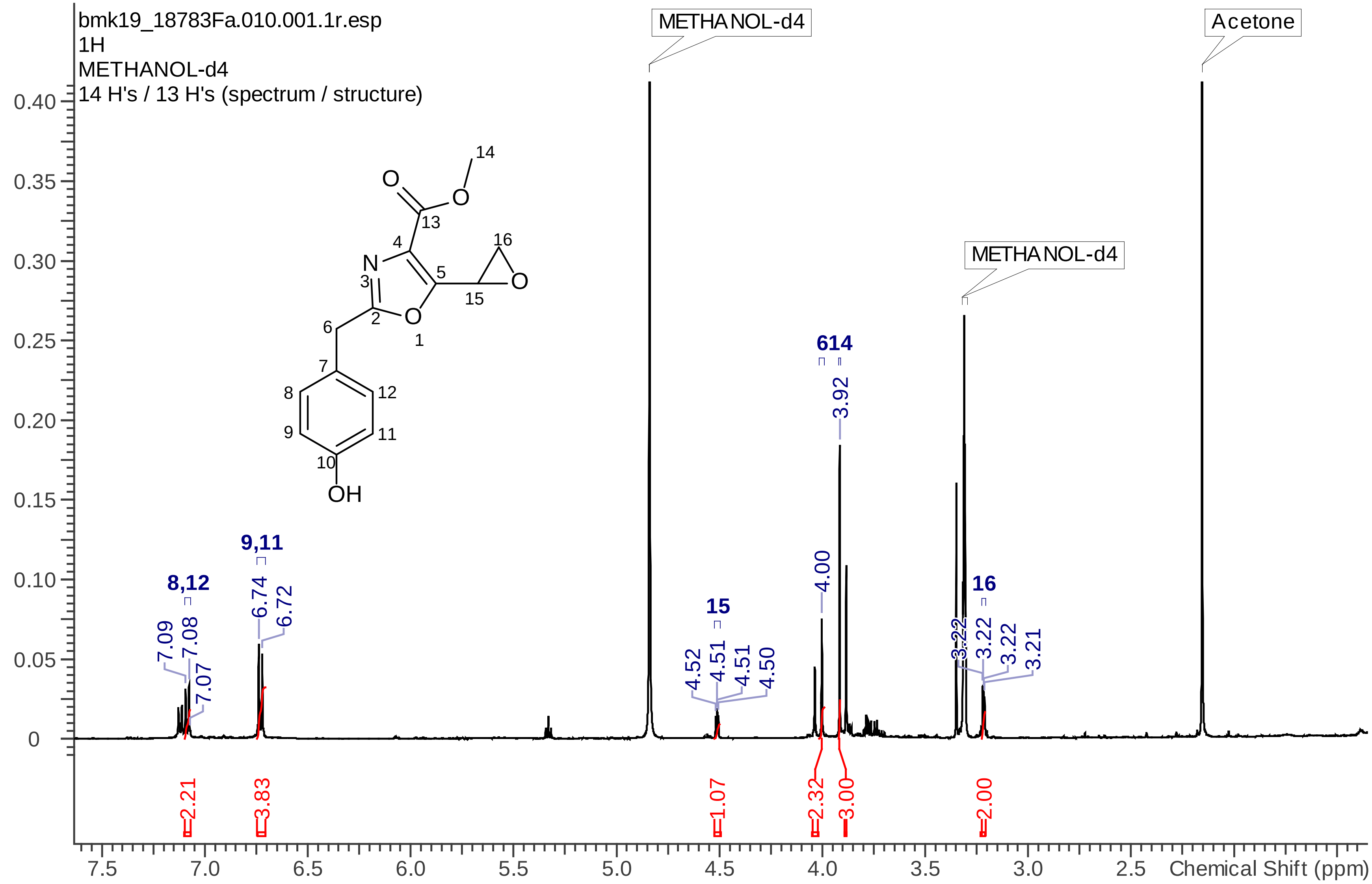

Figure S26: ^{1}H NMR spectrum (MeOH-*d4*, 500 MHz) of macrooxazole D (**4**).

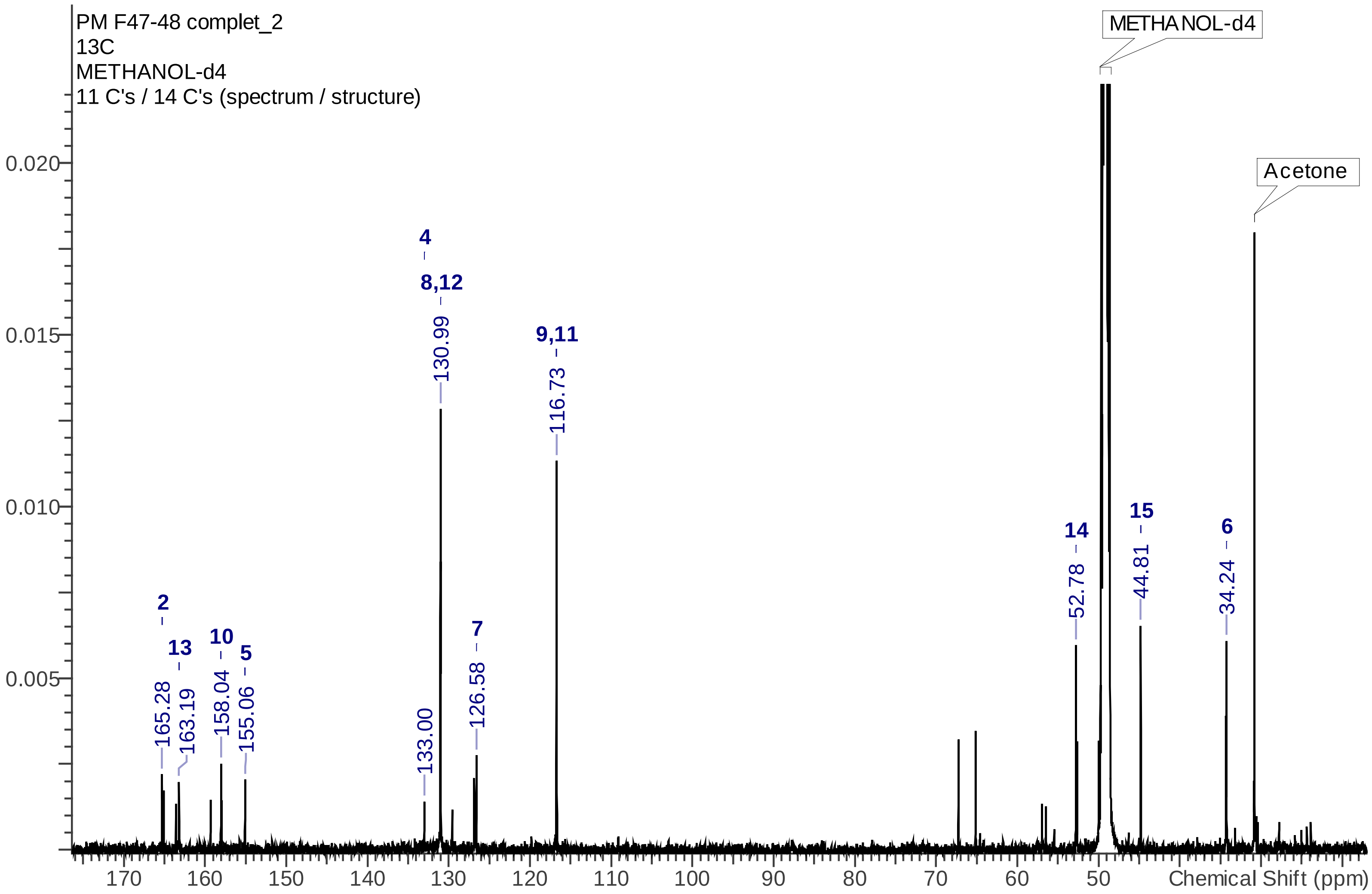

Figure S27: ^{13}C NMR spectrum (MeOH-*d4*, 125 MHz) of macrooxazole D (**4**).

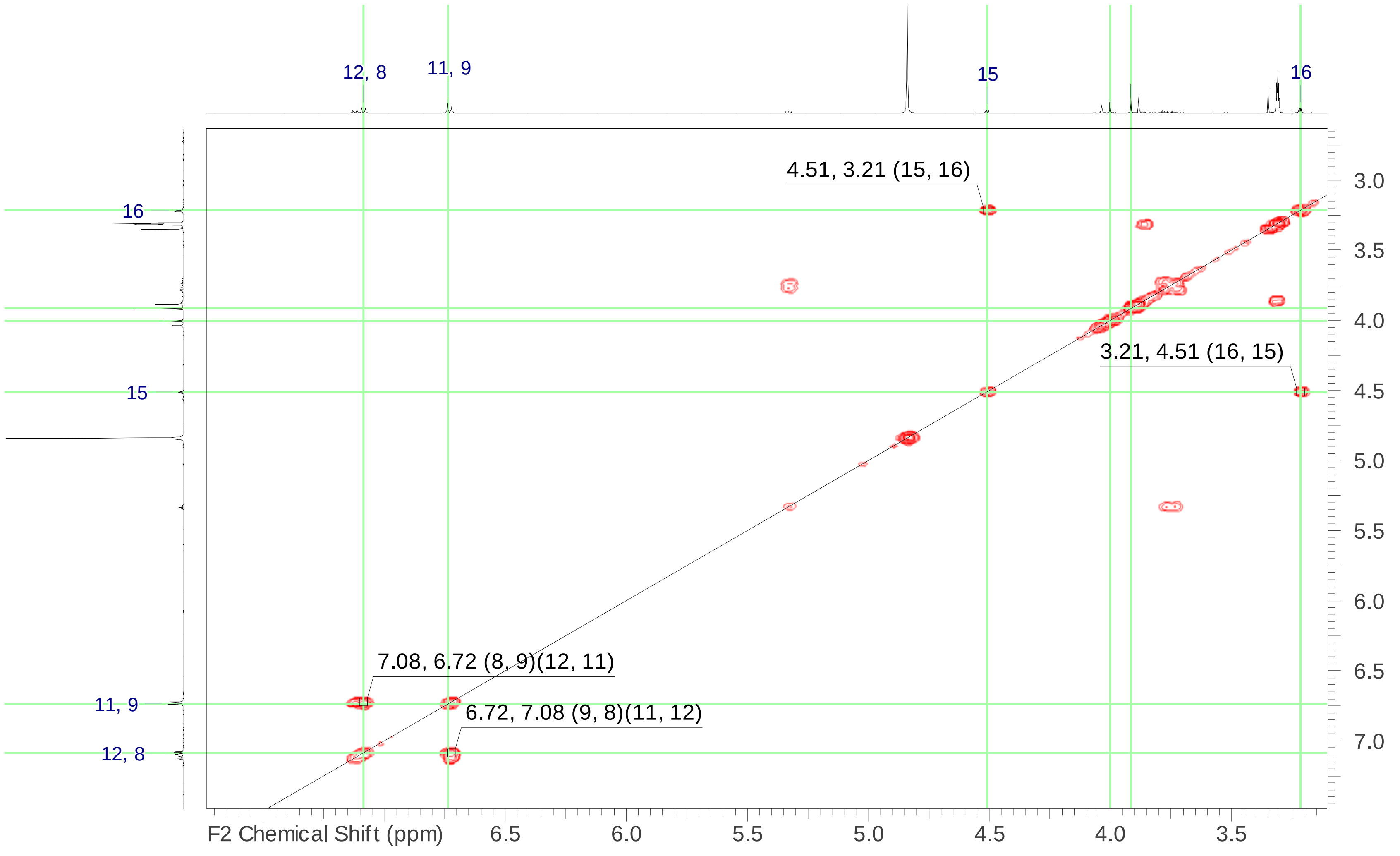

Figure S28: ^{1}H, ^{1}H COSY NMR spectrum (MeOH-*d4*, 500 MHz) of macrooxazole D (**4**).

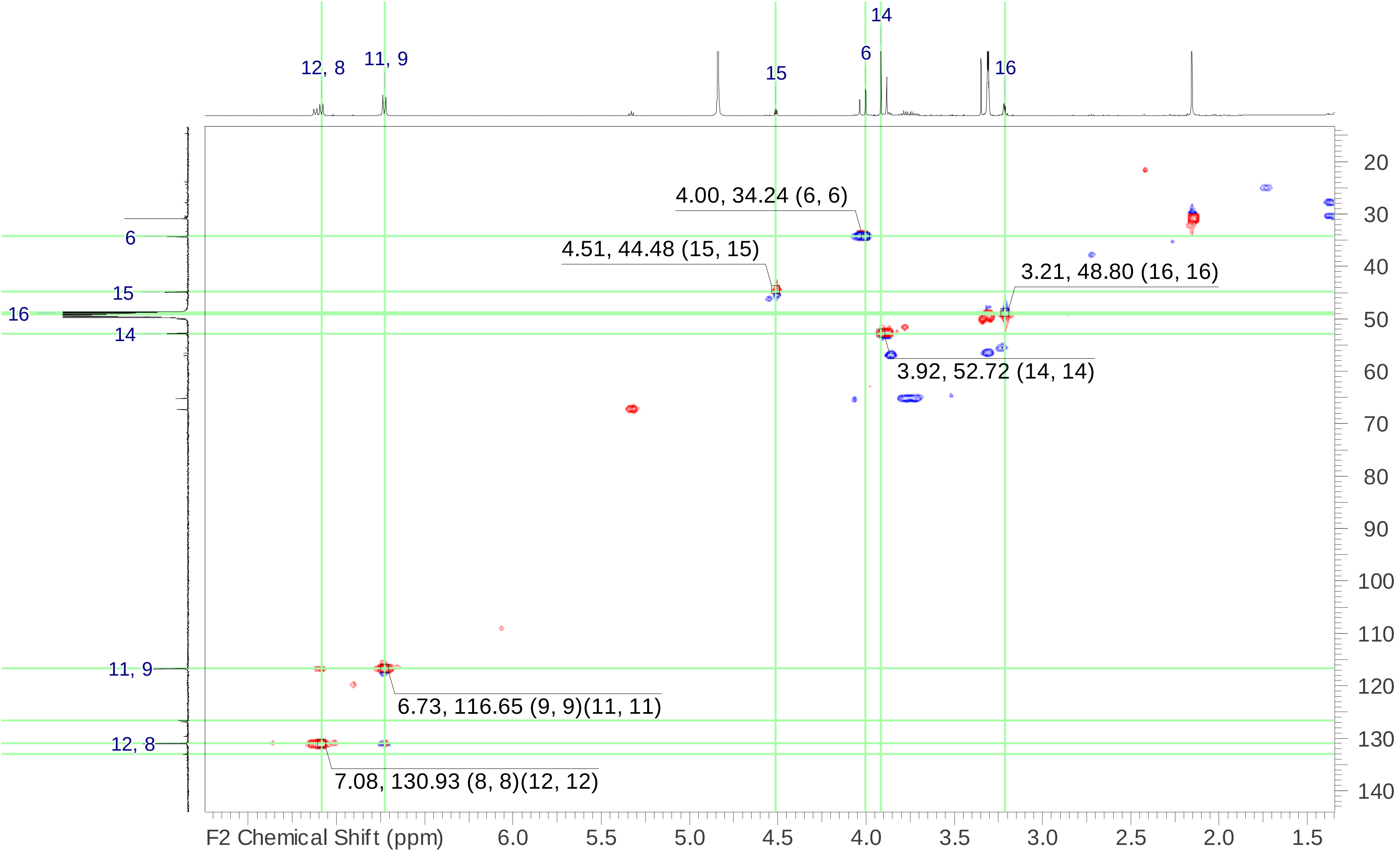

Figure S29: ^{1}H, ^{13}C HSQC NMR spectrum (MeOH-*d4*, 500 MHz) of macrooxazole D (**4**).

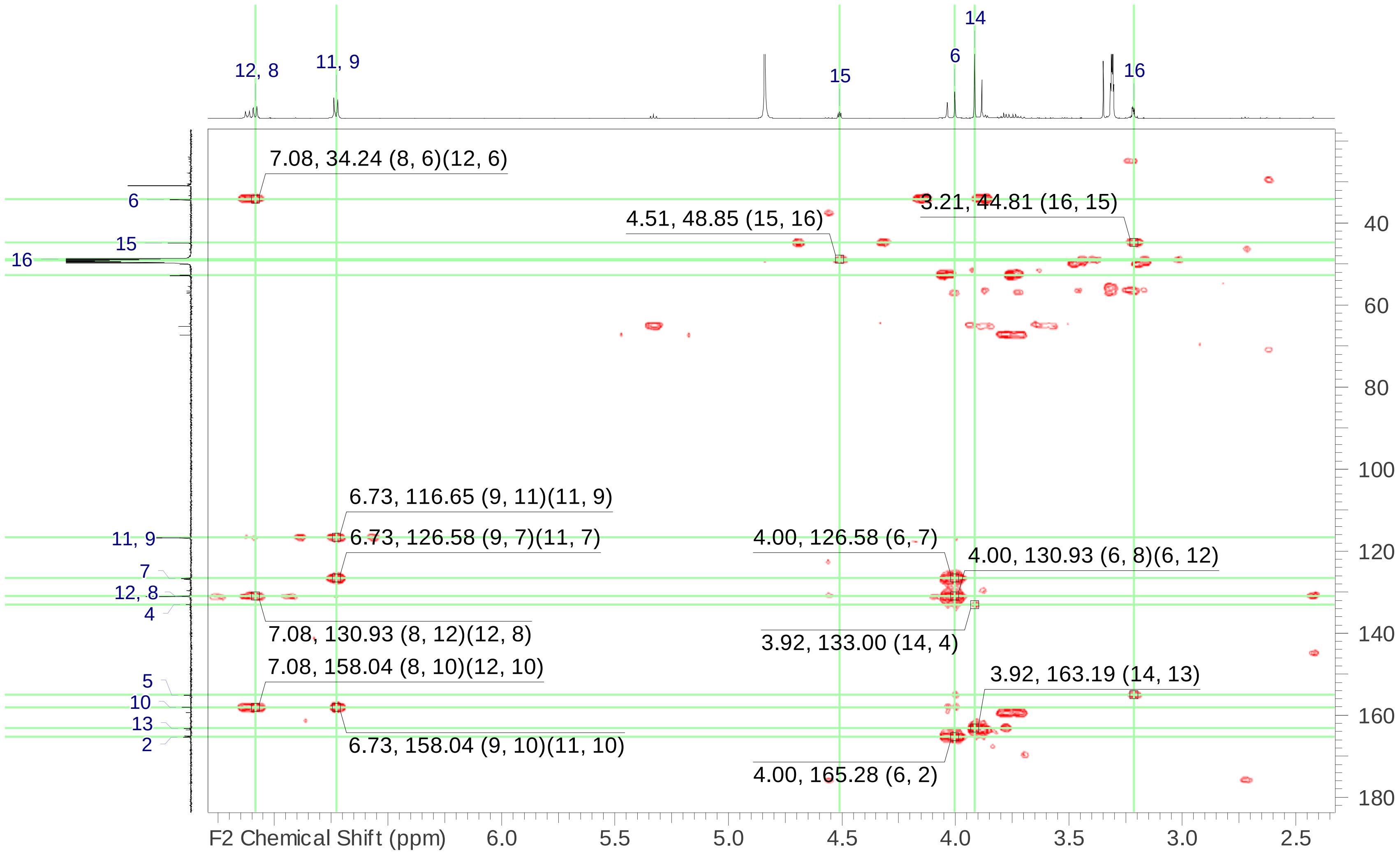

Figure S30: ^{1}H, ^{13}C HMBC NMR spectrum (MeOH-*d4*, 500 MHz) of macrooxazole D (**4**).

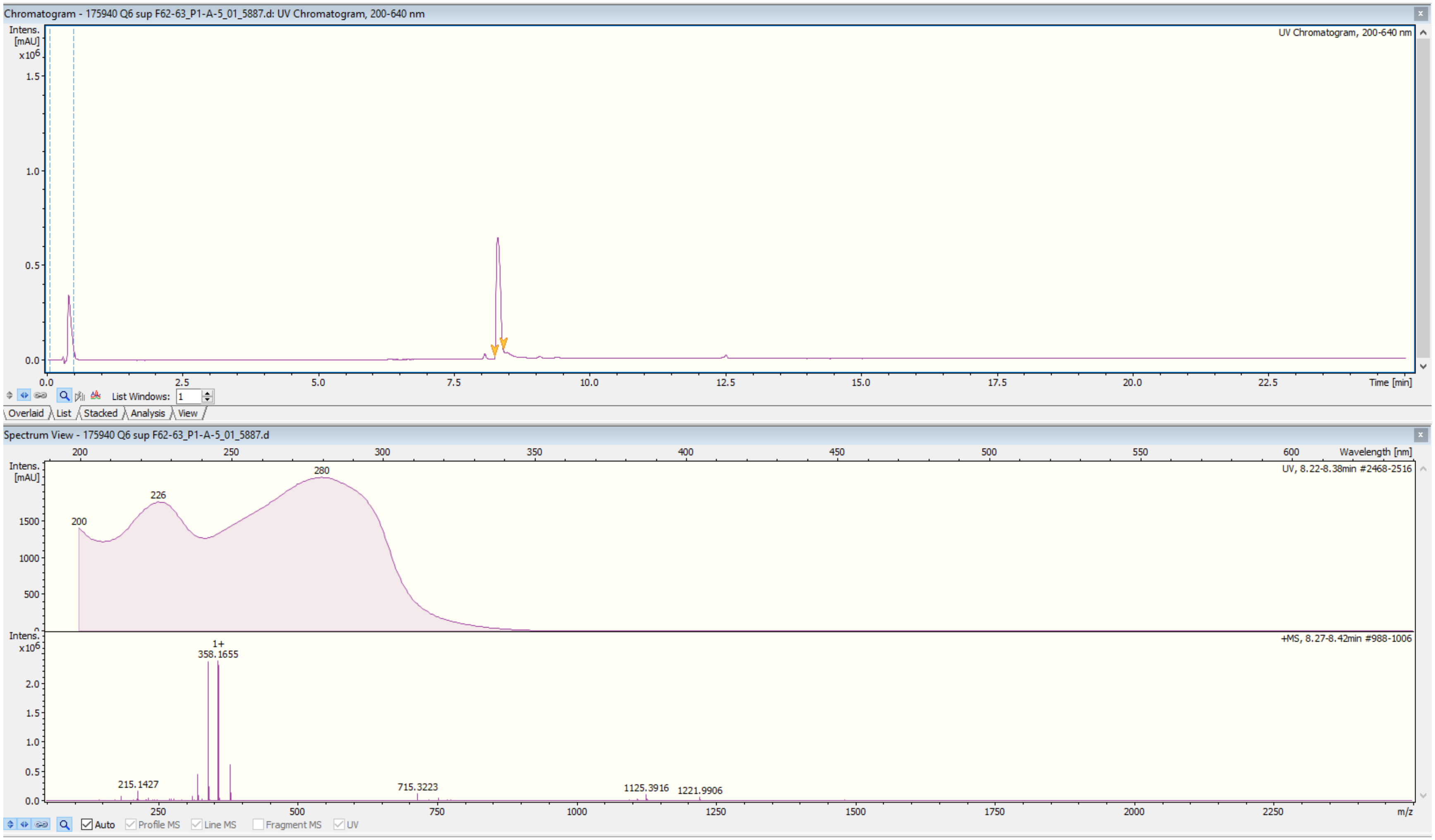

Figure S31: HR-ESIMS data for macrocidin A (**5**).

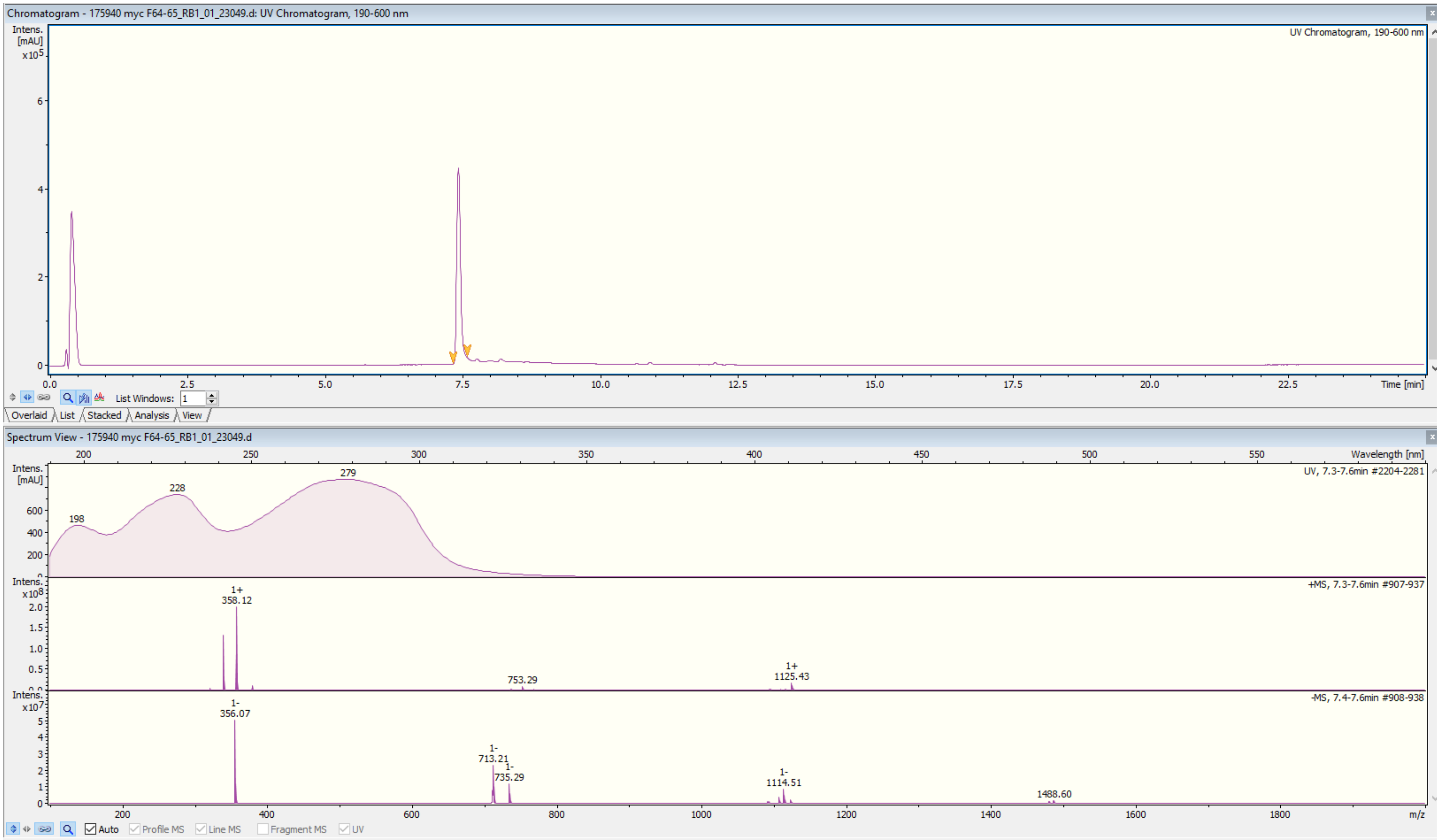

Figure S32: ESIMS data for macrocidin A (**5**).

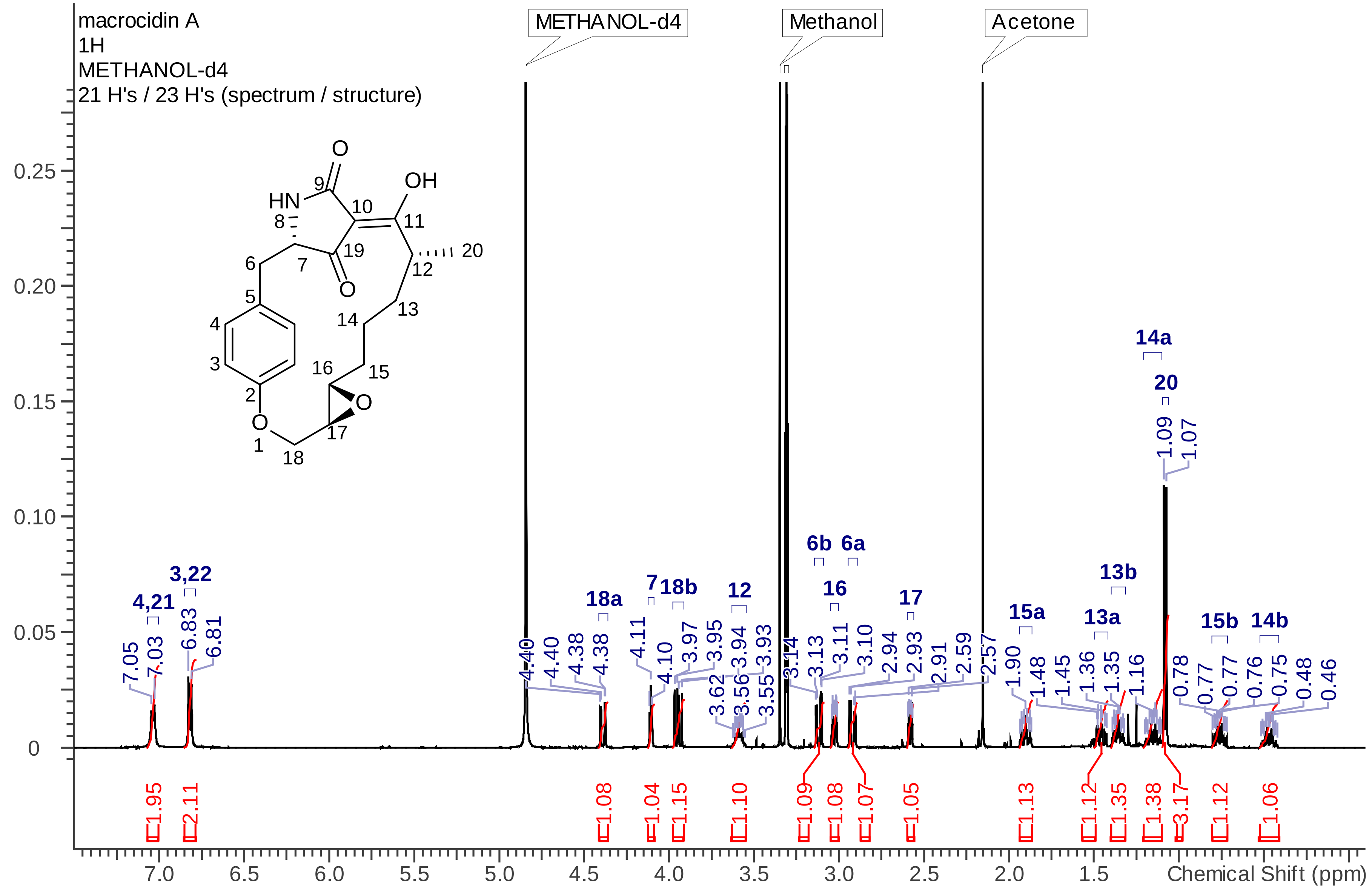

Figure S33: ^{1}H NMR spectrum (MeOH-*d4*, 500 MHz) of macrocidin A (**5**)

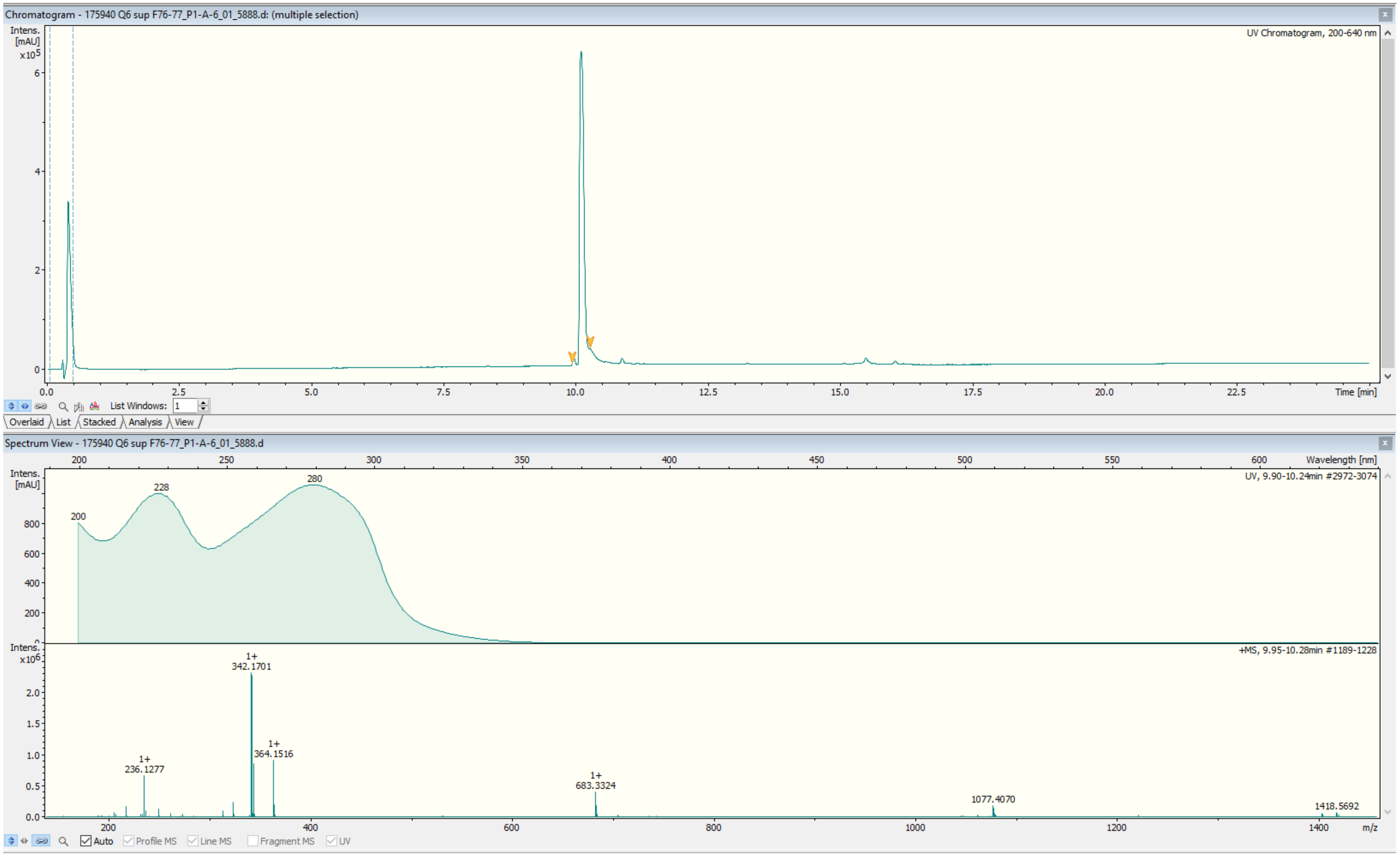

Figure S34: HR-ESIMS data for macrocidin Z (**6**).

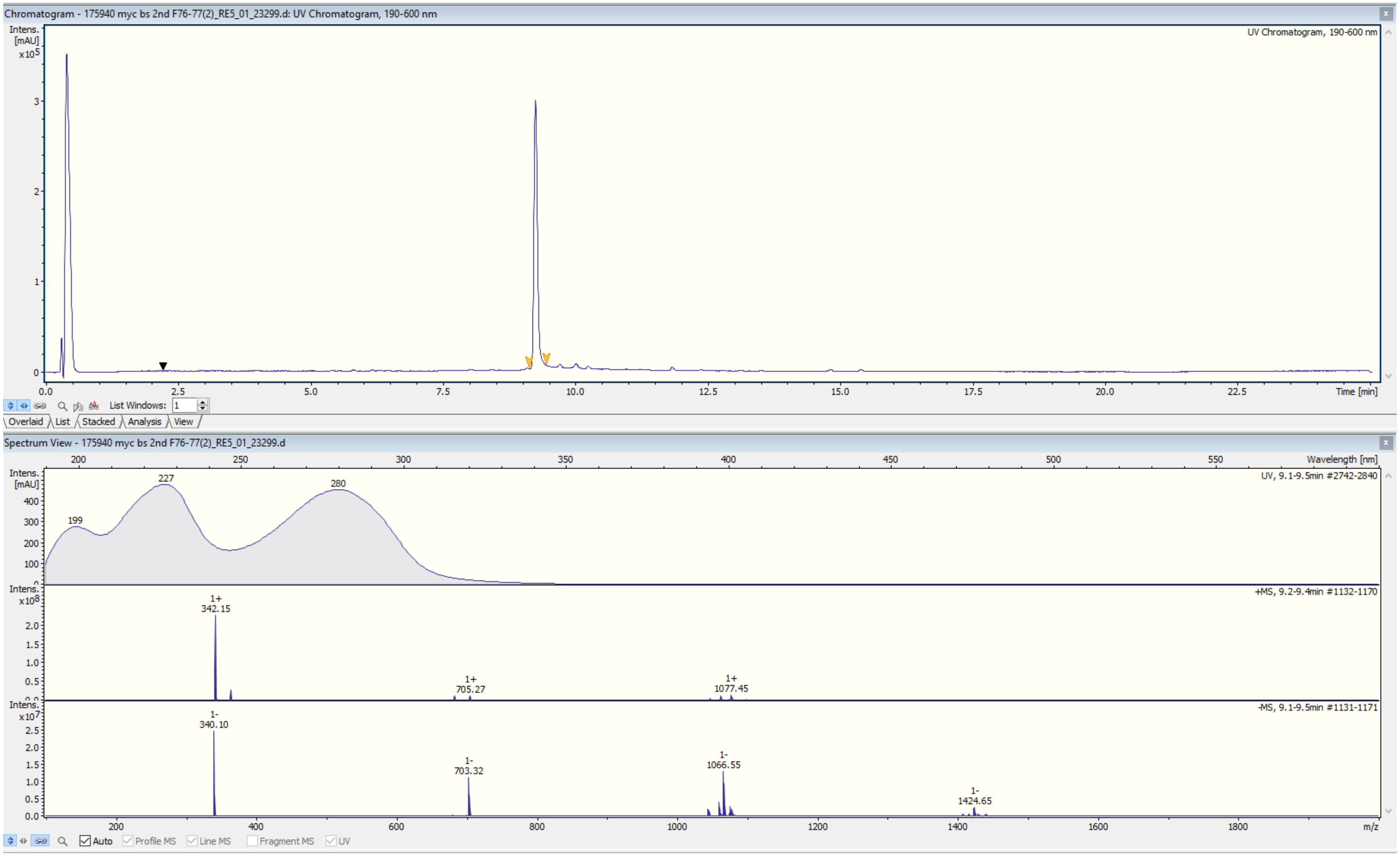

Figure S35: ESIMS data for macrocidin Z (**6**).

1D and 2D NMR data for macrocidin Z (6)

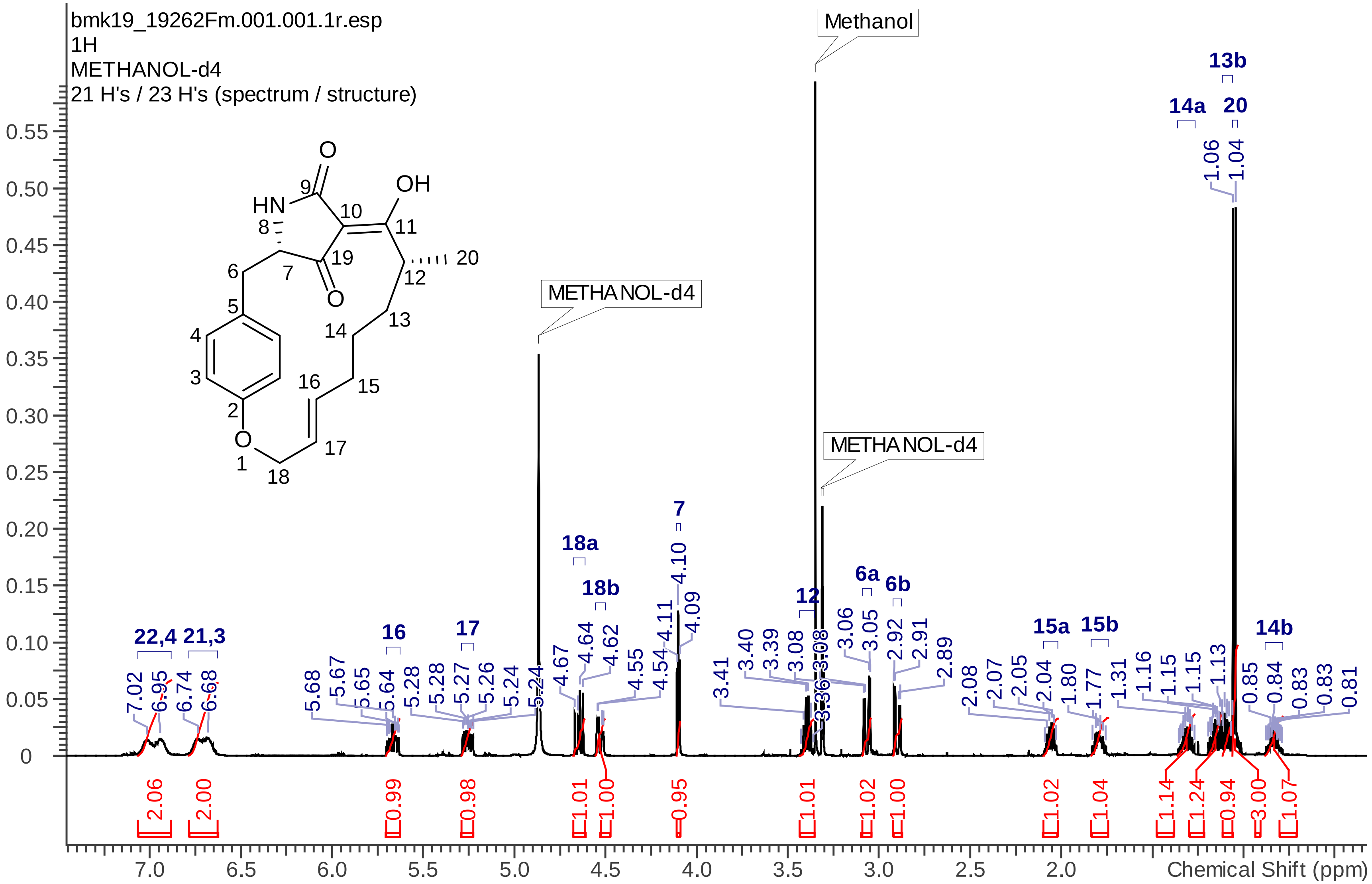

Figure S36: ^{1}H NMR spectrum (MeOH-*d4*, 500 MHz) of macrocidin Z (**6**).

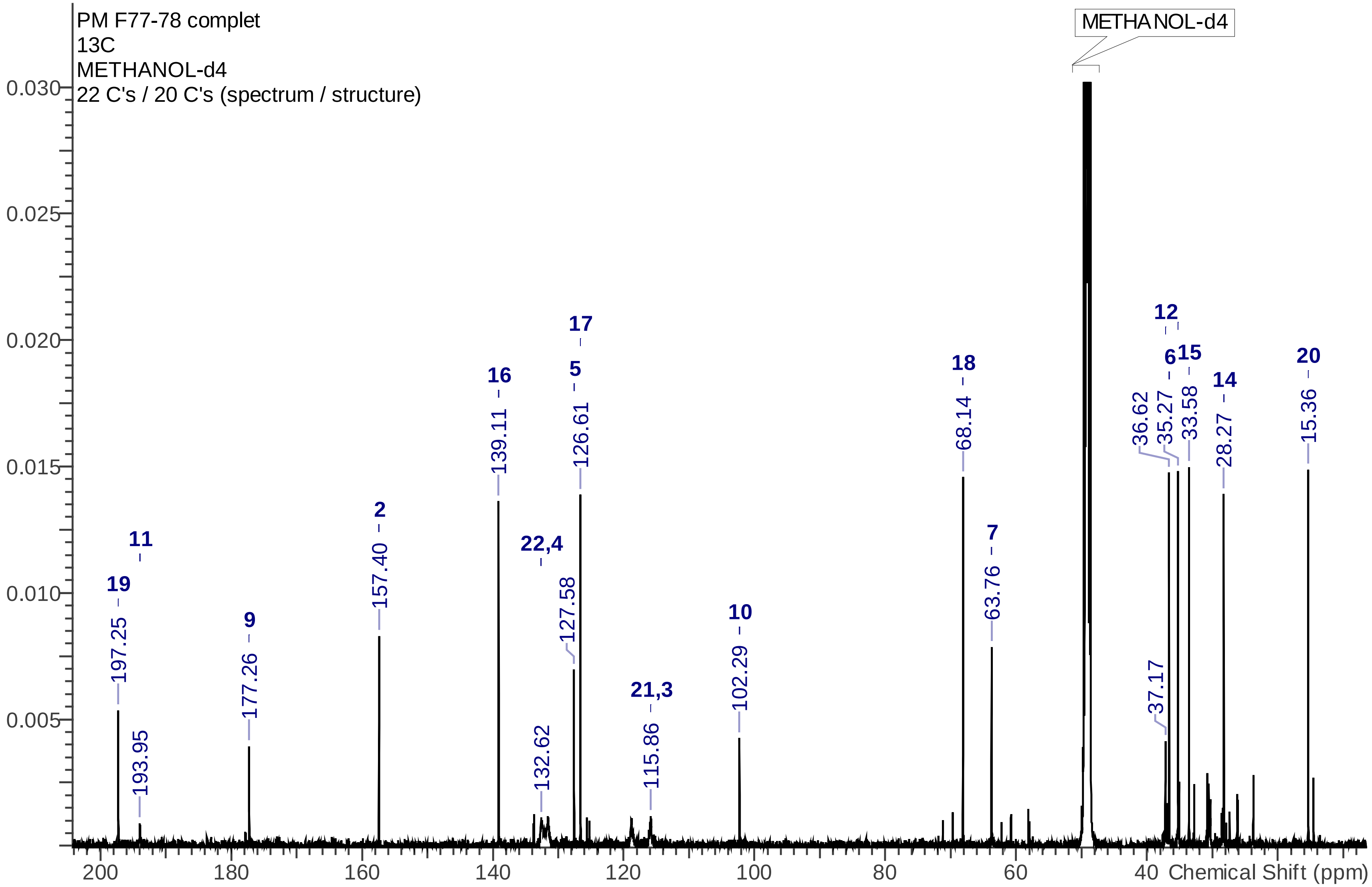

Figure S37: ^{13}C NMR spectrum (MeOH-*d4*, 125 MHz) of macrocidin Z (**6**).

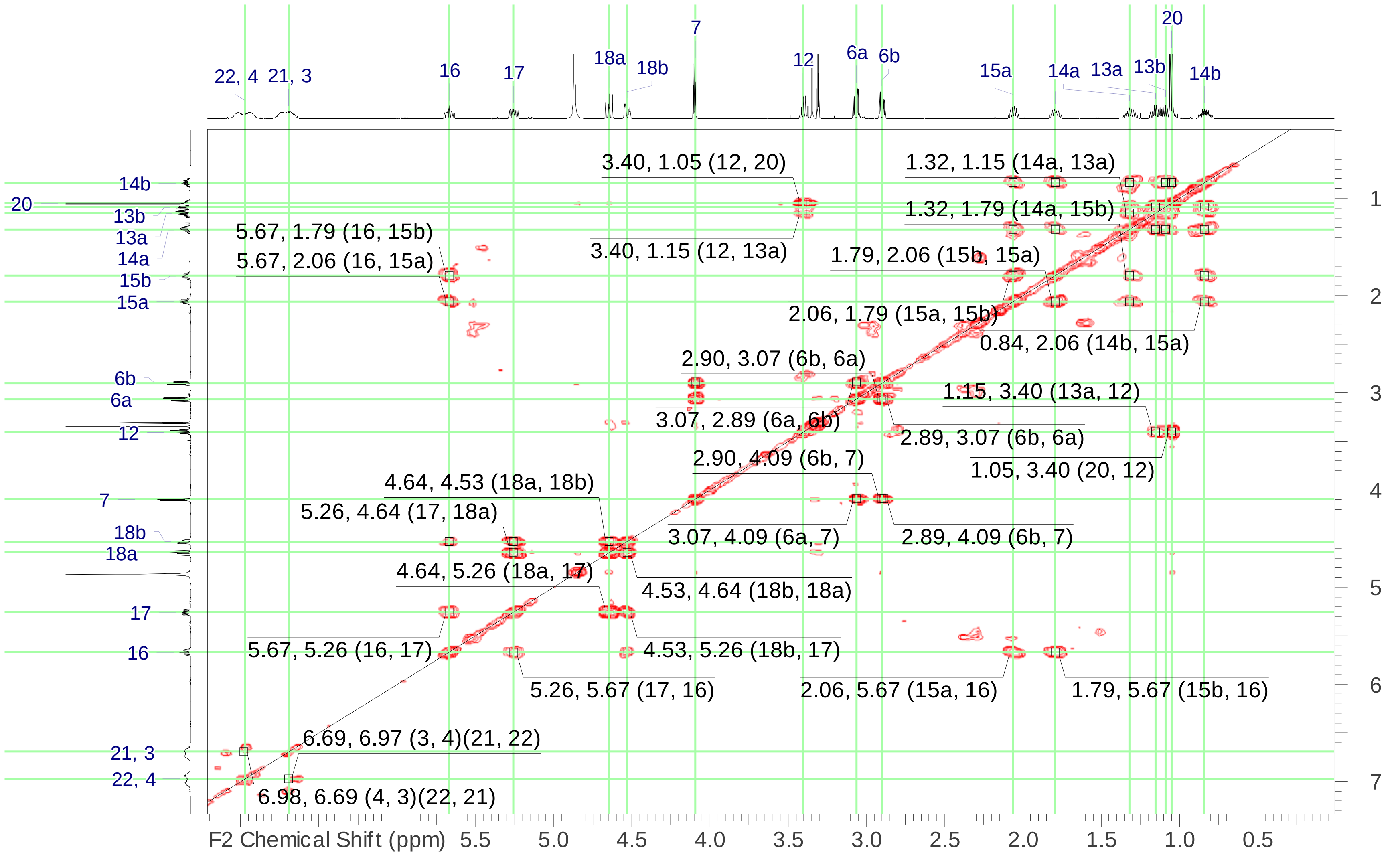

Figure S38: ^{1}H, ^{1}H COSY NMR spectrum (MeOH-*d4*, 500 MHz) of macrocidin Z (**6**).

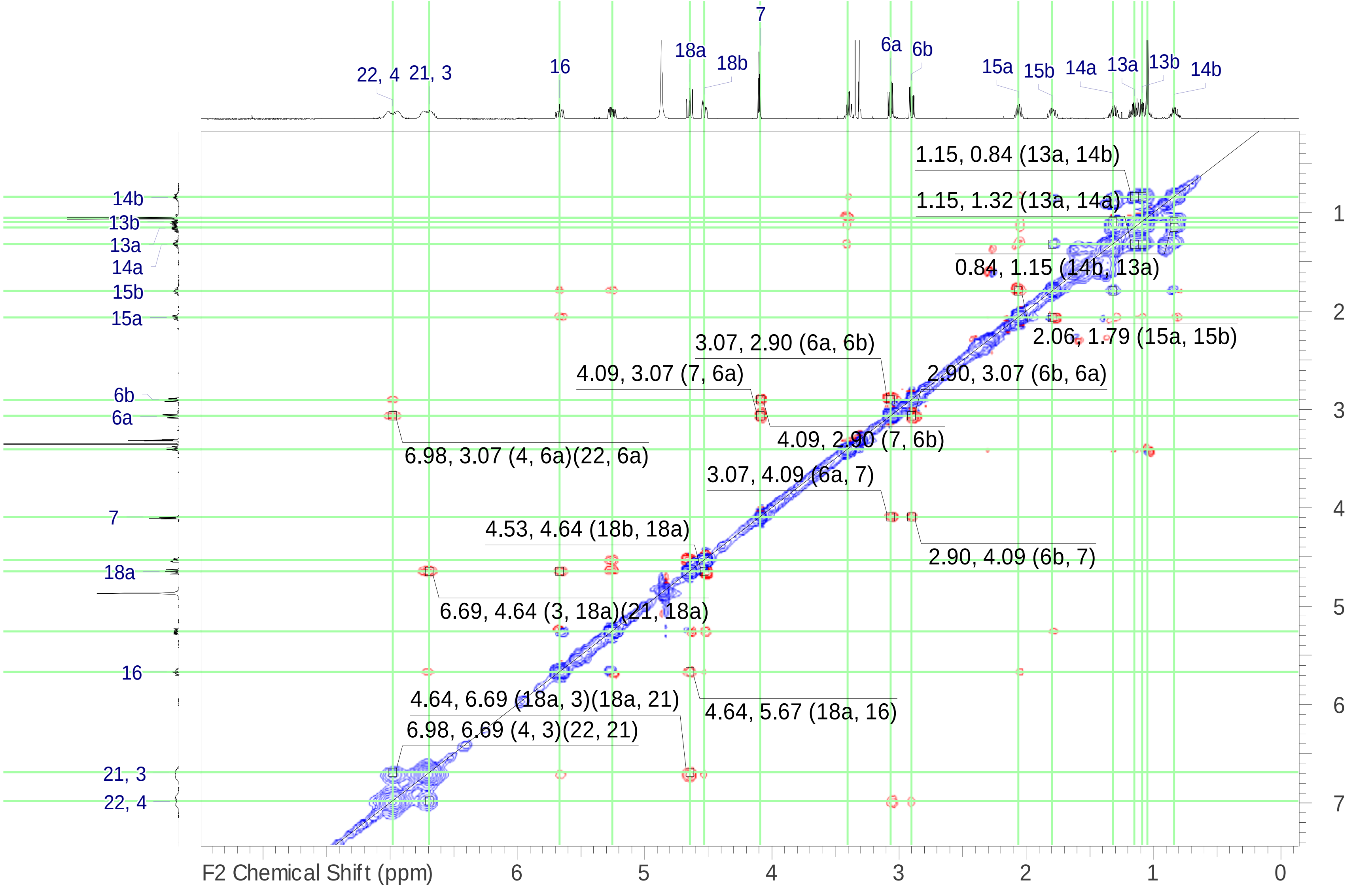

Figure S39: ^{1}H, ^{1}H ROESY NMR spectrum (MeOH-*d4*, 500 MHz) of macrocidin Z (**6**)

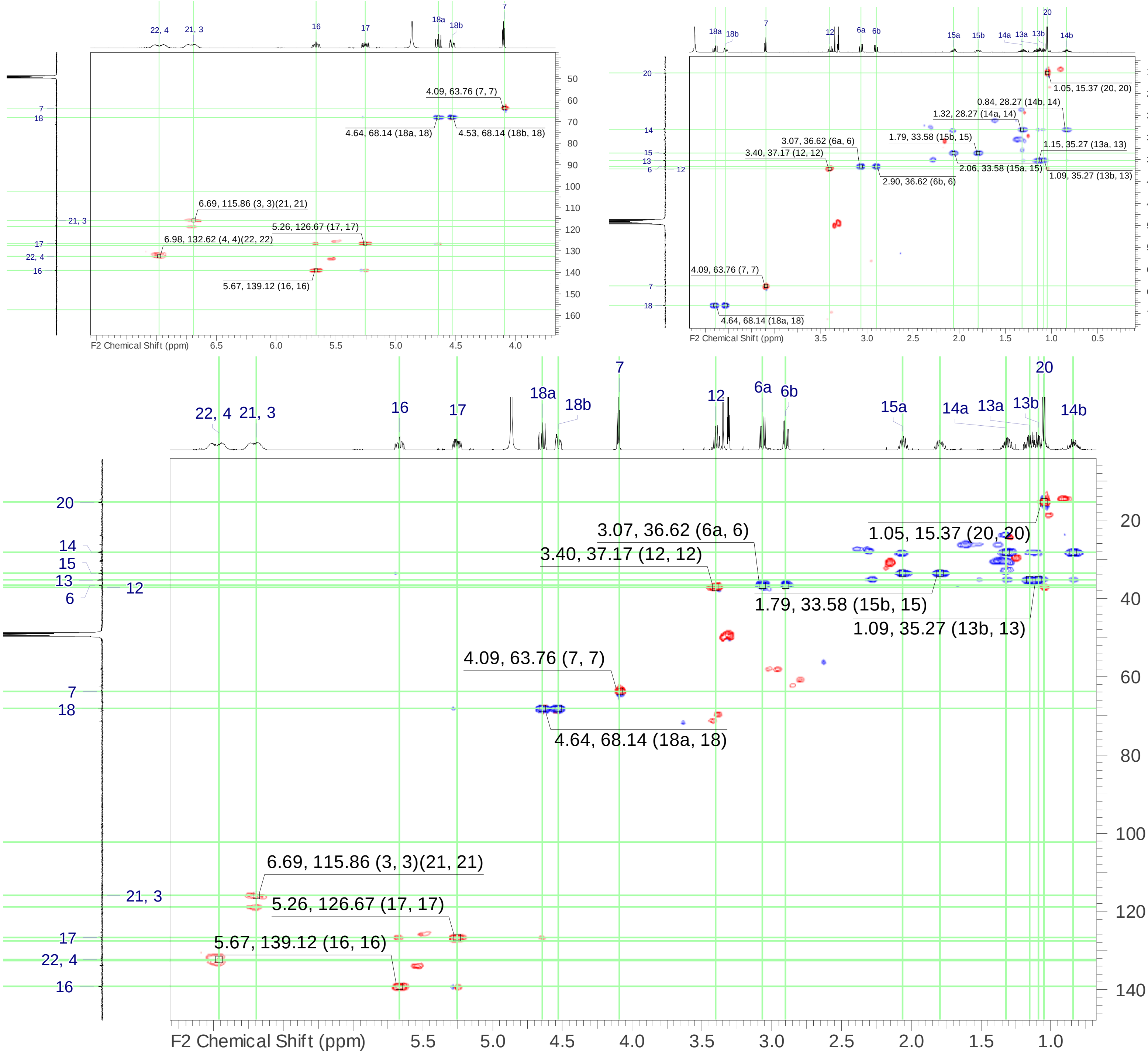

Figure S40: ^{1}H, ^{13}C HSQC NMR spectrum (MeOH-*d4*, 500 MHz) of macrocidin Z (**6**).

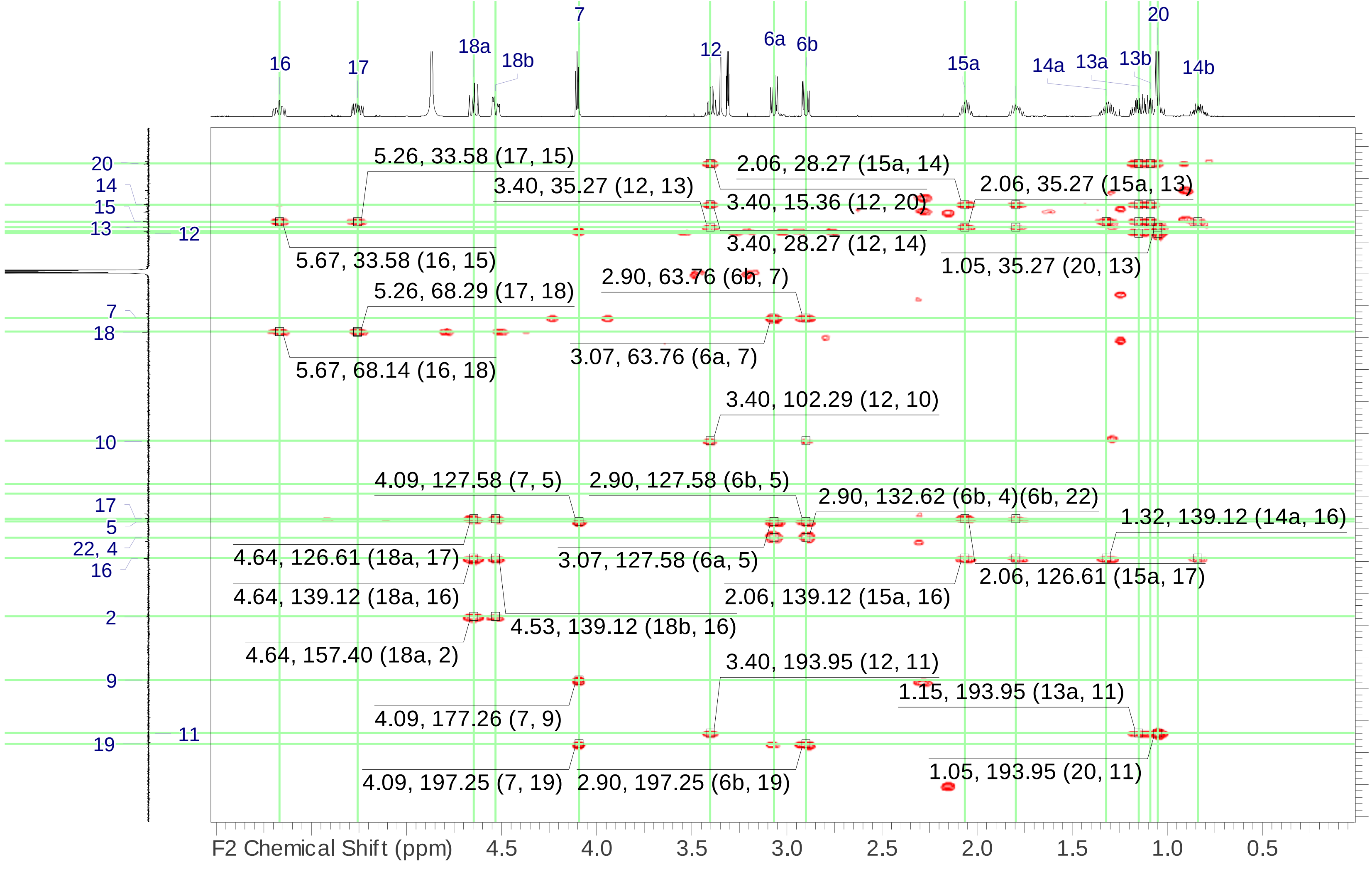

Figure S41: ^{1}H, ^{13}C HMBC NMR spectrum (MeOH-*d4*, 500 MHz) of macrocidin Z (**6**)

Table S1: Minimum Inhibitory concentrations (MIC) of compounds **1-6** against tested microorganisms.

**Test organisms**	**MIC (µg/mL)**						
	**1**	**2**	**3**	**2/4** (ratio1:2)	**5**	**6**	**References**
Schizosaccharomyces pombe DSM70572	n.i	n.i	n.i	n.i	n.i	n.i	33.3[n]
Pichia anomala DSM6766	n.i	n.i	n.i	n.i	n.i	n.i	33.3[n]
Mucor hiemalis DSM2656	n.i	n.i	66.7	n.i	n.i	n.i	66.7[n]
Candida albicans DSM1665	n.i	n.i	n.i	n.i	n.i	n.i	66.7[n]
Rhodoturula glutinis DSM10134	n.i	n.i	n.i	n.i	n.i	n.i	8.3[n]
Micrococcus luteus DSM1790	n.i	n.i	n.i	n.i	n.i	66.7	0.4[o]
Escherichia coli DSM1116	n.i	n.i	n.i	n.i	n.i	n.i	1.7[o]
Bacillus subtilis DSM10	n.i	n.i	66.7	n.i	16.7	n.i	16.7[o]
Mycolicibacterium smegmatis ATCC700084	n.i	n.i	n.i	n.i	33.3	n.i	1.7[k]
Staphylococcus aureus DSM346	n.i	n.i	n.i	n.i	n.i	n.i	0.2[o]
Pseudomonas aeruginosa PA14	n.i	n.i	n.i	n.i	n.i	n.i	0.4[g]
Chromobacterium violaceum DSM30191	n.i	n.i	n.i	n.i	n.i	n.i	0.4[o]

n.i.: No inhibition observed under test conditions, g Gentamycin 1 mg/mL, k Kanamycin 1 mg/mL, n Nystatin 1 mg/mL, o Oxytetracyclin 1 mg/mL. Starting concentration for antimicrobial assay were 66.7 µg/mL.

Table S2: Cytotoxic effect (IC_{50}) of compound **1-6** against two cancer cell lines.

**Cell lines**	**IC_{50} (µg/mL)**						
	**1**	**2**	**3**	**2/4** (ratio 1:2)	**5**	**6**	**Epothilone B**
**KB3.1**	n.a	n.a	n.a	23	s.i	s.i	0.000016
**L929**	n.a	n.a	n.a	23	n.a	n.a	0.00026

n.a: Not active; s.i: slight inhibition of cells proliferation; Epothilone B 1mg/mL. Starting concentration for cytotoxicity assay were 37 µg/mL.

NMR spectra of the synthetic intermediates

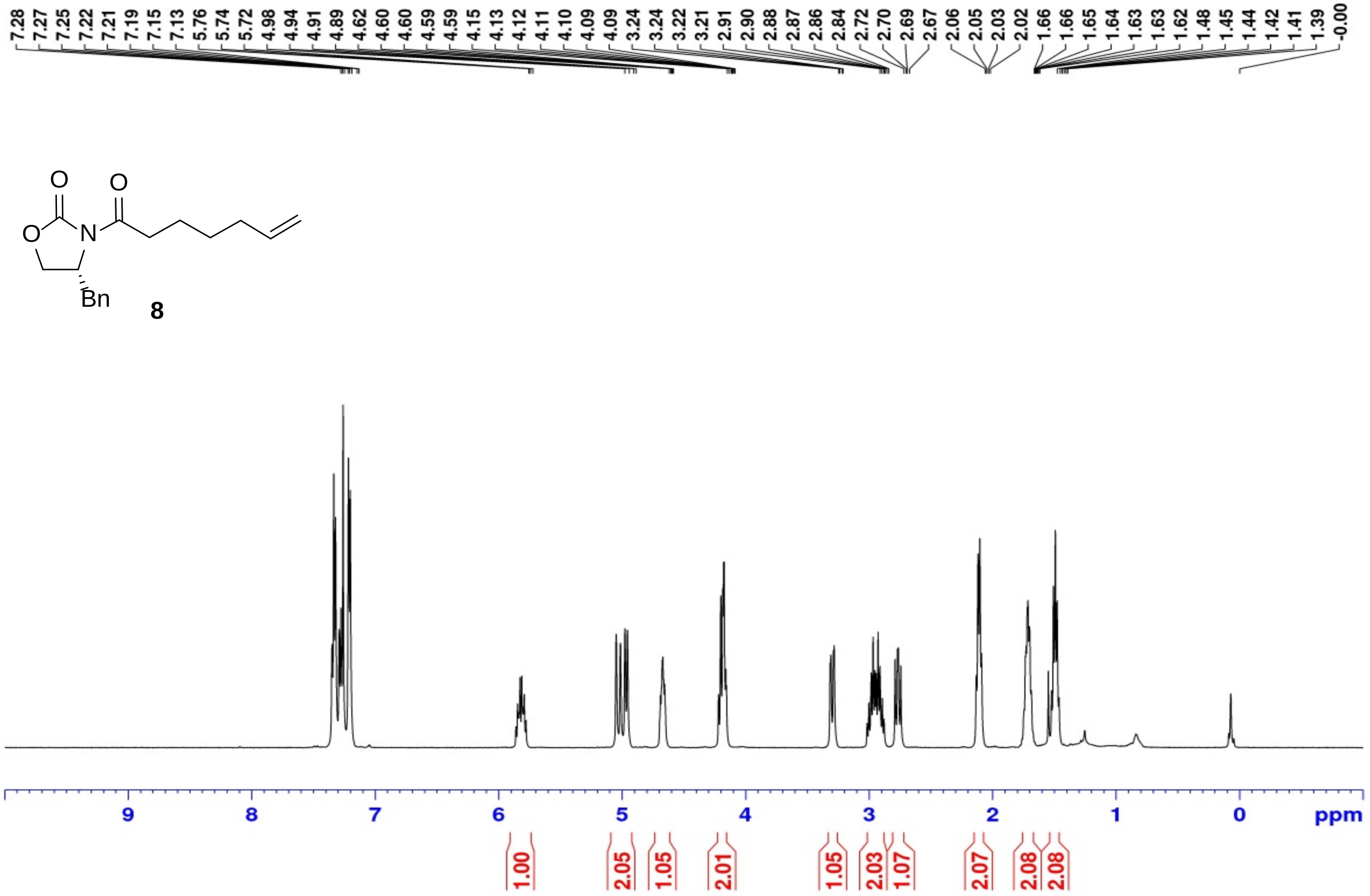

Figure S42: ^{1}H-NMR spectrum of compound **8** in $CDCl_3$.

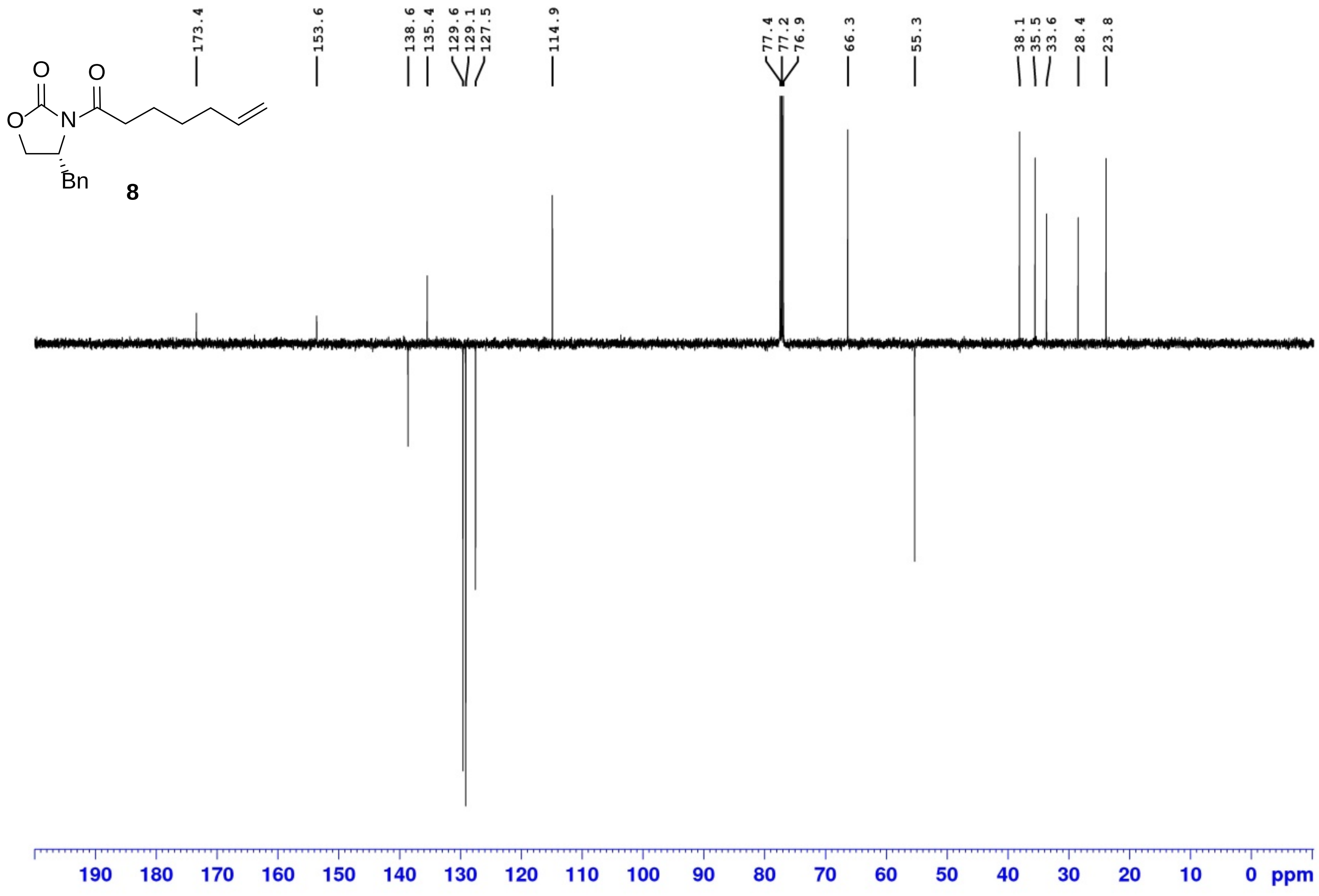

Figure S43: ^{13}C-NMR spectrum of compound **8** in $CDCl_3$.

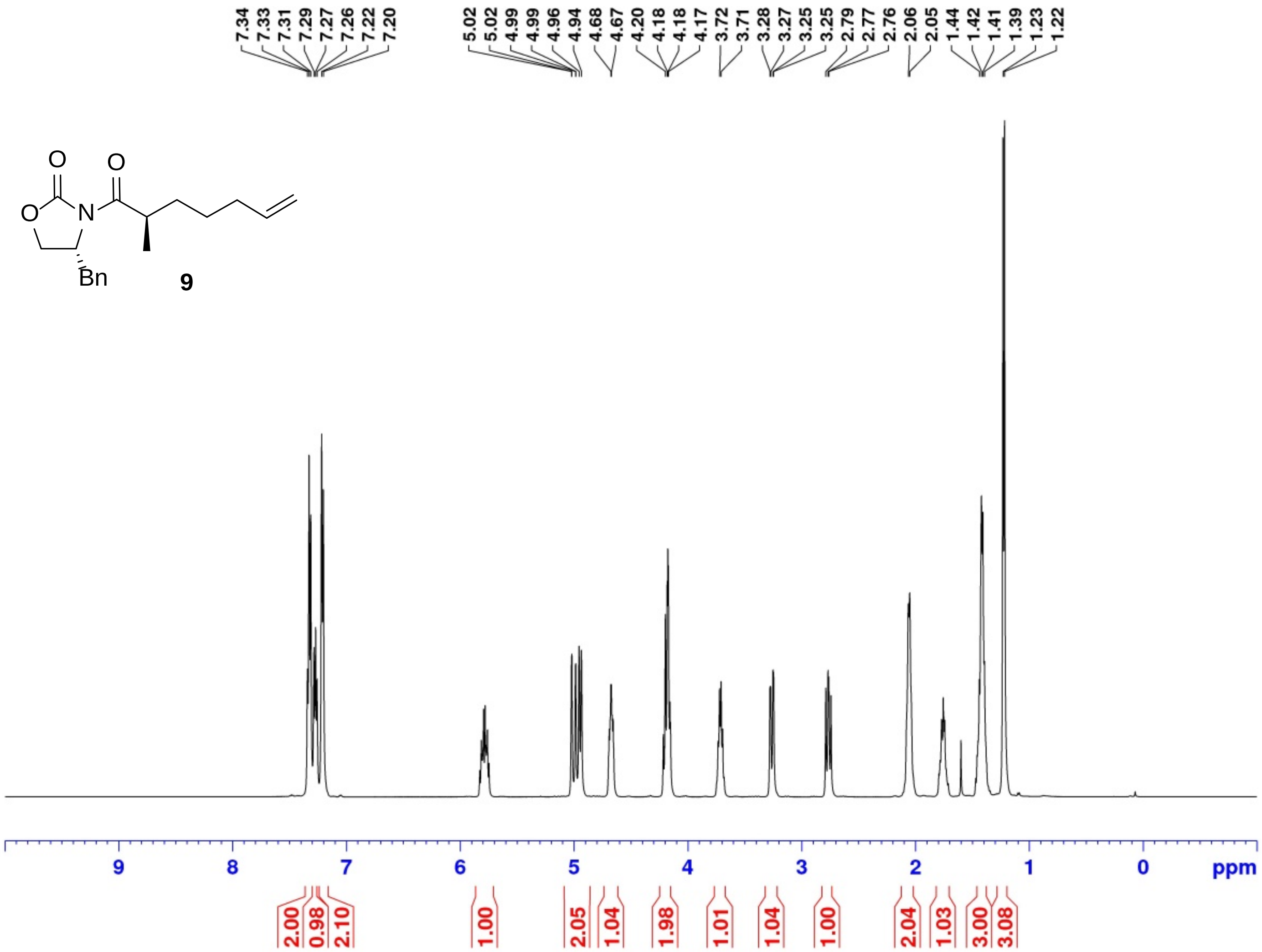

Figure S44: ^{1}H-NMR spectrum of compound **9** in $CDCl_3$.

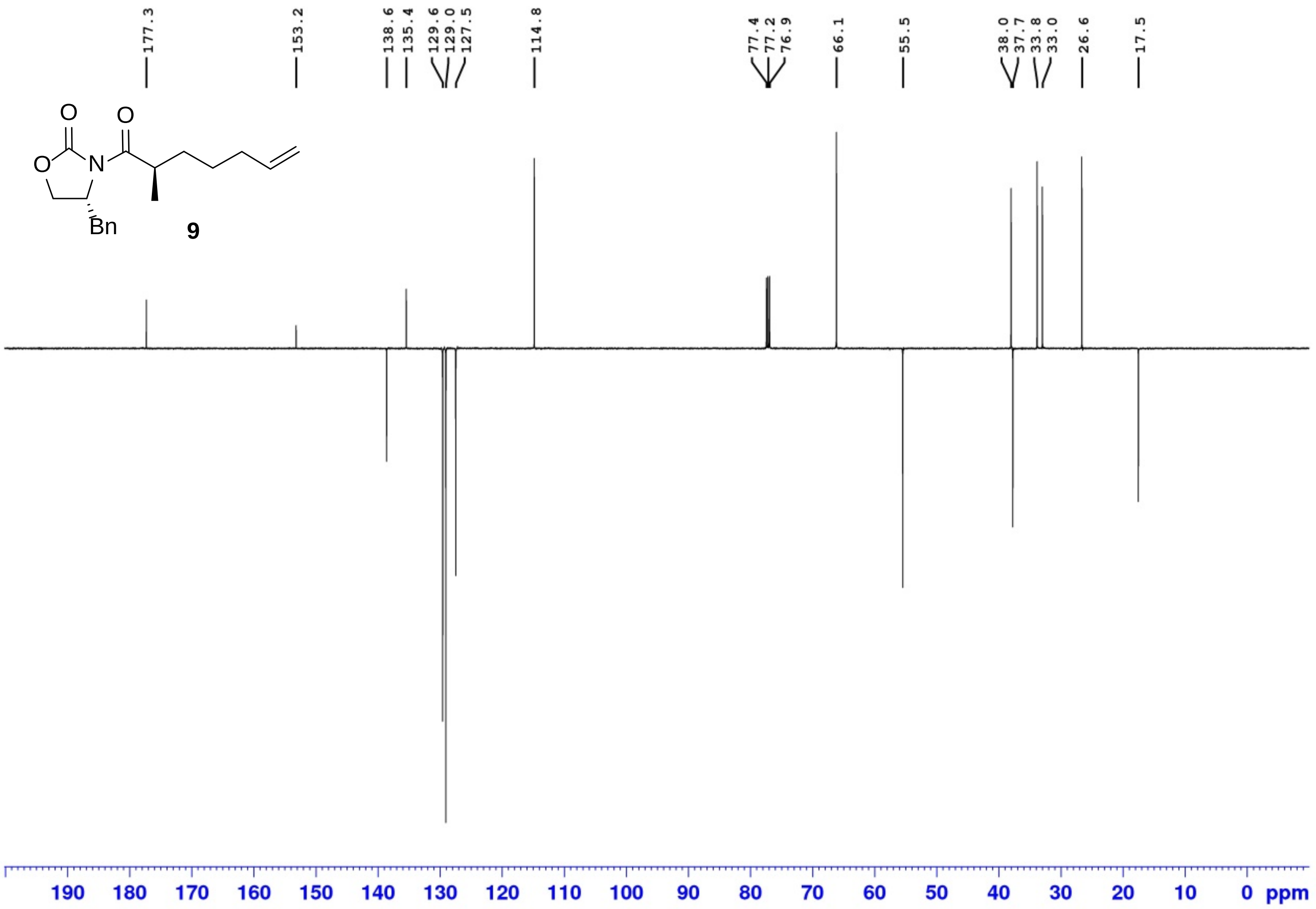

Figure S45: ^{13}C-NMR spectrum of compound **9** in $CDCl_3$.

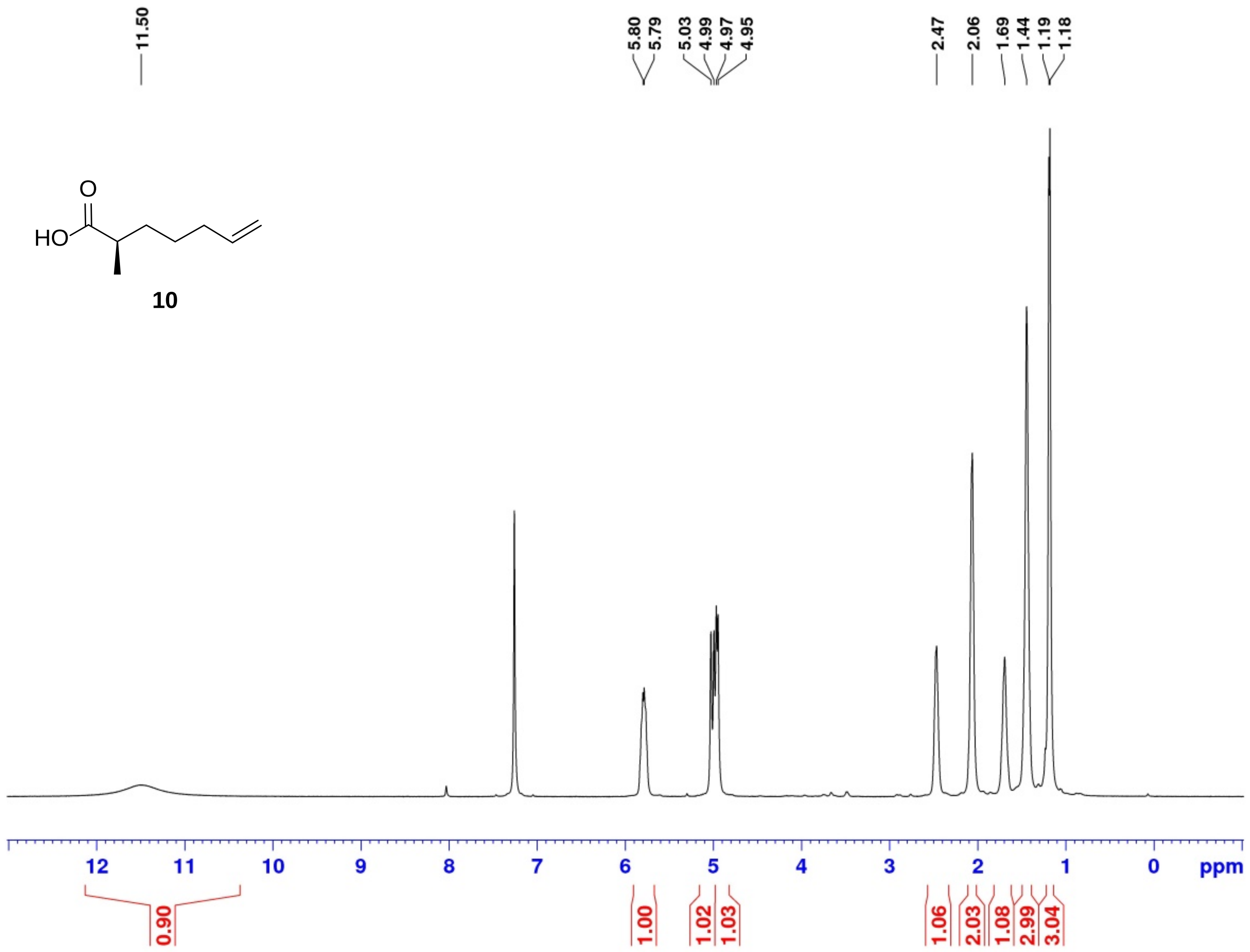

10

Figure S46: ^{1}H-NMR spectrum of compound **10** in $CDCl_3$.

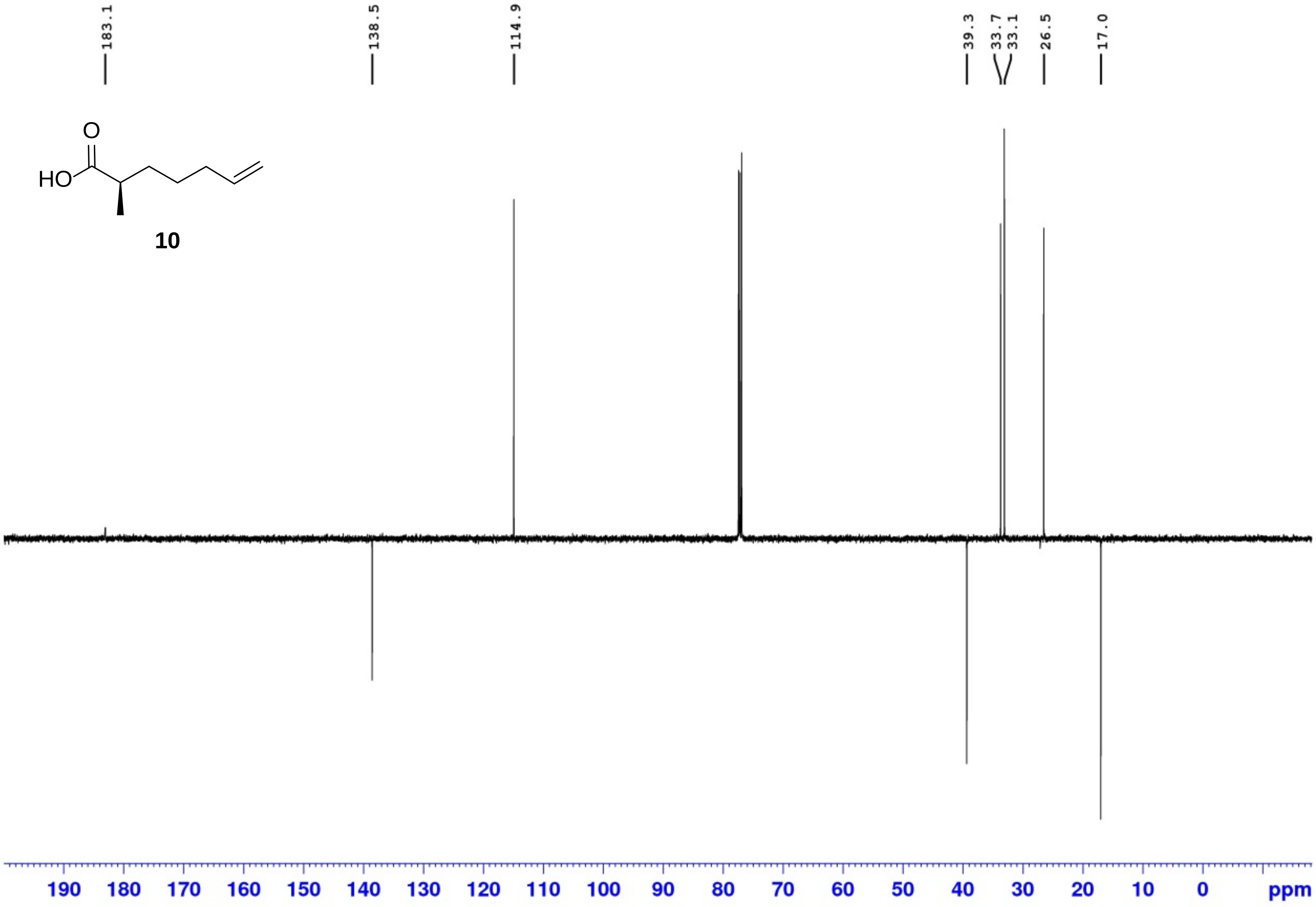

Figure S47: ^{13}C-NMR spectrum of compound **10** in $CDCl_3$.

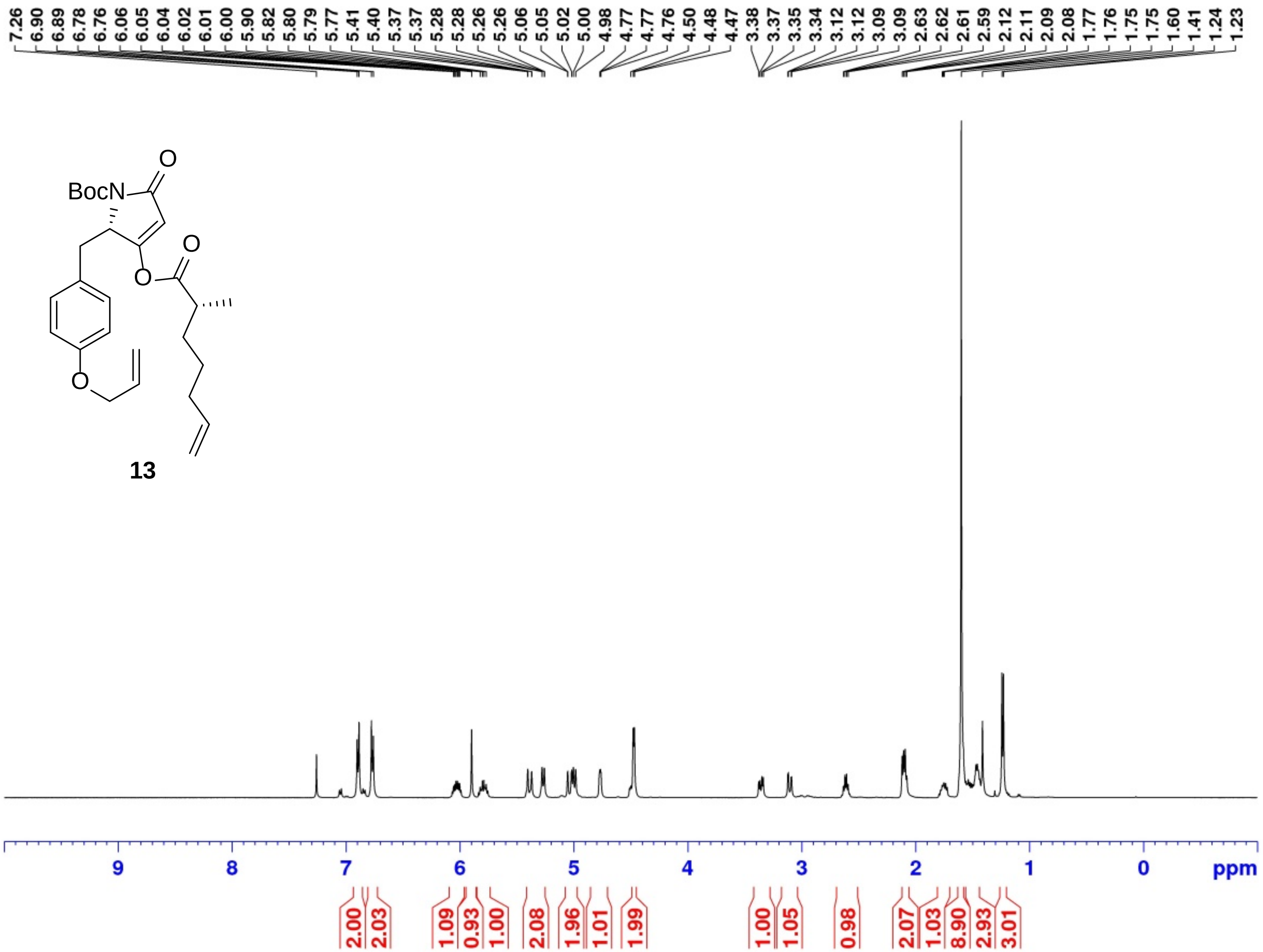

Figure S48: ^{1}H-NMR spectrum of compound **13** in $CDCl_3$.

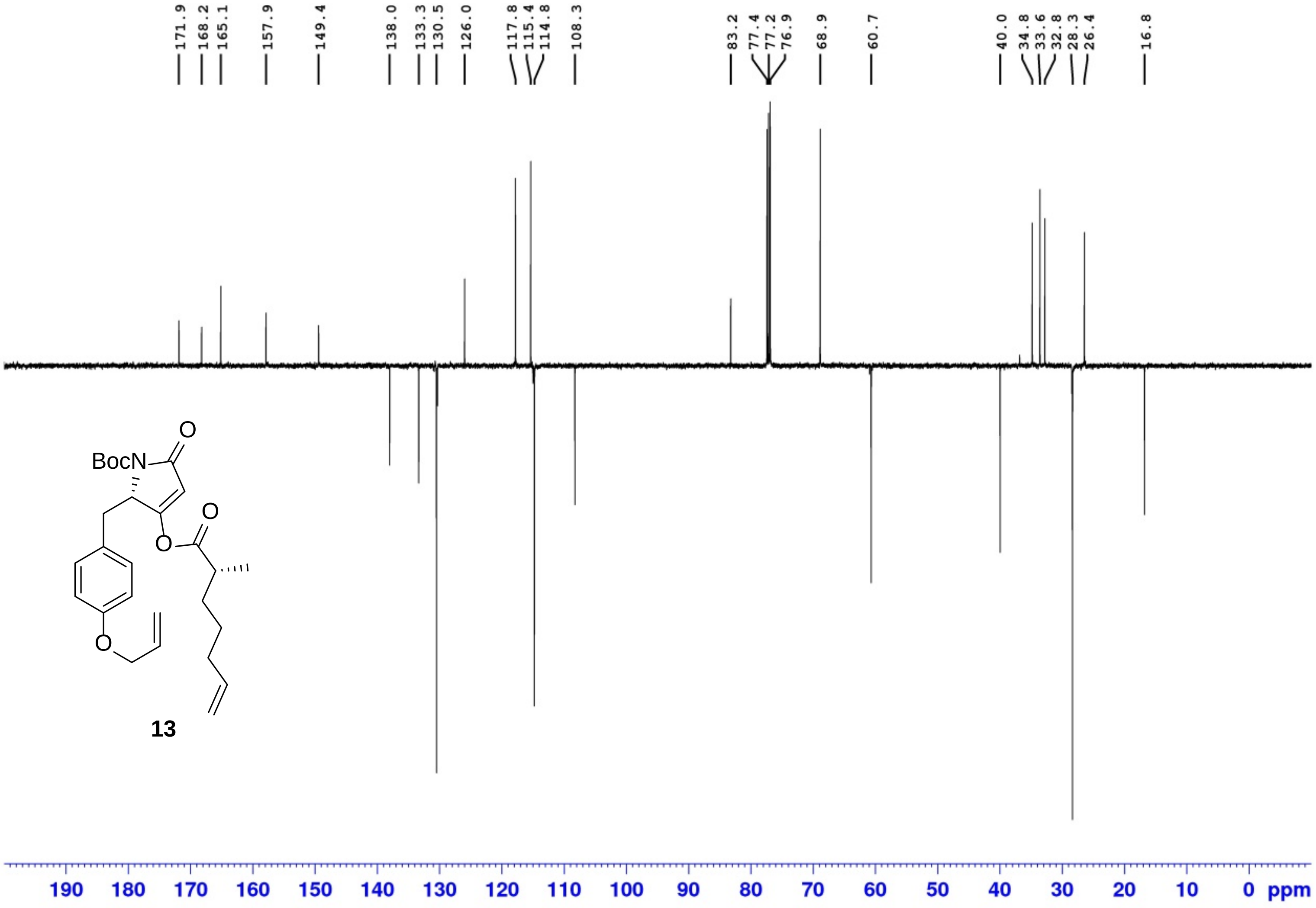

Figure S49: ^{13}C-NMR spectrum of compound **13** in $CDCl_3$.

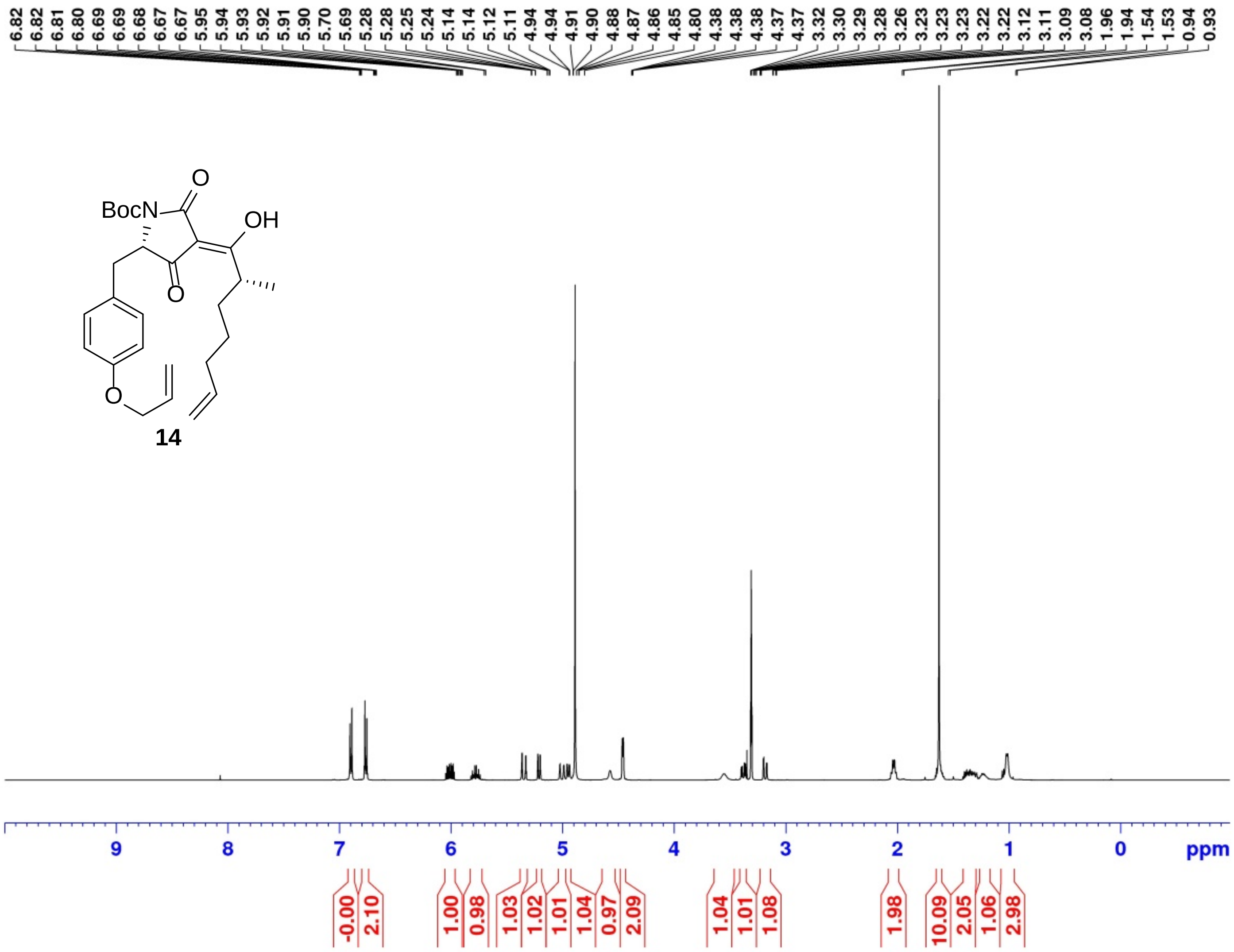

Figure S50: ^{1}H-NMR spectrum of compound **14** in MeOD.

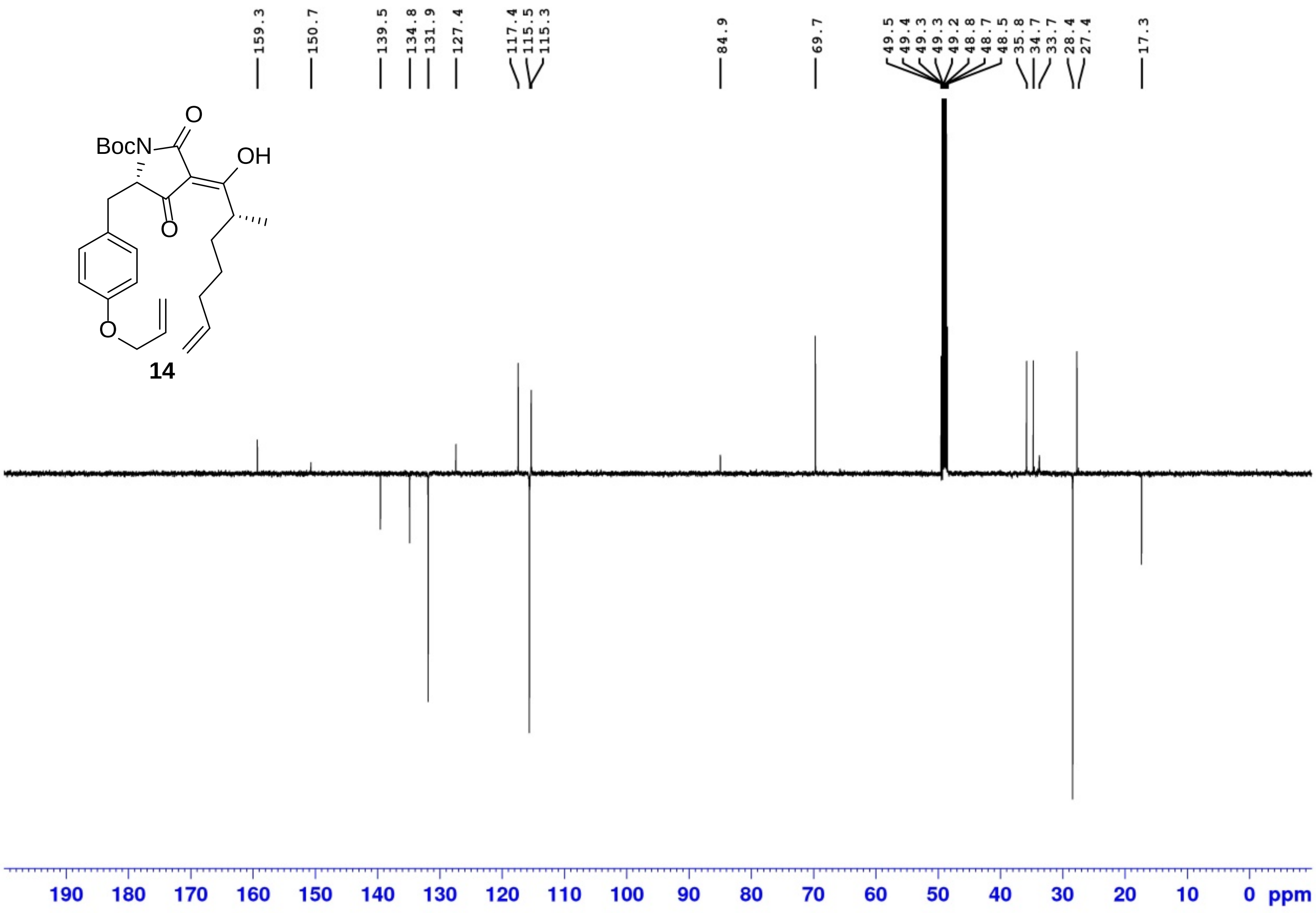

Figure S51: ^{13}C-NMR spectrum of compound **14** in MeOD.

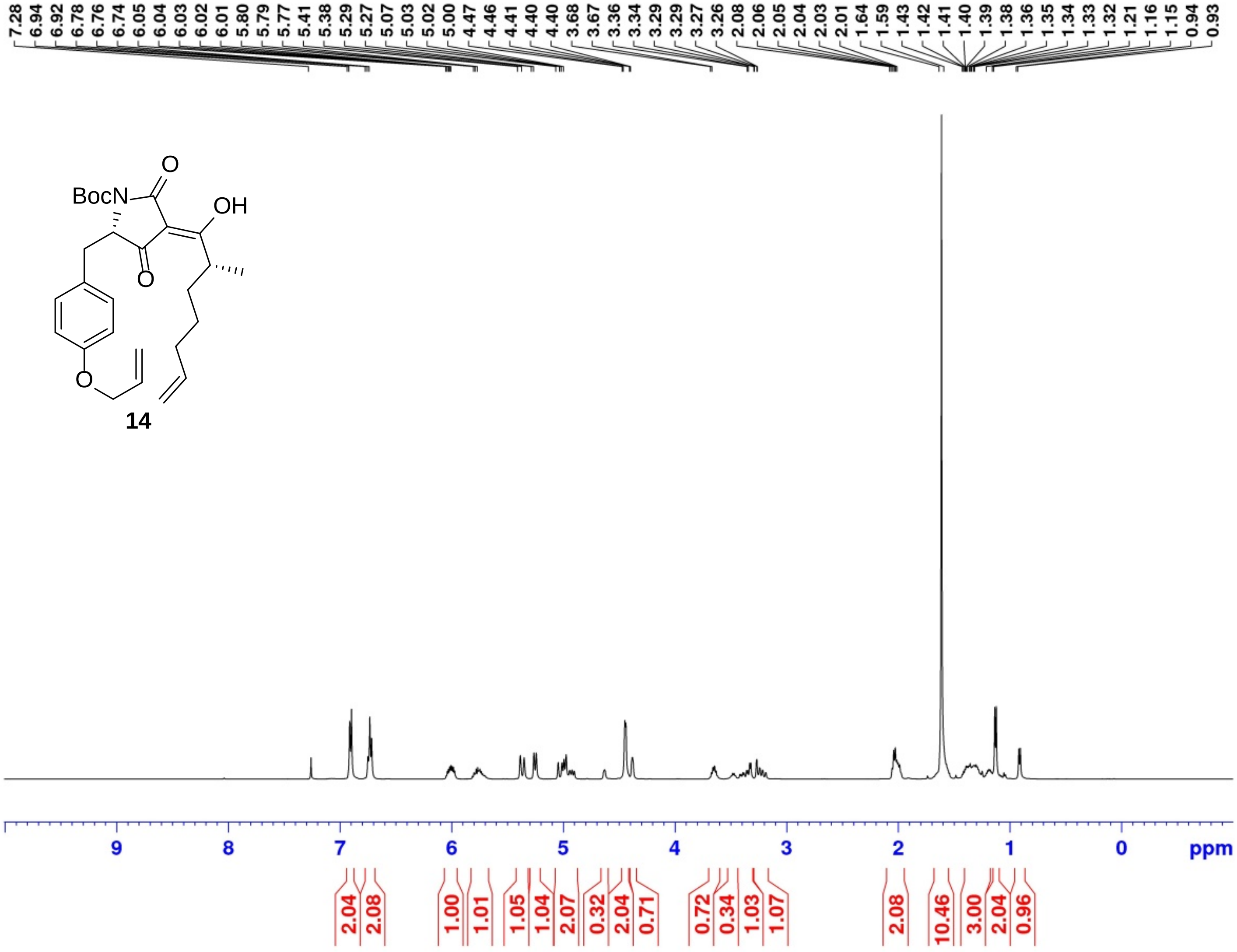

Figure S52: ^{1}H-NMR spectrum of compound **14** in $CDCl_3$.

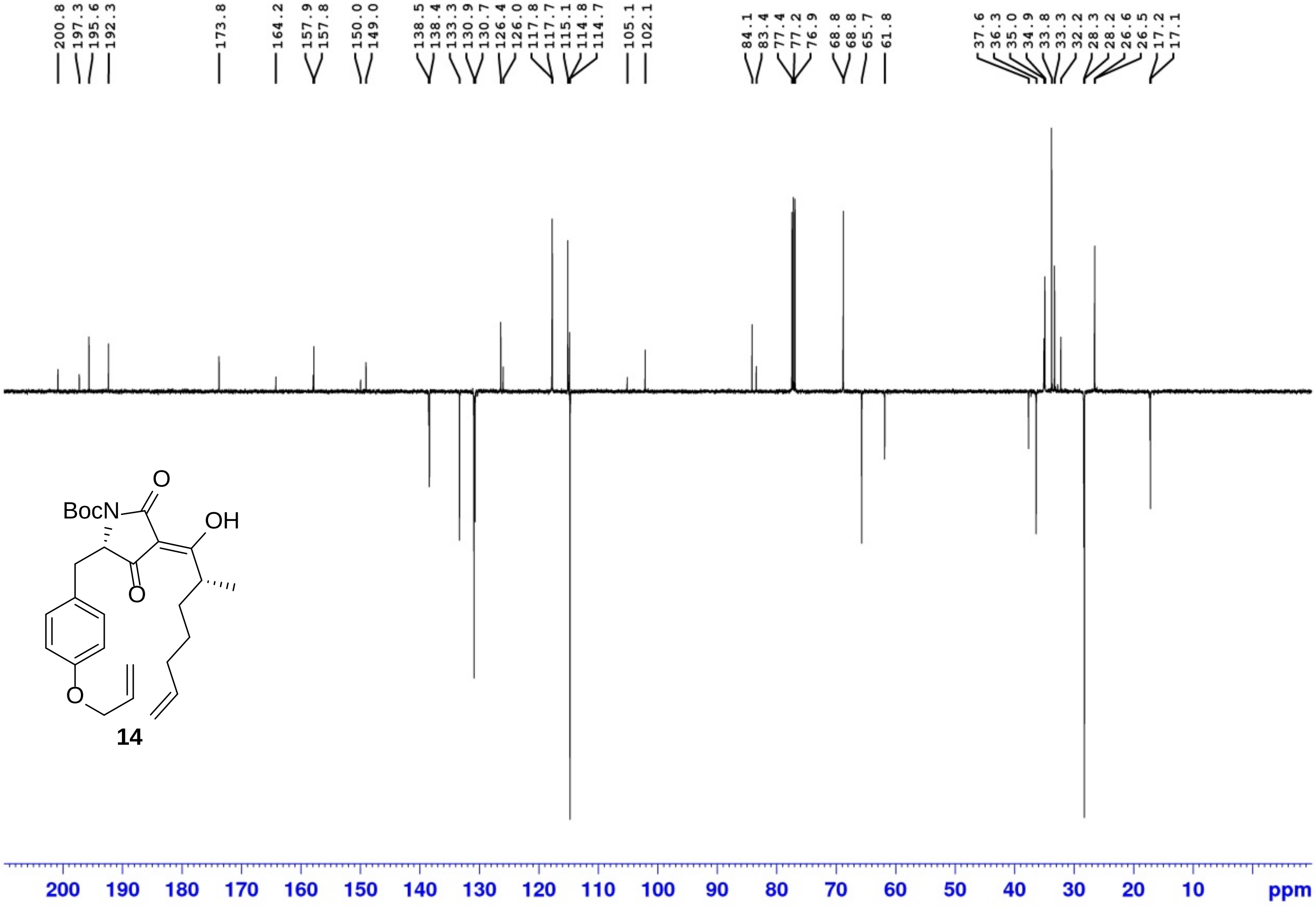

Figure S53: ^{13}C-NMR spectrum of compound **14** in $CDCl_3$.

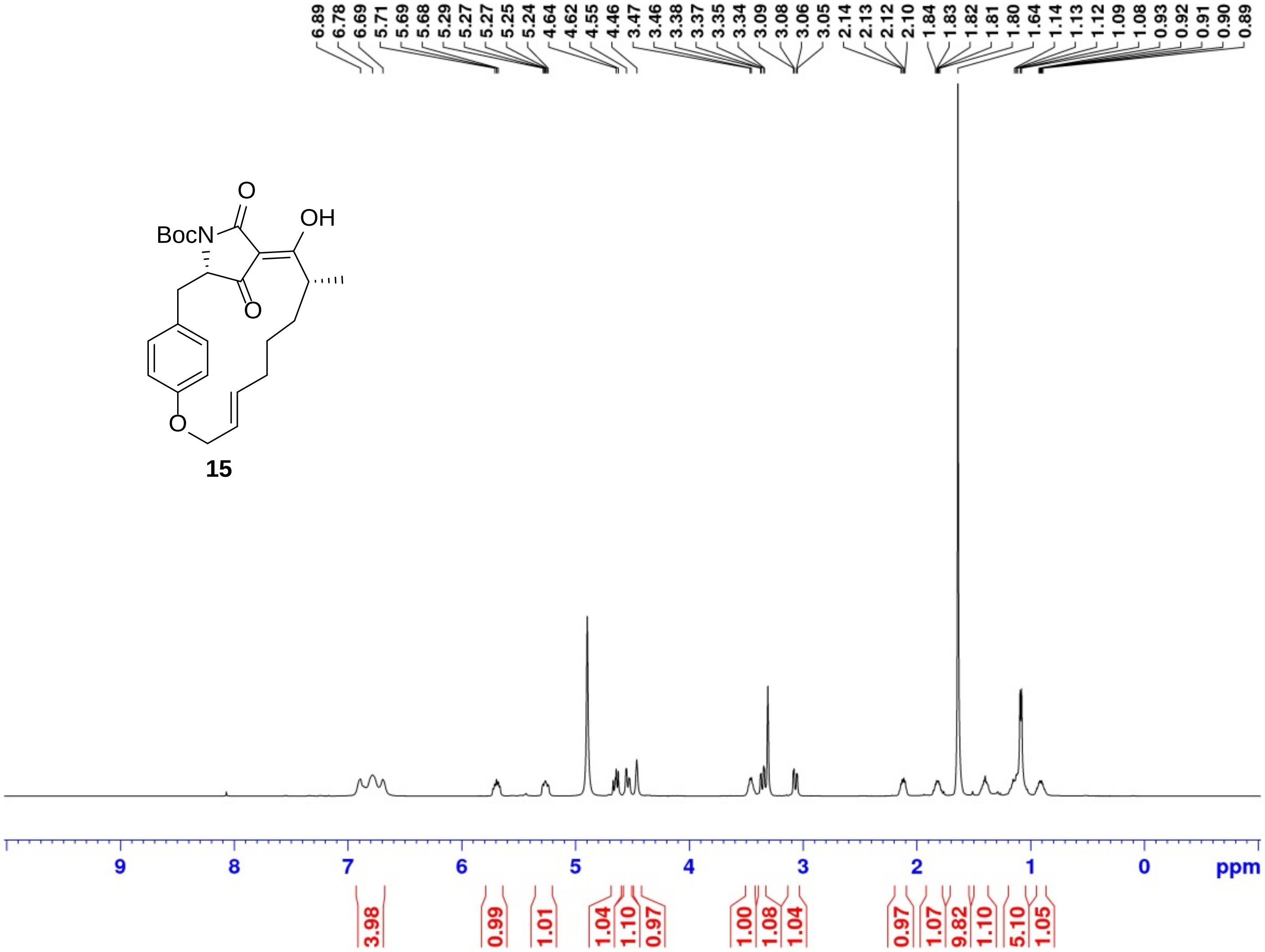

15

Figure S54: ^{1}H-NMR spectrum of compound **15** in MeOD.

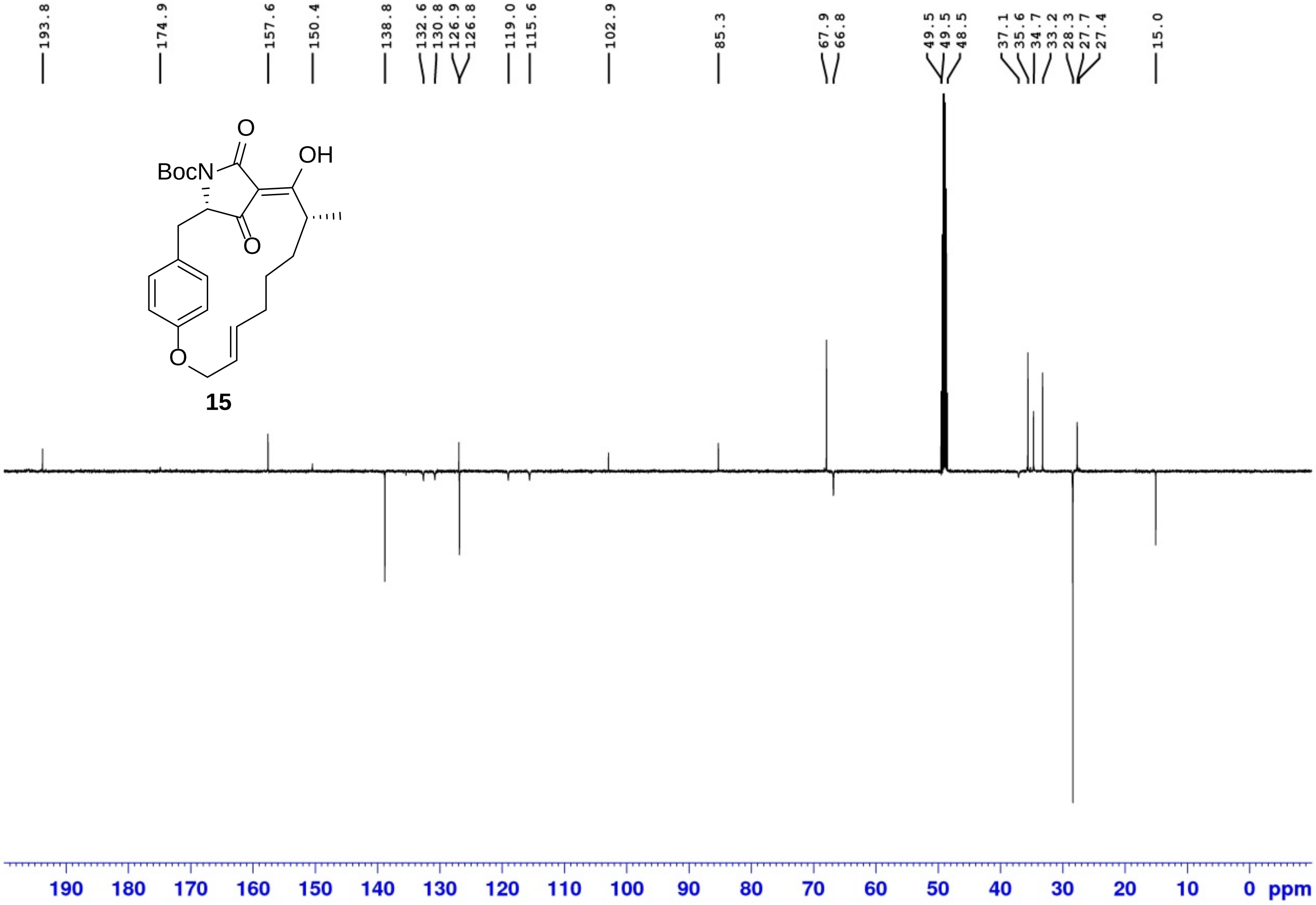

Figure S55: ^{13}C-NMR spectrum of compound **15** in MeOD.

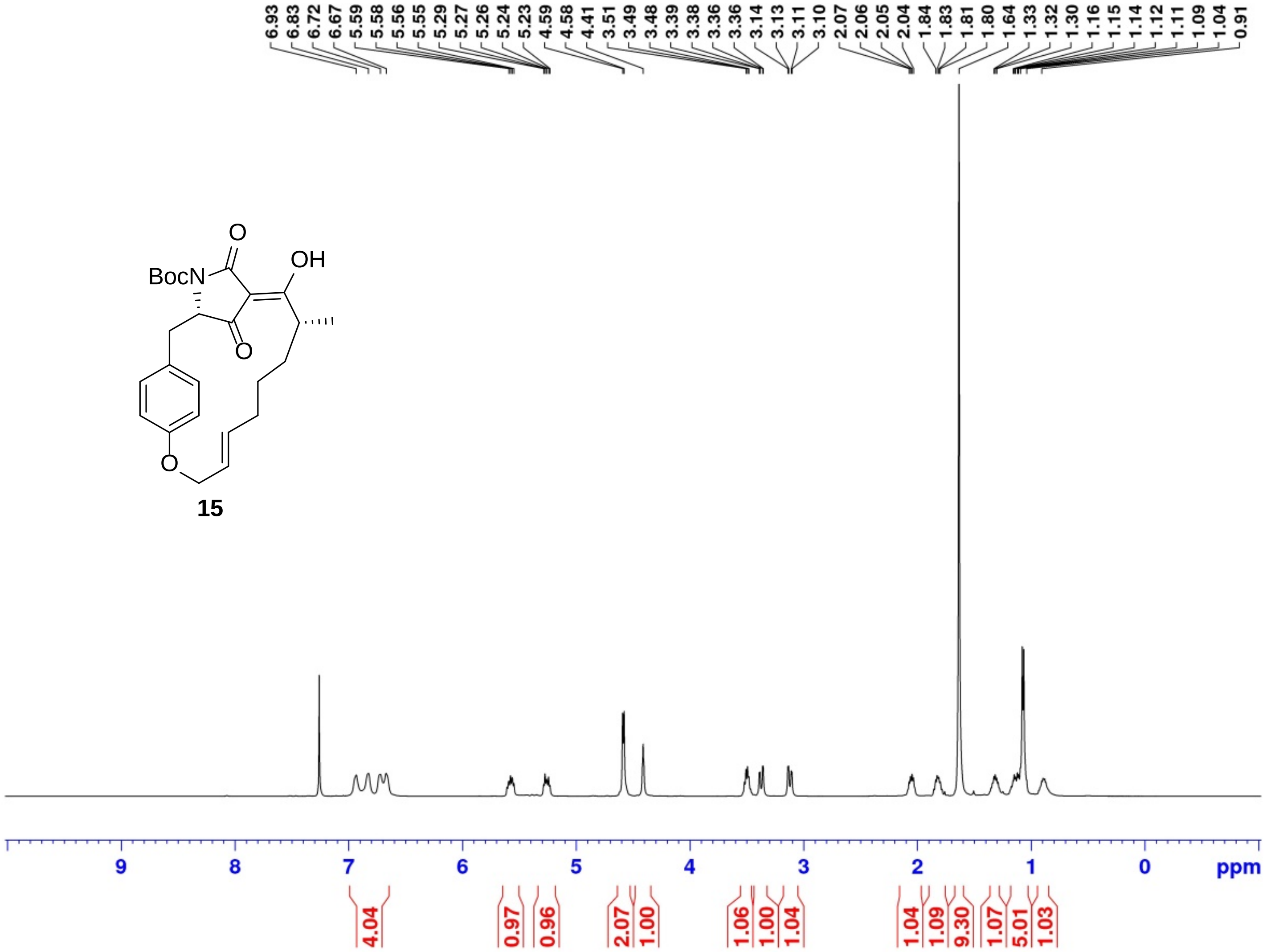

Figure S56: ^{1}H-NMR spectrum of compound **15** in $CDCl_3$.

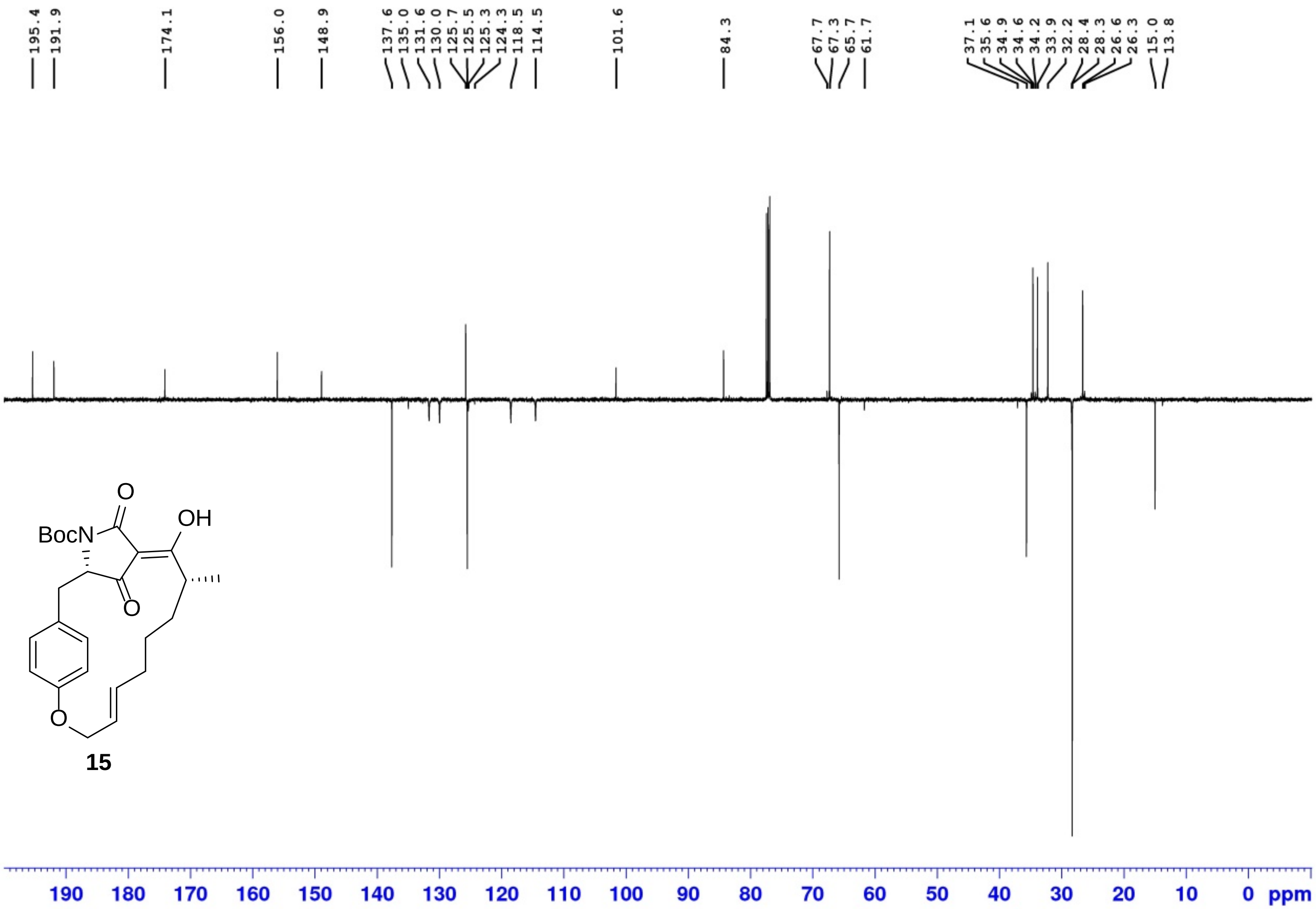

Figure S57: ^{13}C-NMR spectrum of compound **15** in $CDCl_3$.

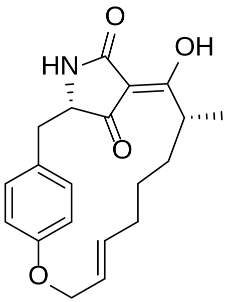

6

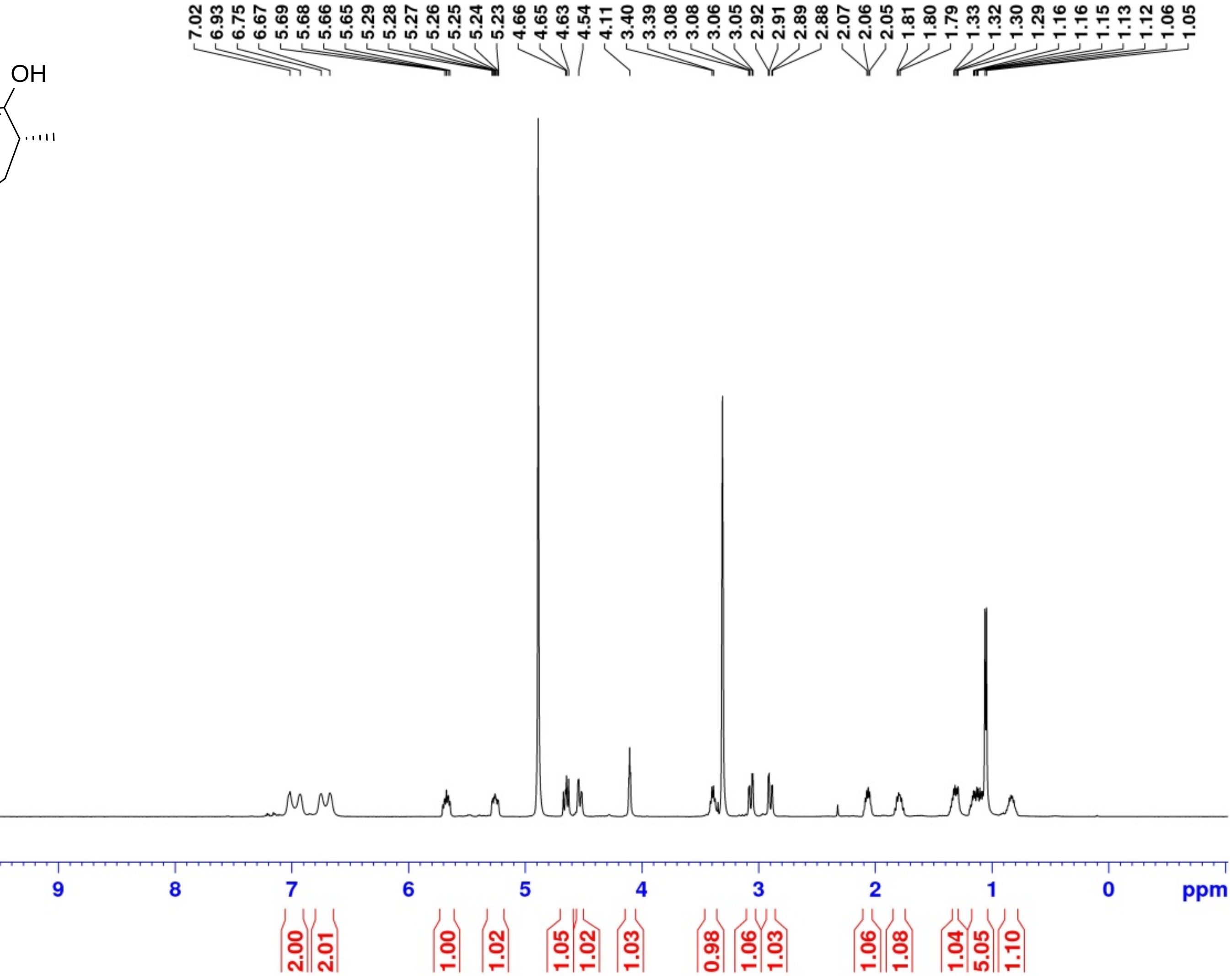

Figure S58: ^{1}H-NMR spectrum of macrocidin Z (**6**) in MeOD.

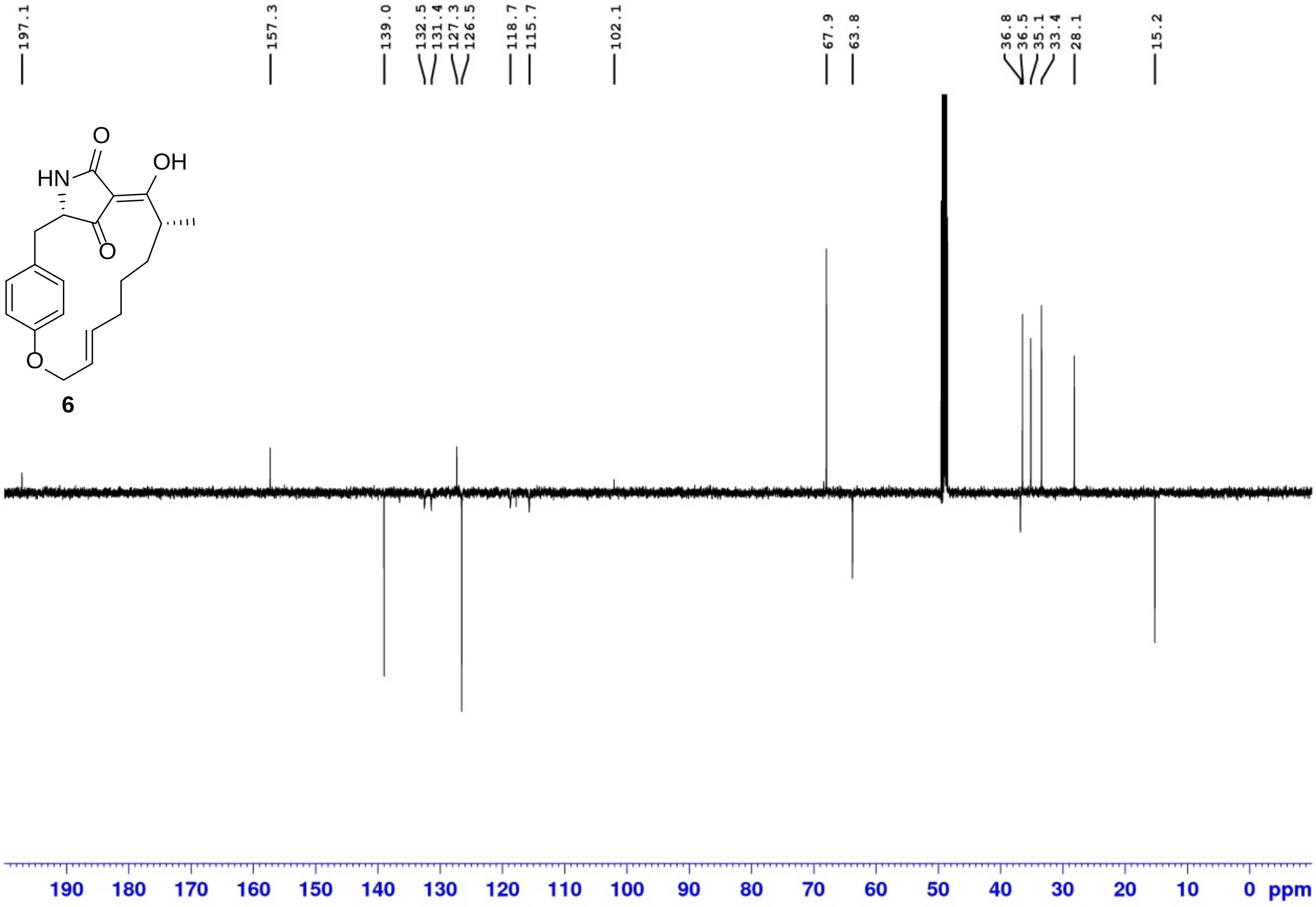

Figure S59: ^{13}C-NMR spectrum of macrocidin Z (**6**) in MeOD.

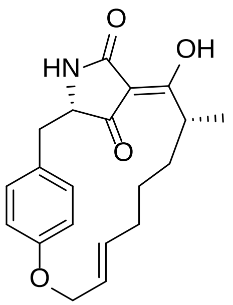

6

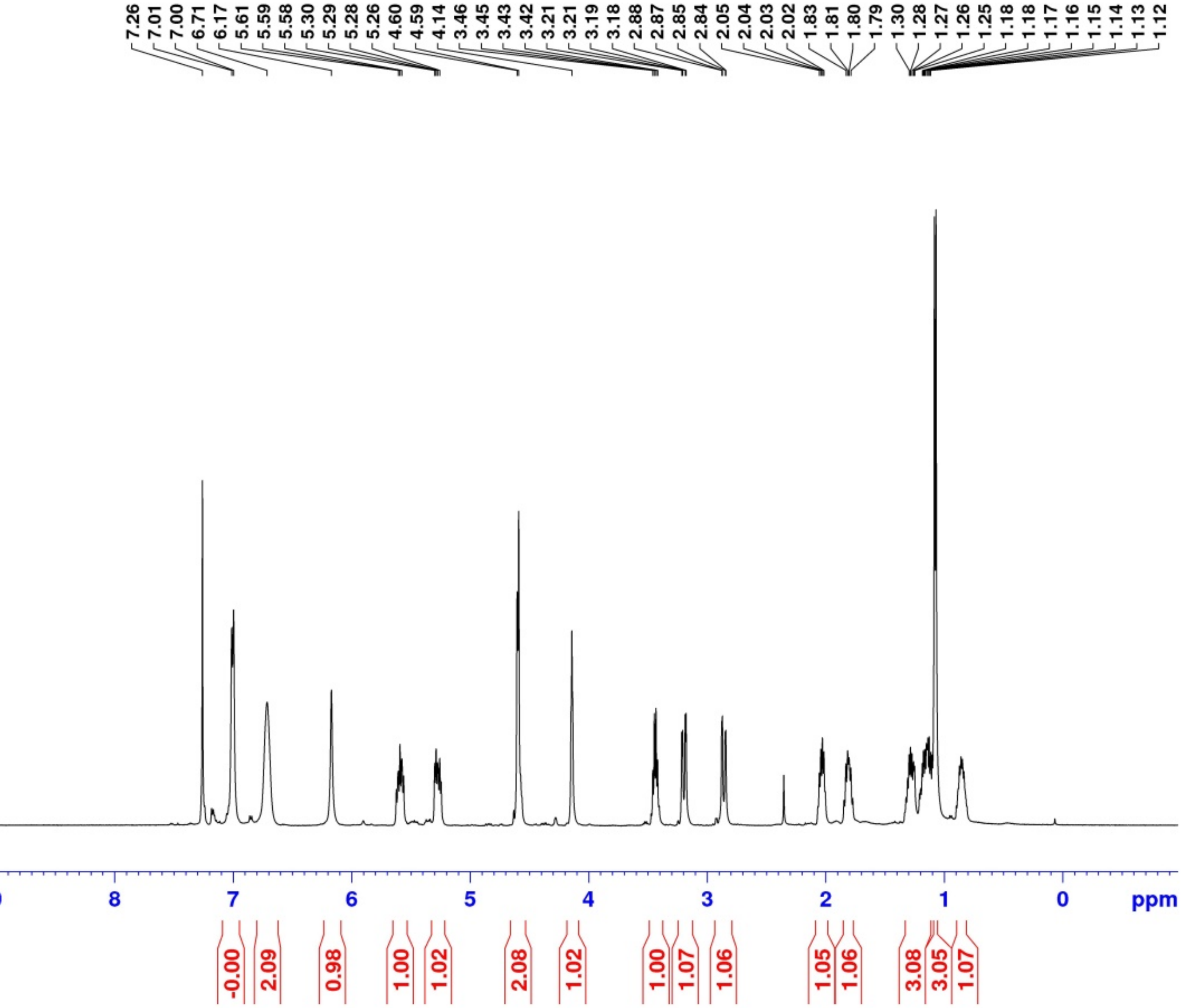

Figure S60: ^{1}H-NMR spectrum of macrocidin Z (**6**) in $CDCl_3$.

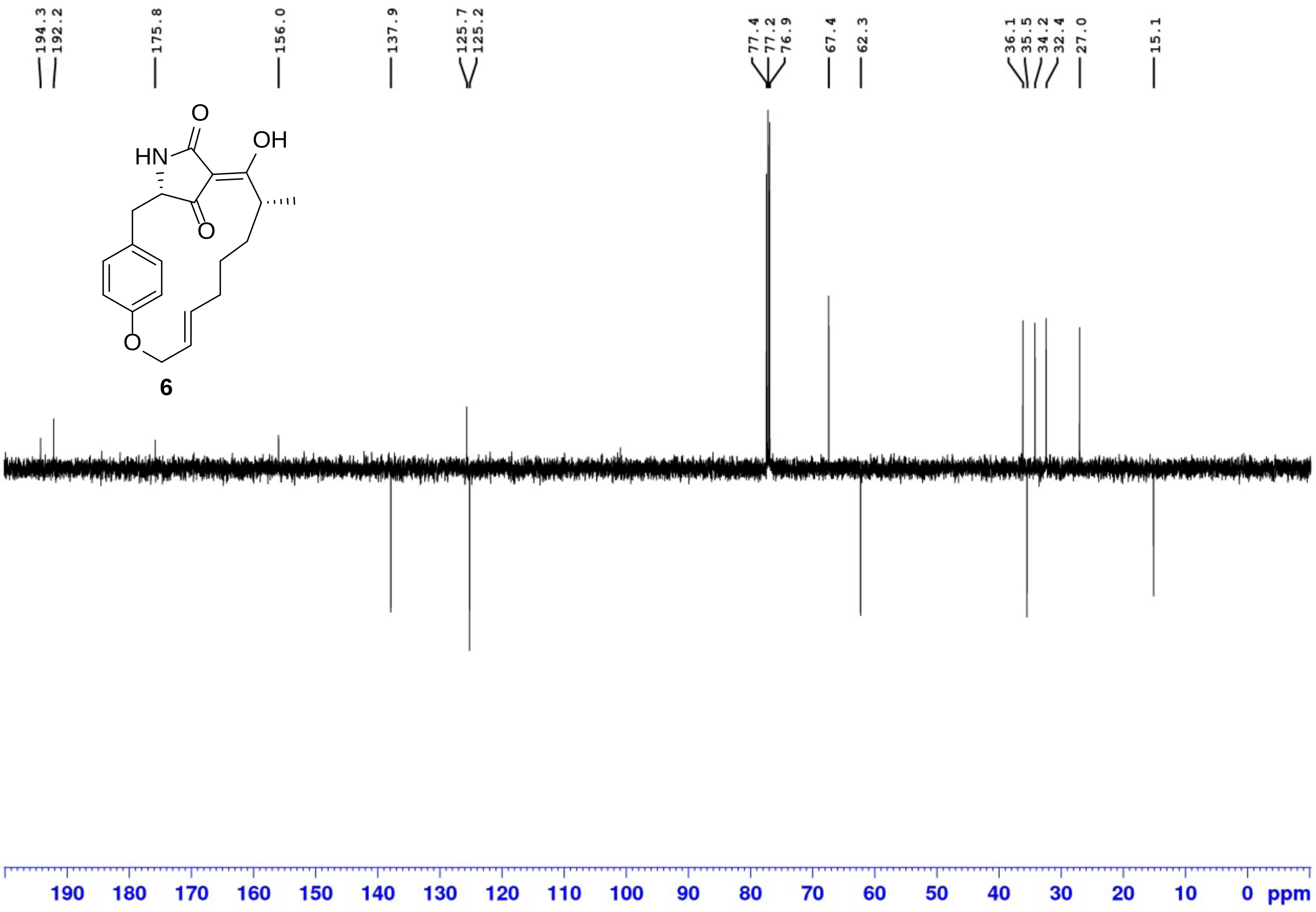

Figure S61: ^{13}C-NMR spectrum of macrocidin Z (**6**) in $CDCl_3$.

Antimicrobial assay

The assay was conducted as a minimum inhibitory concentration (MIC) assay in 96-well roundbottom microtiter plates using the parameters summarized in Table S3 and as already described in [S1].

Stocks of the test organisms were generated by growing the organisms overnight in 50 mL shaking flasks filled with 25 mL of the growth medium at 140 rpm (for media and temperatures see Table S3). If the organisms were well grown the next day, which was checked by occurrence of an optical density (OD)>30 of the suspension ($OD_{600\ nm}$ for bacteria, $OD_{548\ nm}$ for fungi and *M. smegmatis*), aliquots of these were stored in 1.5 mL reaction tubes in a freezer at −80 °C for up to 12 months. Upon use, aliquots were unthawed and the OD of the suspension measured and adjusted by diluting with the respective growth medium. $OD_{600\ nm}$ was adjusted to 0.01 and $OD_{548\ nm}$ to 0.1.

Subsequently, 150 µL of the adjusted suspensions were added to all wells of a 96-well microtiter plate (one test organism per plate). In row A, additional 130 µL of suspensions plus 20 µL of the test compounds (1 mg/mL) and the controls (one compound/column) were added. The test compounds were dissolved in MeOH, MeOH was used as negative control, while different positive controls (references) were used for the test organisms (see Table S3). Then, starting from row A, 150 µL of the suspension were transferred to the next row, the contents thoroughly mixed, and 150 µL transferred to the following row. The remaining 150 µL after row H were discarded. This resulted in a serial dilution of the test compounds, ranging from 66.7 µg/mL in row A to 0.52 µg/mL in row H. The microtiter plates were then incubated overnight on a microplate shaker at 800 rpm at 30 or 37 °C (see Table S3) and were visually evaluated the next day. The MIC is defined as the lowest concentration were no growth of the test organism was observed. A lower MIC thus corresponds to a higher antimicrobial activity of the test compound.

Table S3: MIC assay experiment parameters

Test organisms	Strain No.	Growth medium	Incubation temp. [°C]	Positive controls (references)
Bacillus subtilis	DSM10	MHB [1]	30	oxytetracyclin 1.0 mg/mL
Staphylococcus aureus	DSM346	MHB [1]	30	oxytetracyclin 1.0 mg/mL
Micrococcus luteus	DSM1790	MHB [1]	30	oxytetracyclin 1.0 mg/mL
Chromobacterium violaceum	DSM30191	MHB [1]	30	oxytetracyclin 1.0 mg/mL
Escherichia coli	DSM1116	MHB [1]	37	oxytetracyclin 1.0 mg/mL
Pseudomonas aeruginosa	PA14	MHB [1]	37	gentamicin 0.1 mg/mL
Mycolicibacterium smegmatis	ATCC700084	7H9+ADC [2]	37	kanamycin 0.1 mg/mL
Candida albicans	DSM1665	MYC [3]	30	nystatin 1.0 mg/mL
Schizosaccharomyces pombe	DSM70572	MYC [3]	30	nystatin 1.0 mg/mL
Mucor hiemalis	DSM2656	MYC [3]	30	nystatin 1.0 mg/mL

Pichia anomala	DSM6766	MYC [3]	30	nystatin 1.0 mg/mL
Rhodotorula glutinis	DSM10134	MYC [3]	30	nystatin 1.0 mg/mL

[1] MHB: Müller-Hinton Broth (SN X927.1, Carl Roth GmbH, Karlsruhe, Germany); [2] 7H9+ADC: Middlebrook 7H9 Broth Base + Middlebrook ADC Growth Supplement (SN M0678+M0553, Merck, Darmstadt, Germany); [3] MYC: 1 % w/v, bacto peptone, 1% w/v yeast extract, 2 % w/v glycerol, pH 6.3

[S1]: Becker, K.; Wessel, A. C.; Luangsa-ard, J. J.; Stadler, M. Viridistratins A-C, antimicrobial and cytotoxic benzo[j]fluoranthenes from stromata of Annulohypoxylon viridistratum (Hypoxylaceae, Ascomycota). Biomolecules 2020, 10, 805.

Cytotoxicity assay

The assay was conducted in 96-well flat-bottom microtiter plates using the parameters summarized in Table S4 and as described in [S1, S2].

Cell lines L929 and KB 3.1 were incubated at 37 °C under 10 % CO_2 in Gibco™ DMEM medium (Thermo Fisher Scientific, Waltham, MA, USA) supplemented with 10 % FBS. A microtiter plate was filled with 120 µL of this suspension (50,000/mL) in each well.

Separately, another microtiter plate was filled with 100 µL of growth medium in each well. Then, 50 µL of the test compound solutions (1 mg/mL) were given to wells of the first column in two replicates (one compound per row). Cells without additives, MeOH were used as negative control. Starting from the first column, 50 µL of the solutions were gradually transferred to the next column, the contents thoroughly mixed, and 50 µL transferred to the following column. This created a serial dilution of the test compounds ranging from 333 µg/mL to 1.9×10^{-3} µg/mL. The remaining 50 µL after column twelve were discarded. From this microtiter plate, 60 µL of the solutions from 111 µg/mL to 1.9×10^{-3}µg/mL were given to the first plate containing 120 µL of the cell suspensions (i.e. the highest concentration 333 µg/mL was not used). This resulted in final compound concentrations ranging from 37 µg/mL to 0.6×10^{-3} µg/mL.

After 5 days of incubation under the aforementioned incubation conditions, the half maximum inhibitory concentrations (IC50) were determined using a colorimetric tetrazolium dye MTT assay [S3]. For this, 20 µL of a 5 mg/mL solution of 3-(4,5-dimethyl-2-thiazolyl)-2,5-diphenyl-2H-tetrazolium bromide (MTT) were added to each well and incubated for two hours at 37 °C. Then, the microtiter plate was centrifuged (3,000 rpm, 5 min) and the supernatant removed by holding the plate upside-down and gentle shaking. Afterwards, the wells were washed using 100 µL of phosphate buffered saline (PBS). The plate was again centrifuged and the supernatant removed as described before. Then, 100 µL of an isopropanol:HCl solution (1L isopropanol+4 mL HCl 37 % w/v) were added to the wells. After incubating for 10 min at ambient temperature, the absorption of the wells at 595 nm was measured with an Infinite® 200 Pro microplate reader (TECAN, Männedorf, Schweiz).

The absorption values of the cells without additives were averaged and set to 100 % cell viability. Then, the means of absorption of the two compound replicates were set in relation to the blank media. These percentage values were plotted against the concentration range (37 µg/mL to 0.6×10^{-3} µg/mL). The IC_{50} value was read from the plot (in µg/mL).

Table S4: Cytotoxicity assay experiment parameters

cell line	type	No.	growth medium
L929	mouse fibroblasts	ACC 2	DMEM [1] + 10 % FBS [2]
KB 3.1	Human endocervical adenocarcinoma (AC)	ACC 158	DMEM [1] + 10 % FBS [2]

[1] DMEM: Dulbecco's Modified Eagle Medium (SN 61965026, Thermo Fisher Scientific, Waltham, MA, USA); [2] FBS: Fetal Bovine Serum (SN 10500064, Thermo Fisher Scientific)

[S2]: Sandargo, B.; Michehl, M.; Praditya, D.; Steinmann, E.; Stadler, M.; Surup, F. Antiviral meroterpenoid rhodatin and sesquiterpenoids rhodocoranes A–E from the Wrinkled Peach Mushroom, *Rhodotus palmatus*. *Organic Letters* **2019**, 21, 3286-3289.

Table S5: *S. aureus* biofilm and preformed biofilm inhibition activity of compounds **1-3**, **5-6**

**Compounds**	**Inhibition of biofilm formation (%)**	**Inhibition of preformed biofilm (%)**
**1**	-	-
**2**	65 (250 μg/mL) ± 5<br>43 (125 μg/mL) ± 9	36 (250 μg/mL)<br>31 (125 μg/mL)
**3**	75 (250 μg/mL) ± 3<br>59 (125 μg/mL) ± 9	57 (250 μg/mL) ± 3<br>48 (125 μg/mL) ± 15
**4**	n.t	n.t
**5**	79 (250 μg/mL) ± 2<br>77 (62.5 μg/mL) ± 2<br>61 (15.6 μg/mL) ± 15	75 (250 μg/mL) ± 4<br>65 (62.5 μg/mL) ± 12<br>31 (15.6 μg/mL) ± 13

**6**	76 (250 μg/mL) ± 9<br>70 (62.5 μg/mL) ± 11<br>19 (15.6 μg/mL) ± 8	73 (250 μg/mL) ± 7<br>59 (62.5 μg/mL) ± 10<br>40 (15.6 μg/mL) ± 15
Microporenic acid A	83 (250 μg/mL) ± 5<br>81 (62.5 μg/mL) ± 6<br>48 (15.6 μg/mL) ± 3	71 (250 μg/mL) ± 8<br>70 (62.5 μg/mL) ± 9<br>39 (15.6 μg/mL) ± 13

Data are expressed as mean ± SD.
